# Optical control of ultrafast structural dynamics in a fluorescent protein

**DOI:** 10.1038/s41557-023-01275-1

**Published:** 2023-08-10

**Authors:** Christopher D. M. Hutchison, James M. Baxter, Ann Fitzpatrick, Gabriel Dorlhiac, Alisia Fadini, Samuel Perrett, Karim Maghlaoui, Salomé Bodet Lefèvre, Violeta Cordon-Preciado, Josie L. Ferreira, Volha U. Chukhutsina, Douglas Garratt, Jonathan Barnard, Gediminas Galinis, Flo Glencross, Rhodri M. Morgan, Sian Stockton, Ben Taylor, Letong Yuan, Matthew G. Romei, Chi-Yun Lin, Jon P. Marangos, Marius Schmidt, Viktoria Chatrchyan, Tiago Buckup, Dmitry Morozov, Jaehyun Park, Sehan Park, Intae Eom, Minseok Kim, Dogeun Jang, Hyeongi Choi, HyoJung Hyun, Gisu Park, Eriko Nango, Rie Tanaka, Shigeki Owada, Kensuke Tono, Daniel P. DePonte, Sergio Carbajo, Matt Seaberg, Andrew Aquila, Sebastien Boutet, Anton Barty, So Iwata, Steven G. Boxer, Gerrit Groenhof, Jasper J. van Thor

**Affiliations:** 1 Department of Life Sciences, Faculty of Natural Sciences, Imperial College London, London, UK; 2 Diamond Light Source Ltd, Harwell Science & Innovation Campus, Didcot, UK; 3 Quantum Optics and Laser Science Group, Blackett Laboratory, Imperial College London, London, UK; 4 Department of Chemistry, Stanford University, Stanford, CA USA; 5 Physics Department, University of Wisconsin-Milwaukee, Milwaukee, WI USA; 6 Physikalisch Chemisches Institut, Ruprecht-Karls Universität Heidelberg, Heidelberg, Germany; 7 Nanoscience Center and Department of Chemistry, University of Jyväskylä, Jyväskylä, Finland; 8 Pohang Accelerator Laboratory, POSTECH, Pohang, Republic of Korea; 9 Department of Chemical Engineering, POSTECH, Pohang, Republic of Korea; 10 RIKEN SPring-8 Center, Sayo, Hyogo Japan; 11 Institute of Multidisciplinary Research for Advanced Materials, Tohoku University, Sendai, Miyagi Japan; 12 Department of Cell Biology, Graduate School of Medicine, Kyoto University, Sakyo, Kyoto Japan; 13 Japan Synchrotron Radiation Research Institute, Sayo, Hyogo Japan; 14 Linac Coherent Light Source, Stanford Linear Accelerator Centre (SLAC), National Accelerator Laboratory, Menlo Park, CA USA; 15 Center for Free-Electron Laser Science, Deutsches Elektronen-Synchrotron, Hamburg, Germany

**Keywords:** Photobiology, Nanocrystallography, Reaction kinetics and dynamics, Reaction mechanisms, Thermodynamics

## Abstract

The photoisomerization reaction of a fluorescent protein chromophore occurs on the ultrafast timescale. The structural dynamics that result from femtosecond optical excitation have contributions from vibrational and electronic processes and from reaction dynamics that involve the crossing through a conical intersection. The creation and progression of the ultrafast structural dynamics strongly depends on optical and molecular parameters. When using X-ray crystallography as a probe of ultrafast dynamics, the origin of the observed nuclear motions is not known. Now, high-resolution pump–probe X-ray crystallography reveals complex sub-ångström, ultrafast motions and hydrogen-bonding rearrangements in the active site of a fluorescent protein. However, we demonstrate that the measured motions are not part of the photoisomerization reaction but instead arise from impulsively driven coherent vibrational processes in the electronic ground state. A coherent-control experiment using a two-colour and two-pulse optical excitation strongly amplifies the X-ray crystallographic difference density, while it fully depletes the photoisomerization process. A coherent control mechanism was tested and confirmed the wave packets assignment.

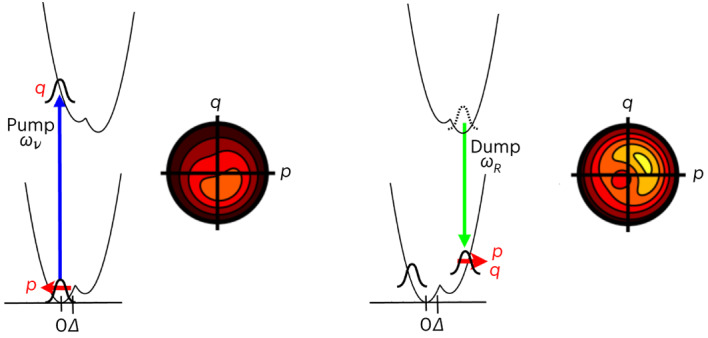

## Abstract (web summary)

Pump–probe measurements conventionally achieve femtosecond time resolution for X-ray crystallography of reactive processes, but the measured structural dynamics are complex. Using coherent control techniques, we show that the ultrafast crystallographic differences of a fluorescent protein are dominated by ground-state vibrational processes that are unconnected to the photoisomerization reaction of the chromophore.

## Main

Photoisomerization of chromophores in fluorescent proteins (FPs)^1^, bacteriorhodopsin^2,3^ and photoreceptors, such as rhodopsin^4,5^, phytochrome^6,7^ or the photoactive yellow protein^8–12^, involve specific excited-state bond rearrangements in addition to macromolecular reaction dynamics and non-adiabatic transitions at conical intersections. Biological photoisomerization is a representative example of non-adiabatic reaction dynamics that involves substantial nuclear reconfiguration. The class of reversibly photoswitchable FPs (rs-FPs) is of particular interest for understanding the origin of structural motions that are associated with the *cis–trans* and *trans–cis* photoisomerization reactions of these biological chromophores. The photoisomerization reaction of rs-FPs includes incoherent excited-state barrier crossing^13^. These rs-FPs have therefore found widespread use in the fields of protein highlighting^14^, optogenetics^15^ and super-resolution microscopy^16^. The protein environment in rs-FPs controls the isomerization process through specific electrostatic interactions^13^, and direct structural observations are therefore essential to understand how these interactions steer the ultrafast process.

Because photoisomerization occurs on the timescale of the excited-state (ES) lifetime, ultrafast time resolution is needed for direct structural observations. The non-adiabatic dynamics occur either coherently within the vibrational dephasing time^17^ or incoherently through thermally driven barrier crossing within the excited-state lifetime^18^. In the latter case, excited-state motion may not contribute substantially to measurements made with short observation delays, because the product accumulates thermally by barrier crossing throughout the excited-state lifetime. The photoisomerization motion is therefore not directly captured in a pump–probe time series, which measures concentration changes instead if the process is incoherent. With the advent of X-ray free-electron laser (XFEL) sources, recording time-resolved X-ray crystallographic structures of such ultrafast photochemical processes has become a reality^12,19–22^. Successful time-resolved crystallography studies have used the pump–probe approach, and the light-induced X-ray crystallographic difference densities have been assigned to reactions and excited-state processes. However, the relationship between ultrafast nuclear dynamics that are measured in real space directly from crystallographic coordinates and the outcome of the non-adiabatic dynamics is not yet established. This is because there remain fundamental open questions regarding the correct physical assignment and interpretation of the observed ultrafast structural changes. First, analysis based on rate kinetics that quantifies concentration changes of static species is commonly applied to time-resolved observations, but this is not applicable if the time resolution is within vibrational dephasing. Second, although it is widely accepted in the field of Raman spectroscopy that both ground-state and excited-state motions contribute to the structural dynamics under conditions of ultrafast excitation^23–26^, the extent of contributions from electronic ground-state motions has not yet been analysed for experimental time-resolved serial femtosecond crystallography (TR-SFX) results and has so far only been considered on a theoretical basis^26,27^. In this Article, to address these open questions, we demonstrate the use of optical control for analysis of the creation and evolution of coherence under conditions for ultrafast TR-SFX.

**Fig. 1:**
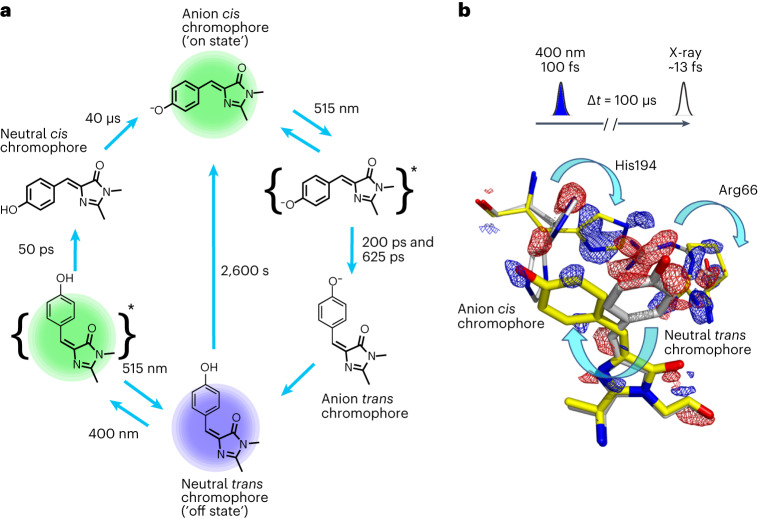
rsKiiro photocycle. **a**, General photocycle scheme of the reversible photoisomerization and proton-transfer reactions of rsKiiro. Light-induced transitions with 400-nm (blue) and 515-nm (green) wavelengths are indicated. **b**, Time-resolved crystallography measurement of *trans*–*cis* photoisomerization of the off state with a *trans* neutral chromophore using femtosecond excitation at a wavelength of 400 nm, conducted at PAL-XFEL. *Q*-weighted *F*_o(100μs)_–*F*_o(Dark)_ difference maps contoured at 3*σ* at a resolution of 1.5 Å and at a 100-μs delay show *trans*–*cis* photoisomerization and rearrangements of His194 and Arg66.

**Extended Data Fig. 1:**
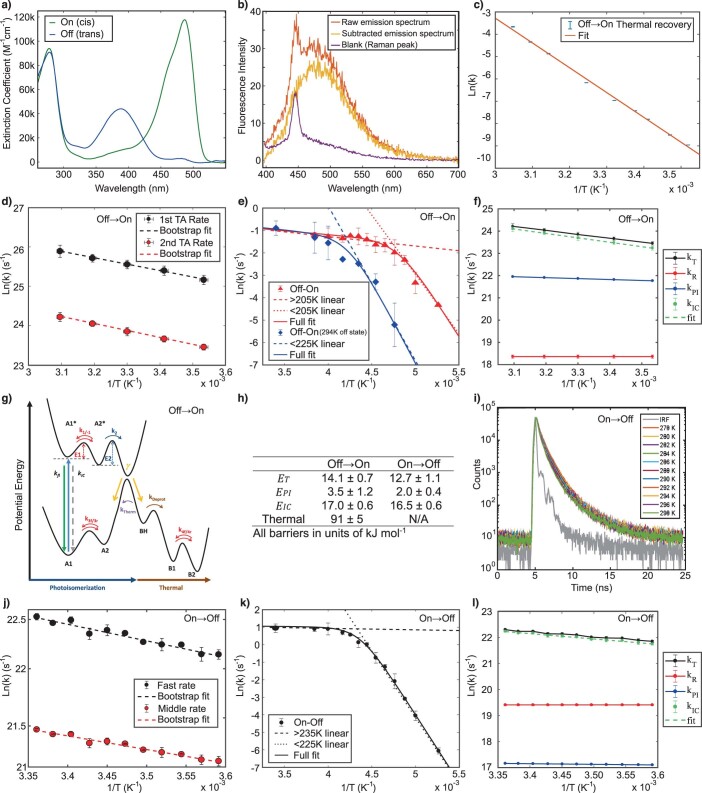
Temperature dependence and energy barriers of rsKiiro photo-switching. **a**. Steady state UV -VIS spectra of the on (cis) and off (trans) state. **b**. Fluorescence emission spectra from the weakly fluorescent off state. **c**. Arrhenius plot of the off→on thermal recovery at temperature s ranging from 284-323 K fitting of the Arrhenius equation recovered an energy barrier of of 91± 5 kJ mol^-1^ between the states and an unscaled prefactor of 1.28×10^13±1^ s^-1^. **d**. Arrhenius plot of the temperature dependence of the two off→on PP TA time constants (Extended Data Fig. 3d), **e**. Arrhenius plot of the off→on photoswitching rate over a large range of cryo temperatures, where the preconversion to the off state was performed at cyro (red) and room temperature (blue). **f**. Bootstrap Arrhenius fitting of the total off→on rate (k_T_) obtained from temperature dependence of the average TA rate which was used with the photoswitching scaled rate (k_PI_) and radiative decay rate (k_R_) to recover the internal conversion rate (k_IC_) and barrier (E_IC_). **g**. The proposed electronic structure model for the off→on reaction of rsKiiro. **h**. Summery table of electronic barriers recovered for rsKiiro photoswitching reactions. **i**. Time correlated single photon counting (TCSPC) decays for the on→off reaction of rsKiiro including the instrument response function (IRF). **j**. Arrhenius plots of the temperature dependence of the fast and middle rates recovered from TCSPC. **k & l** The same plots as e & f for the on→off reaction. A full description of all the fitting models and values are shown in section 11 of the supplementary materials. Error bars shown in **c-f** & **j-l** use SE.

**Extended Data Fig. 2:**
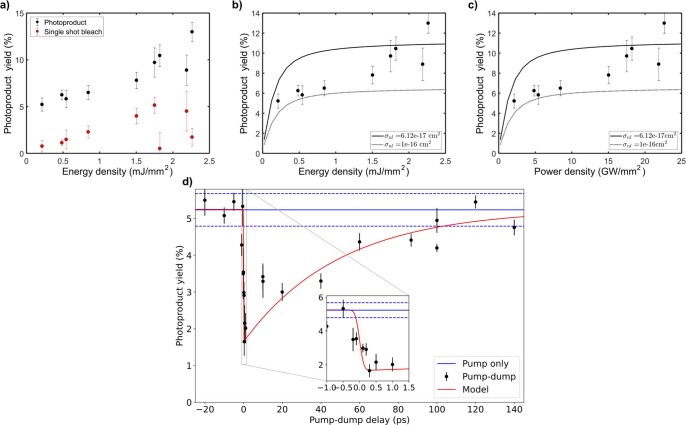
Flash photolysis yields in crystalline rsKiiro with femtosecond pumping. **a**. Photoproduct yield and single shot bleach of rsKiiro prepared in the trans off state and pumped with 400 nm 100 femtosecond pulses over a range of energy densities. **b**. Fitting of total non-linear cross-section modelled with Z-scan theory using a quantum yield of 18.3% for the off→on reaction (section 6 of supplementary materials). **c**. Same fitting expressed against power density. **d**. Photoproduct yield in crystalline rsKiiro in the presence of a 515 nm dump pulse (black) as a function of pump-dump delay. The pump-probe yield (blue) and its error (blue dashed) are shown for reference, while the model (red) is the convolution of two 100 fs Gaussian pulses combined with the 50 ps exponential decay of the excited state lifetime obtained from transient absorption measurements. In **a-d** each measurement condition was repeated across numerous crystalline samples with absorptions ranging between 0.1-0.6 OD. Error bars shown use SE.

**Extended Data Fig. 3:**
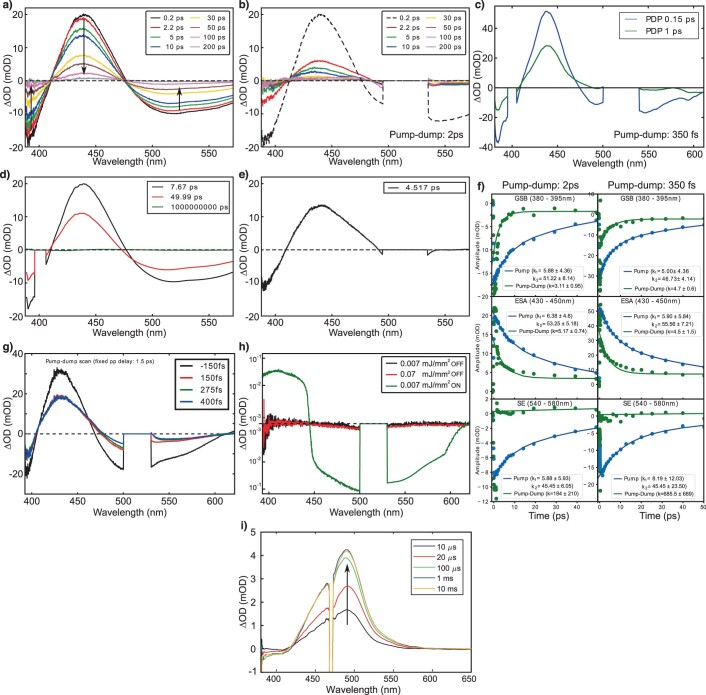
Transient absorption spectroscopy of rsKiiro. **a**. PP transient absorption spectra showing GSB, ESA and SE at <410, 420-470 and 470-570 nm. **b**. PDP transient absorption spectroscopy for a 2 ps pump-dump delay, showing a ~50% reduction of the GSB and ESA and complete suppression of the SE. **c**. PDP TA spectra for pump-dump delay of 350 fs showing the same behaviour as the longer dump delay. **d**. Evolution Associated Difference Spectra (EADS) recovered from global fitting of PP TA spectra using a simple sequential model (A1*→A2*→A2/A1/BH)(Extended Data Figs. 1g and 8). **e**. Global fitting of the PDP TA spectra after the dump pulse recovered a single EADS corresponding to the A2 state. **f**. Lineout fits of the PP and PDP key spectra regions for 2 ps (left) and 350 fs (right) pump-dump delays, a bi-exponential was used for the PP fits with amplitude weighting and initial time constant taken from the global fitting. **g**. TA spectra for a fixed pump-probe delay of 1.5 ps while scanning the pump-dump delay. **h**. Dump only off-resonance pumping TA spectra with 510 nm femtosecond excitation of the ‘off’ state at fixed 1 ps pump probe delay (‘off’ spectra). Preillumination with 515 nm light prepares the pure off state and no TA signals are seen within the sensitivity of the instrument (black and red) while if the preillumination is not used and the ON state is present the weaker condition generates >100 mOD signals (Note: log vertical scale). This demonstrates that 515 nm dump pulse has sufficiently low cross section in the off state to not contribute to the signals seen in the absence of a pump-pulse. **i**. Microsecond transient absorption difference spectra of rsKiiro trans-cis reaction. The rise 488 nm peak corresponding to the cis-anion can be seen with a 40 ± 10 μs time constant. The break in the spectra at 473 nm corresponds to scatter of the pre-illumination laser.

**Extended Data Fig. 4:**
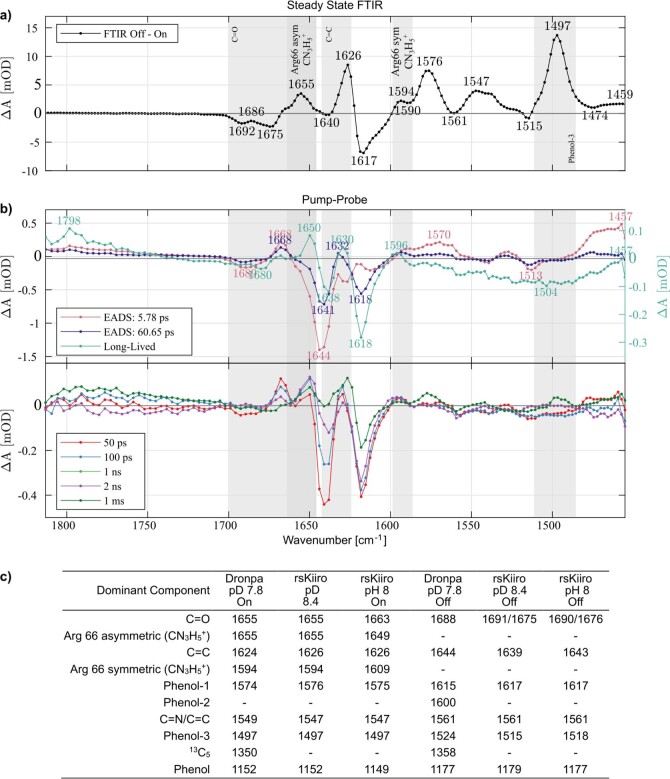
Femtosecond time resolved Infrared and FTIR spectroscopy of rsKiiro. **a**. steady state FTIR difference spectra of the off minus the on state of rsKiiro. Conversion to the off state was achieved by illuminating the sample with a 500 nm LED. **b**. TR-IR pump-probe measurements of the off state when pumped with 400 nm 100 fs pulses. The fitted EADS spectra are shown (top) and selected spectra at various pump-probe delays (bottom). Greyed out bands correspond to the labelled assignments. Note: For the on state there is overlap of the C = O and Arg66 asym CN_3_H_5_^+^ in the 1645-1670 cm^-1^ region. **c**. Dronpa and rsKiiro on and off state FTIR peak assignments for ^2^H and ^1^H buffers. Dronpa assignments made by Warren et al. All values in units of cm^-1^.

Based on the EosFP sequence^28^, we developed a new reversibly photoswitching rs-FP, ‘rsKiiro’ (described in detail in Supplementary Discussion 10). Reversible photoswitching occurs between a bright fluorescent *cis* anionic *p*-hydroxybenzylideneimidazolinone (HBDI) chromophore (the ‘on’ state) and the non-fluorescent *trans* neutral (the ‘off’ state) (Fig. 1), as is also the case for the rs-FP ‘Dronpa’^14^. Compared to Dronpa, rsKiiro has substantially improved structural ordering in the ‘on’ ground state as well as the ‘off’ photoproduct, allowing high-resolution X-ray crystallography of the photoreactions. In addition, rsKiiro was selected by screening variants for photoisomerization quantum yield, thermal recovery kinetics, complete photoconversion and full reversibility (Extended Data Figs. 1c and 2a and Supplementary Discussion 10). Illumination of the ‘on’ state with green light drives a *cis–trans* photoisomerization of the chromophore to produce a metastable non-fluorescent ‘off’ state with a neutral *trans* chromophore (Fig. 1a). The thermal-ground-state recovery of the ‘on’ state at ambient temperature is sufficiently slow for pump*–*probe TR-SFX, with *τ*_rev_ ≈ 2,600 s (Fig. 1), and reversible *trans–cis* photoisomerization driven by 400-nm illumination proceeds to completion with a quantum yield of ~0.2, enabling detection with a sufficient concentration of photoproduct (Fig. 1b). Transient visible (Extended Data Fig. 3) and mid-infrared (Extended Data Fig. 4) spectroscopy confirm the excited-state *trans–cis* photoisomerization with a pump-induced 12% yield in solution samples and crystals (Extended Data Fig. 2 and Supplementary Methods 6).

We conducted high-resolution ultrafast TR-SFX experiments of the ‘off’ state of rsKiiro under conditions designed to optically control vibrational coherence and population dynamics. Four experiments conducted at LCLS, PAL-XFEL and SACLA yielded a consistent and reproducible set of observations that revealed a fundamental new understanding of the origins of ultrafast structural dynamics in this photochromic rsKiiro FP. Two complementary sets of experiments were conducted, namely a pump*–*probe (PP) experiment and a two-pulse, two-colour pump*–*dump*–*probe (PDP) experiment, to control the excited- and ground-state motions.

## Results and discussion

An ~100-fs pump interaction at 400 nm, close to resonance of the *trans* neutral chromophore (off state), creates an excited-state population with a lifetime of *τ* = 50 ps in both solution and crystals (Fig. 1a and Extended Data Figs. 1a, 2 and 3d). A power density of 1,400 GW cm^−2^ (full-width at half-maximum, FWHM) was chosen for PP experiments to maximize the photoisomerization yield, based on optical measurements in crystals of anionic *cis* product absorption (Extended Data Fig. 2a). Under these conditions, a TR-SFX experiment shows that *trans*–*cis* photoisomerization is associated with short-range motions of His194 and Arg66 in the chromophore binding pocket (Fig. 1b). With the 100-μs PP delay, the formation of the on-state product has completed, which includes deprotonation of the phenolate chromophore (Fig. 1a). These observations match the static photoinduced differences between the on- and off-state crystal structures prepared with weak continuous-wave (c.w.) illumination (Supplementary Fig. 48).

**Fig. 2:**
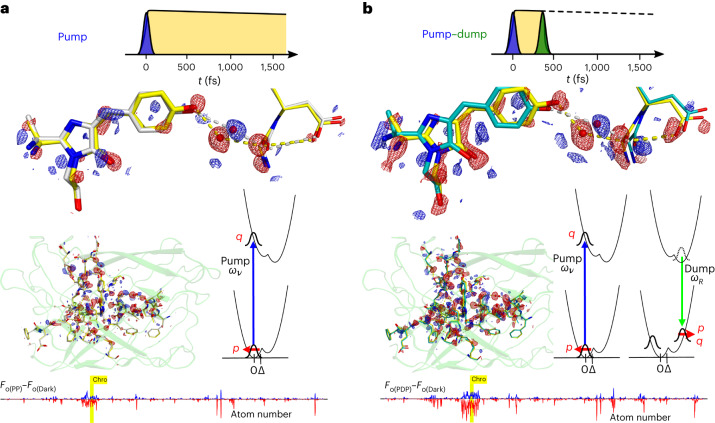
Optical control of structural dynamics in rsKiiro. **a**,**b**, Femtosecond time-resolved PP (**a**) and PDP (**b**) TR-SFX experiment for rsKiiro in the off state with a *trans* neutral chromophore. For this analysis, time-resolved data with delays between ~250 fs and 1.2 ps were merged, and *Q*-weighted *F*_o(PP)_–*F*_o(Dark)_ and *F*_o(PDP)_–*F*_o(Dark)_ maps are shown (red, −3*σ*, blue +3*σ*) at a resolution of 1.5 Å. Coordinates for the ground state are shown (yellow sticks; PDB 7QLM). Coordinates for the PP data (white sticks, **a**; PDB 7QLN) and PDP data (cyan sticks, **b**; PDB 7QLO) were refined from extrapolated structure factors and occupancy refinement. The creation of coherence in the PP and PDP conditions follows the density matrix theory of impulsive Raman spectroscopy applied to a double-well adiabatic potential. The initial Wigner coordinate and direction relative to the nuclear binding force (momentum, *p*; position, *q*) are indicated by arrows. Also shown are the sequentially integrated electron densities around each atom in the protein chain (bottom), with the atoms in the chromophore highlighted in yellow.

**Extended Data Fig. 5:**
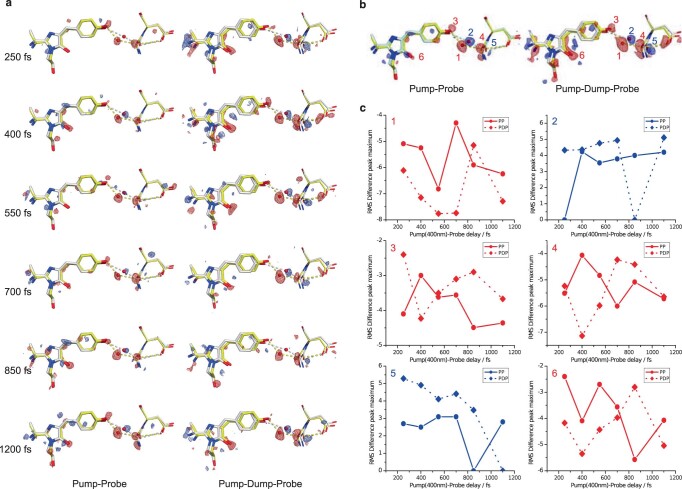
Comparison of the PP vs PDP TR-SFX light induced differences in the rsKiiro chromophore region. **a**. Difference maps for the chromophore region, hydrogen bonded water and His194 for the femtosecond time bins for PP (Left) and PDP (right) data. The coordinates for ground state (yellow carbons) and the refined coordinates for light data after partiality refinement (grey carbons) are shown with electron density contoured at +3σ rms (blue) and -3σ rms (red).A 1.5 Å resolution cutoff was used. **b**. Same representation for the merged 0-1 ps PP delays with key difference peaks numbered. **c**. Difference density peak values at the locations shown in panel b. Plotted as a solid line (with dots) and a dashed line (with diamonds) for the PP and PDP respectively. Shown in blue for positive values of density, and in red for negative. The time dependence of difference features elsewhere in the protein (Fig. 1) showed a similar multi-mode character, but with different dependence for most features.

**Extended Data Fig. 6:**
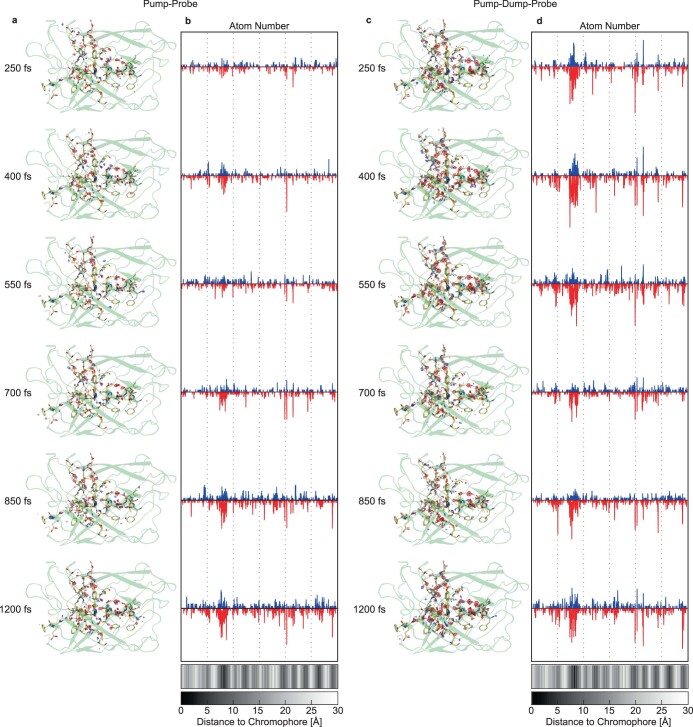
Comparison of rsKiiro TR-SFX Pump-Probe vs Pump-Dump-Probe difference maps. **a**. Q-weighted difference electron density for each bin of the Pump-Probe (400 nm) on the left and of the Pump-Dump-Probe (400-515 nm) on the right. The secondary structure (green) of the ground state coordinates (yellow) are shown with electron density contoured at +3σ rms (blue) and -3σ rms (red). **b**. Integrated electron difference density above 3σ level within 2 Å of each sequential atom in the protein chain (in residue number order) for Pump-Probe data 3375set. Plotted for each time bin is the positive (blue) and negative (red) electron density, which are normalized with respect to all other time bins. Shown at the bottom is a greyscale bar indicating the distance from the center of the chromophore to the atom number^65^ **c**. and **d**. The same analysis for the Pump-Dump-Probe case.

**Extended Data Fig. 7:**
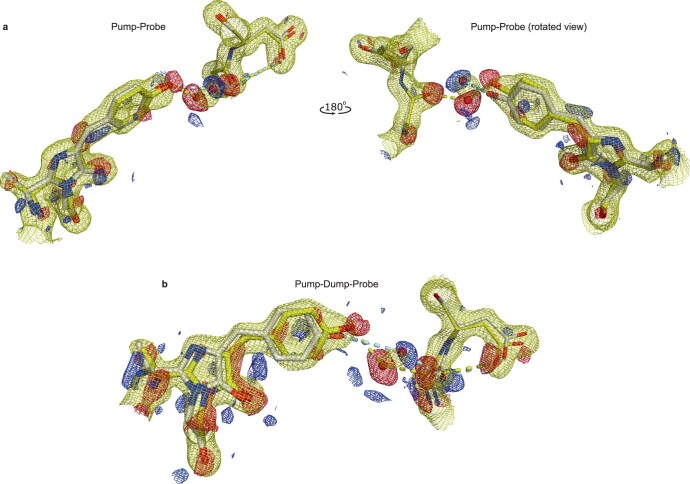
Extrapolated maps and difference maps of merged 0-1 ps TR-SFX of rsKiiro with refined coordinates. **a**. Pump-probe illumination scheme. Map showing extrapolated F_0_ electron density for N_EXT_ = 13.6, contoured at 1.5 rms (yellow mesh) with the ground state (yellow) and extrapolated (grey) refined coordinates. Q-weighted difference electron density between the dark and 400 nm 0-1 ps is shown at 3 rms level with positive and negative signals in blue and red respectively. There is a 180° rotation between the left and right representations. **b**. Pump-Dump-Probe illumination scheme, with same representation except a value of N_EXT_ = 9 was chosen as it was closest to R-factor minimization occupancies of 21 % using the approximation: PT ~ 200/ N_EXT_ where PT is population transfer.

Femtosecond TR-SFX experiments at LCLS achieved data at a resolution of 1.35 Å (Table 1), and timing tool measurements allowed binning of diffraction data into 150-fs-wide bins (Extended Data Figs. 5 and 6). The pump–probe observations up to 1-ps delay showed strong *F*_o_–*F*_o_ difference electron-density features, up to 8.5*σ*, on the chromophore and on a hydrogen-bonded water and throughout the core of the protein (Fig. 2a and Extended Data Fig. 7). Coordinate refinement from extrapolated structure factors and occupancy refinement showed a small translational motion of the chromophore characterized by a 69-pm displacement of the phenolic oxygen. A 1.25-Å coordinate change of a nearby hydrogen-bonded water molecule modifies the O–O distance between the chromophore and water from 2.63 Å to 3.06 Å (Fig. 2a). A concomitant repositioning of the Gly155 backbone carbonyl changes its O–O distance to this water molecule from 2.77 Å to 2.84 Å. The larger distances in the femtosecond time-resolved structure suggest that the hydrogen bonding of the water molecule to both the chromophore and Gly155 is weakened. Other water molecules in the chromophore pocket and the side chains that primarily reside on the central helix undergo similar rearrangements (Fig. 2a). Separation of the ultrafast time-resolved PP data into bins of 150 fs showed complex dynamical changes of these difference-electron-density features throughout the protein, including those on the chromophore and hydrogen-bonding environment (Extended Data Fig. 5c). The dynamics are interpreted as a superposition of multiple impulsively driven modes that follow the displacement, rather than a single periodic motion. The displacements of protein, chromophore and water molecules, as shown in Fig. 2, were already detected in the early ~250–400-fs time bin. The time dependence of the dynamical modulation of difference features is limited by the ~4.4-THz experimental bandwidth, which is determined by the experimental pulse durations.

**Table 1:** LCLS LR23 PP and PDP crystallographic statistics for merged 0–1-ps delays

	Dark	400 nm, 0–1 ps	400–515 nm, 0–1 ps
**Data collection**			
Wavelength (eV)	9,486	9,486	9,486
Indexed patterns	35,030	47,590	39,754
Resolution limits (Å)	15.40–1.80 (1.864–1.800)^a^	15.40–1.80 (1.864–1.800)^a^	18.01–1.80 (1.864–1.800)^a^
	15.40–1.50 (1.554–1.500)^b^	15.40–1.50 (1.554–1.500)^b^	18.01–1.50 (1.554–1.500)^b^
	15.40–1.35 (1.398–1.350)^c^	15.40–1.35 (1.398–1.350)^c^	18.01–1.35 (1.398–1.350)^c^
No. of unique reflection indices	21,941^a^	21,941^a^	21,964^a^
	37,542^b^	37,542^b^	37,565^b^
	51,229^c^	51,229^c^	51,252^c^
No. of merged reflections	8,859,960 (583,183)^a^	9,663,635 (454,993)^a^	7,042,698 (265,101)^a^
	12,622,674 (790,070)^b^	11,562,142 (263,229)^b^	7,928,580 (96,156)^b^
	14,971,721 (647,523)^c^	11,990,599 (64,419)^c^	8,056,732 (13,369)^c^
Completeness (%)	100.00 (100.00)^a^	100.00 (100.00)^a^	99.96 (100.00)^a^
	100.00 (100.00)^b^	100.00 (100.00)^b^	99.98 (100.00)^b^
	100.00 (100.00)^c^	99.77 (97.73)^c^	96.67 (68.35)^c^
Signal to noise	8.568 (5.24)^a^	9.793 (5.65)^a^	8.272 (4.27)^a^
	6.328 (1.86)^b^	7.225 (2.36)^b^	5.790 (1.20)^b^
	4.843 (0.40)^c^	5.607 (0.61)^c^	4.566 (0.88)^c^
Wilson *b* factor (Å^2^)	27.23^a^	13.84^a^	16.96^a^
	32.89^b^	16.13^b^	19.19^b^
	35.68^c^	16.60^c^	19.87^c^
*R*_Split_ (%)	9.27 (18.03)^a^	8.76 (16.90)^a^	9.83 (22.54)^a^
	9.51 (79.05)^b^	9.83 (40.27)^b^	11.19 (82.62)^b^
	9.68 (291.14)^c^	11.01 (142.12)^c^	12.19 (350.70)^c^
CC*	1.00 (0.98)^a^	1.00 (0.99)^a^	1.00 (0.98)^a^
	1.00 (0.61)^b^	1.00 (0.94)^b^	1.00 (0.82)^b^
	1.00 (0.07)^c^	1.00 (0.62)^c^	1.00 (N/A)^c^
CC_1/2_	0.99 (0.94)^a^	0.99 (0.94)^a^	0.99 (0.91)^a^
	0.99 (0.23)^b^	0.99 (0.79)^b^	0.99 (0.51)^b^
	0.99 (0.00)^c^	0.99 (0.24)^c^	0.99 (0.05)^c^
**Refinement**			
Resolution (Å)	1.3/1.5	1.3/1.5	1.3/1.5
No. of reflections used (all/free)	51,401/35,668	51,120/35,645	47,319/35,676
Reflections used for *R*-free	2,754/1,828	2,671/1,851	2,460/1,833
*R*-factor	0.1836/0.1529	0.1922/0.1674	0.1984/0.1785
*R*-free	0.1901/0.1628	0.1946/0.1700	0.2055/0.1837
No. of non H atoms	2,074	2,074	2,074
Protein residues	216	216	216
Bonds (Å)	0.0288	0.0598	0.0589
Angles (°)	2.767	2.854	3.014

^a,b,c^Merged statistics for distinct resolution shells with resolution cut-off at (a) 1.80 Å, (b) 1.50 Å and (c) 1.35 Å. The values in parenthesis are given for the highest resolution shells.

A two-pulse, two-colour PDP experiment was executed in which the delay between the pump (400 nm) and the Stokes (515 nm) ‘dump’ pulses was chosen to be 350 fs (Fig. 2b). Under these conditions, the electronic dephasing following the first interaction has completed, and coherent impulsive Raman processes are therefore not driven by the two-colour excitation^29^. At the chosen pump–dump delay of 350 fs, the Stokes ‘dump’ pulse selectively and fully depletes the S_1_ excited state and generates a vibrationally excited electronic-ground-state population. The complete loss of the S_1_ excited state in the PDP conditions is confirmed from spectrally resolved transient absorption (TA) spectroscopy in solution (Extended Data Fig. 3b–g) and the loss of photoisomerization product in crystals (Extended Data Fig. 2).

**Extended Data Fig. 8:**
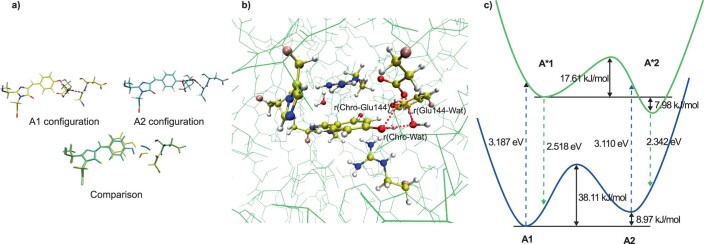
Results of QM-MM calculations on rsKiiro. **a**. Hydrogen bonding configuration in state A1 (yellow), A2 (cyan) and an overlap of the two configurations. **b**. QM subsystem used for QM-MM calculations, shown with balls and sticks. Red dashed lines represent distances, used to search for transition state between A1 and A2 conformations. **c**. Energy diagram for optimized structure of A1 and A2 conformations from QM-MM calculations. These calculations are described in section 12 of the supplementary materials.

The PDP measurements were collected in an interleaved manner together with the PP data, and consistently showed significantly stronger but otherwise almost identical *F*_o_–*F*_o_ electron-density differences as compared to the PP data (Fig. 2b). In addition to amplification of the light-induced differences, the time dependence of the dynamics was also modified when the dump pulse was included (Extended Data Fig. 5c). Structure refinement of the PDP data averaged to 1-ps delay resulted in coordinate changes very similar but with larger displacement compared to the PP data (Fig. 2b and Extended Data Fig. 7). These structural rearrangements suggest a double-well adiabatic potential in both the electronic ground (S_0_) and excited (S_1_) states. The 1.25-Å coordinate change of the water seen in the PP and PDP differences (Fig. 2a,b), as well as the magnitude of other rearrangements, is too large to consider displacement for a single oscillator^27^. The double-well nature of the adiabatic potential of the S_1_ state was experimentally confirmed from the temperature dependence of excited-state decay of the off state as well as the on state. In addition, structure-based thermodynamics modelling retrieved the barriers associated with the double-well potentials for the radiative and non-radiative transitions of the on and off states (Extended Data Fig. 1 and Supplementary Section 11). This is also in line with hybrid quantum mechanics/molecular mechanics (QM/MM) geometry optimizations of the protein in S_0_ and S_1_ (Extended Data Fig. 8 and Supplementary Section 12). The QM/MM simulations furthermore suggest that the relative energies of the minima of these double-well potentials interchange between the ground and excited states (Fig. 2).

We consider the premise that if a time-resolved signal contains both excited- and ground-state contributions, then, after selectively depleting S_1_ by stimulated emission pumping, the signals belonging to the excited state should disappear and only the ground-state signals should remain. Here we observe the same structures in both PP and PDP experiments. However, the PDP data show stronger differences and a modified temporal dependence compared to the PP measurement after the Stokes ‘dump’ pulse was introduced, and there are no discernible signals that are depleted by stimulated emission pumping. This observation forms our primary motivation for assigning the structural changes observed in the PP conditions to dominating ground-state motion, which is unconnected to the photoisomerization reaction coordinate. This assignment shows that the ground-state nuclear coherence, which is impulsively driven^24,25^, dominates the measured displacements. The excited-state coherence is not impulsively prepared; it is purely displacement-driven and insufficiently ordered, whereas the *cis* anionic chromophore product state is seen after full electronic and population decay and thermal proton transfer (Fig. 1). It is concluded that ground-state, rather than excited-state, motions dominate the ultrafast measurements, whereas the product state is only detected with longer waiting times (Fig. 1).

The direct correspondence between the structural changes observed in the PP and PDP experiments prompted further investigation into the electronic origin of the measured femtosecond motions. An ultrafast electronic TA study with 400-nm excitation of the neutral *trans* chromophore (off state) produces induced absorption at 440 nm and a broad stimulated emission spanning 460–600 nm. Including a Stokes ‘dump’ pulse at 515-nm wavelength and with FWHM power density of 2,800 GW cm^−2^ at either 350-fs or 2-ps delay after the pump fully depletes the stimulated-emission profile across the entire spectral width (Extended Data Fig. 3b–g). Strikingly, the Stokes ‘dump’ pulse reduces the induced absorption feature to approximately half the original amplitude, prompting assignment of the 440(+)-nm band to a combination of excited-state absorption and induced absorption of an electronic-ground-state intermediate. Fitting of this feature provides an ~4.5-ps time constant for its subsequent decay for samples in solution (Extended Data Fig. 3e,g).

Furthermore, ultrafast PDP optical measurements in crystal samples confirmed the complete depletion of photoisomerization (Extended Data Fig. 2d). A scan of the arrival time of the Stokes ‘dump’ pulse relative to the pump between negative time and the full excited-state decay showed the expected cross-correlation in the rise time of the action spectroscopy and was consistent with the 50-ps excited-state decay (Extended Data Figs. 1c and 2a). This is conclusive evidence that *trans*–*cis* photoisomerization proceeds from the S_1_ singlet excited state via thermal barrier crossing, and is not a ground-state process as previously suggested^30^. The action spectroscopy additionally indicates that the isomerization is not vibrationally coherent as shown for rhodopsin^17^, but proceeds incoherently throughout the excited-state decay.

**Fig. 3:**
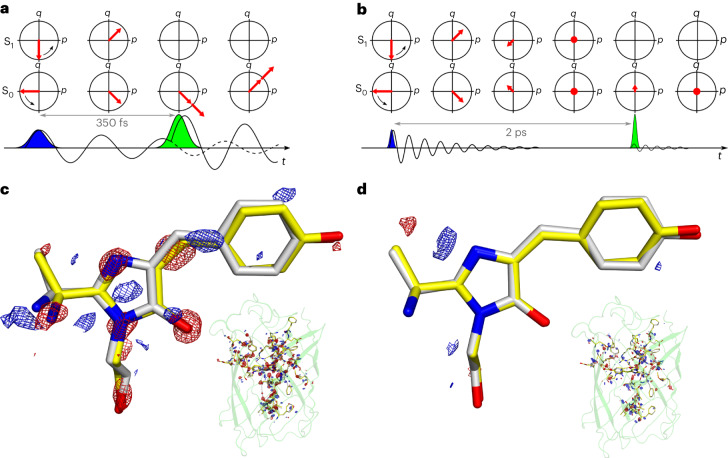
Amplification of structural motion requires the pump–dump delay to be within the vibrational dephasing time, not the excited-state lifetime. **a**,**b**, Vibrational-coherence transfer dominates the observed displacements on a femtosecond timescale for the PDP condition (**a**). A test for Tannor–Rice coherent dynamics moved the dump delay to 2 ps (**b**), after vibrational dephasing but well within the 50-ps excited-state lifetime. **c**,**d**, A comparison of the PDP experiment with a 350-fs dump time (**c**) conducted at SACLA reproduces the LCLS experiment in Fig. 2 in detail. *F*_o_–*F*_o_ difference maps are shown at 3*σ* level and 1.5 Å resolution. Strong decay of the difference signals is observed when dumping at 2 ps after dephasing, as predicted by Tannor–Rice coherent control (**b**,**d**). A schematic representation of coherence in the ground state (S_0_) and excited state (S_1_) is shown in the Wigner phase-space representation as the evolution of momentum *p* and position *q* of the S_0_ and S_1_ wave packets (**a**,**b**). A full coherence simulation using a density-matrix calculation including Wigner transforms is presented in Fig. 4, Extended Data Fig. 8 and Supplementary Section 13.

In separate experiments at SACLA, a series of tests were performed to confirm the assignment of the ultrafast structural changes to ground-state coherent motions (Fig. 3 and Supplementary Section 5.3). First, for PP conditions, we followed the decay of the photoinduced signals by adjusting the pump–probe delay (Supplementary Fig. 38). A 3-ps pump–probe delay significantly decreased the photoinduced differences compared to the 1-ps delay. The 1-ps delay for the PP and PDP conditions reproduced the results from LCLS (Supplementary Fig. 40).

Second, for PDP conditions, we tested the dump-induced amplification of ground-state motion as a function of vibrational-coherence decay. Data at 3 ps and 100 ps for the PDP condition at ~350-fs dump time confirmed the overall decay of the initial femtosecond motions (Supplementary Fig. 41). The decay is comparable to the ~4.5-ps lifetime of the induced absorption at 440 nm associated with the ground-state intermediate (Extended Data Fig. 3e,f).

**Fig. 4:**
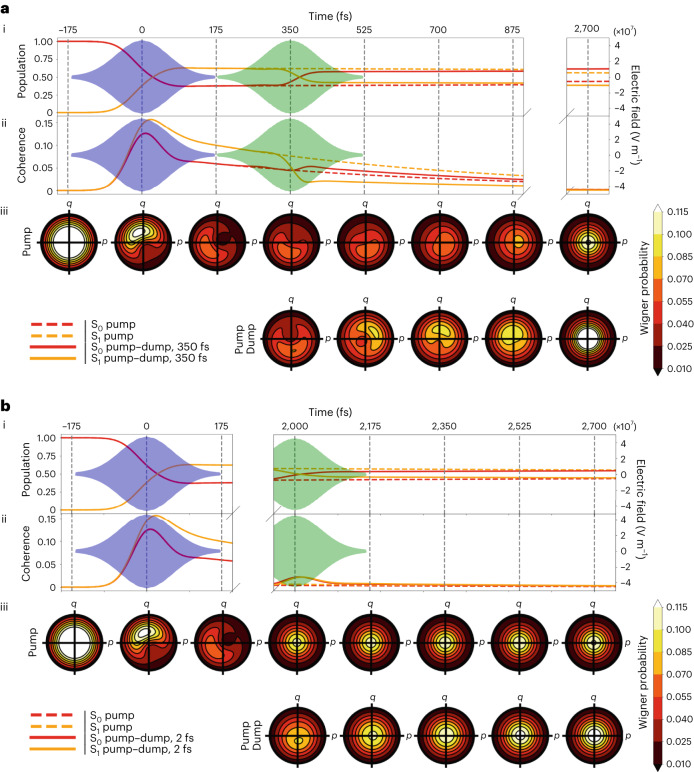
Density matrix calculations and resulting Wigner phase-space probability distributions for TR-SFX experimental conditions on rsKiiro using different pulse schemes. Calculations were performed using the parameters listed in Supplementary Table 11, which are representative of the TR-SFX conditions and use the methodology described in Supplementary Section 13. **a**,**b**, Comparison of the populations (**a**(i) and **b**(i)) and coherences (**a**(ii) and **b**(ii)) of the S_0_ (red) and S_1_ (yellow) electronic states over time, with the pump (dashed lines) and pump–dump (solid lines) schemes shown, for 350-fs pump–dump delay (**a**, corresponding to Figs. 2 and 3a) and 2-ps delay (**b**, corresponding to Fig. 3b). A coherence comparison is made between the pump and short 350-fs (**a**) and long 2-ps (**b**) pump–dump delays. The Wigner phase-space distributions of the S_0_ ground state for all pulse schemes are shown in **a**(iii) and **b**(iii), with **a**(iii) showing an increase in asymmetry due to Tannor–Rice coherence transfer from excited to ground state after dump interaction within vibrational dephasing. In contrast, as expected, the longer 2-ps delay after vibrational dephasing in **b**(iii) shows minimal impact on the distribution, with a small generation of position (*q*) and no impulse momentum (*p*) transferred, but with population transfer after the dump interaction. The corresponding Wigner transformations for S_1_ are shown in Supplementary Fig. 58 using surface representations.

Third, we proceeded with a test of vibrational wave-packet assignment based on coherent control methodology (Fig. 3). In the wave-packet picture, the nuclear motion is also expected to continue after electronic structure change in the Born–Oppenheimer approximation (Fig. 2b). Specifically, the impulse momentum of the wave packet present in the S_1_ excited state may be transferred to the electronic ground state if the stimulated emission interaction occurs within the vibrational dephasing time, as shown by the Tannor–Rice scheme^27,31–33^. This coherence transfer adds to nuclear coherence that is created by the displacement, such that both impulse momentum *p* and position *q* are generated in the ground state after the dump interaction. This coherence furthermore adds to the ground-state coherence generated by the first pump pulse (Figs. 2–4). The initial excited-state coherence created by the pump pulse results only from displacement and is not impulsive, because the ground state is fully dephased before the arrival of the first pulse. Based on impulsive stimulated Raman spectroscopy measurements of vibrational dephasing of the neutral *cis* chromophore of green fluorescent protein (GFP^34–36^; which shows a typical ~1-ps time constant of decay^34–36^), we chose a 2-ps dump delay relative to the pump to take measurements that are representative of completed vibrational relaxation, but are still well within the 50-ps electronic population-decay time. Following the wave-packet assignment, we predict that the amplitude of momentum transfer by the dump pulse should decay in the delayed condition (2 ps; Fig. 3b). The comparison between the 350-fs and 2-ps dump times very clearly shows a full decay of the photoinduced differences, in line with the predictions from optimal coherent control theory (Fig. 3). This provides further evidence for the conclusion that the photoinduced displacements observed in the TR-SFX experiments are due to impulsive, coherent wave-packet motions on the electronic ground-state potential-energy surface rather than in the electronic excited state. The results of the tests that were performed thus confirm that the PP differences can also be confidently assigned to coherent wave-packet motion in the electronic ground state.

The creation and evolution of the impulse momentum *p* and position *q* of nuclear coherence in these experiments are depicted as wave packets in Fig. 2 and in the phase space in Figs. 3 and 4. The first pump interaction creates impulse momentum in the ground state in the direction of the nuclear binding force, and it creates position *q* in S_1_ that is not impulsive^23–26^. Evolution of the coherences in S_0_ and S_1_ is depicted with the periodic interconversions of *p* and *q* according to their frequencies (Figs. 2–4) and their amplitude decays with the vibrational dephasing time. With the arrival of the dump pulse at 350 fs within the dephasing time, the wave-packet momentum is transferred from S_1_ to S_0_ and creates additional position *q* via the displacement (Figs. 2–4). When the dump pulse arrives at 2 ps, the vibrational coherence has decayed but the electronic excitation has not. In this case the dump pulse does not transfer momentum but only creates position (Figs. 3b and 4b).

We performed a non-perturbative density-matrix simulation that fully verified the coherence dynamics that were observed (Supplementary Table 11 and Supplementary Section 13). The electronic and nuclear wavefunctions are represented by the density matrix, and the pump and dump interactions are included according to the experimental frequencies and timings. The density matrix evolves under the Liouville–von Neumann equation, and the coherence and population progression and transfer are analysed using Wigner transforms. These can be separated for S_0_ and S_1_ to provide their momentum and position^37^. Specifically, the wave-packet dynamics are observed in the transfer and subsequent rotation of the two-dimensional surfaces in phase space that represent the quasi-probabilistic distributions of position and momentum, which are equivalent to the schematic depiction in phase space shown at the top of Fig. 3. The rotation in phase space interconverts the impulse momentum and position and represents the vibrational coherence. Vibrational dephasing returns this surface to the centre of phase space, which represents the dephased populations in S_0_ and S_1_. The simulations are shown for the ground state in Fig. 4, and demonstrate the expected coherence evolution and transfer for the PP and PDP conditions as well as the dephasing conditions, with loss of momentum transfer that matches the diagrammatic representation shown in Fig. 3. Strikingly, the density-matrix simulation for the PDP condition doubles the magnitude of the ground-state coherence following the dump interaction (Fig. 4). This demonstrates the essential mechanism of amplification of ultrafast motion under conditions of PDP within the vibrational dephasing time.

The simulations lend strong support to the assignments for the PP and PDP crystallographic data, with the latter showing distinct amplification of motion. The wave-packet generation in these simulations can be readily extended to a double-well adiabatic potential that describes the time-resolved crystal structures (Fig. 2). The phase-space representation is particularly appropriate for representing and analysing the quantum dynamics, because it relates more directly to the real-space measurements of TR-SFX. The conclusions are furthermore fully consistent with the wave-packet description in Raman spectroscopy^23–25,31–33,38^ and the wave-packet description of coherent control by Tannor et al.^31^, Kosloff et al.^32^, and Ruhman and Kosloff^33^.

**Fig. 5:**
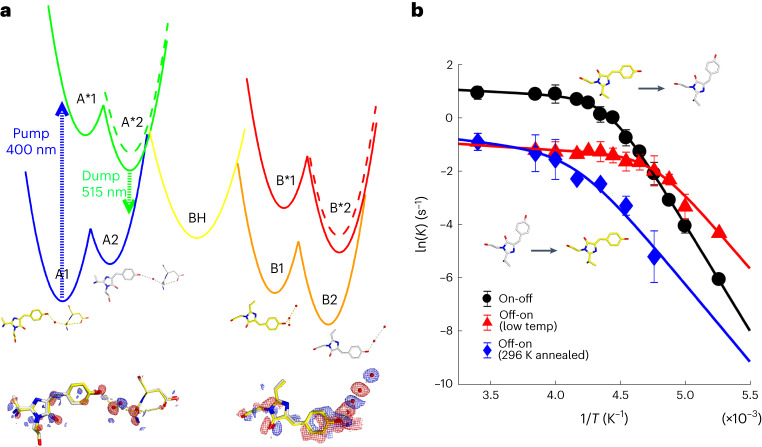
Temperature dependence of *trans*(A)–*cis*(B) and *cis*(B)–*trans*(A) photoisomerization kinetics. **a**, Potentials are shown as adiabatic states that contain the A1 (PDB 7QLM and 7QLJ) and A2 (PDB 7QLN and 7QLO) off-state and B1 (PDB 7QLK) and B2 (PDB 7QLL and 7QLI) on-state structures as shown. The protonated anionic BH state is a putative intermediate that connects the photocycle (Fig. 1). Cryo-trapping of the unrelaxed on state resolved the double-well structural features of the B1 and B2 on states (Supplementary Sections 9 and 11 provide crystallographic details and full thermodynamic modelling, respectively). **b**, Arrhenius plots for the on→off (black) and off→on (red, with low-temperature on→off pre-conversion; blue, with on→off pre-conversion at 296 K) kinetics under continuous illumination at 473 nm and 405 nm, respectively, showing convex behaviour in both directions. The off→on kinetics that included high-temperature annealing before conversion (blue) showed significantly reduced kinetics and shifting of the transition temperature to higher values. Both high- and low-temperature regions involved photoisomerization in both on→off and off→on directions, as shown from X-ray-crystal structural analysis (Supplementary Section 9). The error bars in **b** are the standard error.

To derive the functional importance of the double-well adiabatic potential discovered in our ultrafast crystallography experiments, we combined the structural information with kinetics measurements and thermodynamics modelling. The double-well adiabatic nature of both the off and on states was additionally shown from the thermodynamic analysis of reversible photoisomerization as well as the excited-state decay. The temperature dependence of both *trans*–*cis* and *cis*–*trans* photoisomerization shows convex non-Arrhenius kinetics in which the transition temperature is additionally sensitive to structural annealing, which lowers the ‘A*2’ potential level (Fig. 5). Convex Arrhenius kinetics are conventionally analysed using parameters for double-well potentials. Modelling of the non-Arrhenius kinetics invoked an entropy–enthalpy compensation scheme and retrieved the relaxation parameters of the second energy level of the double-well potential (Figs. 2 and 5, A1*, A2*, Extended Data Fig. 1e,k and Supplementary Section 10.5). The detailed modelling uses the experimental separation of the radiative and non-radiative transitions, which show different barrier values (Extended Data Fig. 1f,l). Full thermodynamics treatment and theoretical considerations are provided in Supplementary Section 10. The reaction model that emerges is fully consistent with the time-resolved crystallography and also the QM/MM calculations. The barrier for internal conversion arises from viscosity dependence, which indicates that substantial reconfiguration is involved. Experimental evidence from the PDP yield in crystals and solutions furthermore supports that the non-adiabatic reaction dynamics proceed thermally throughout the 50-ps lifetime of the off state (Extended Data Fig. 2). The photoisomerization trajectory is therefore not directly observed in the time-resolved measurements because the photoproduct accumulates throughout the duration of the excited-state lifetime of 50 ps.

In conclusion, we have demonstrated a successful coherent control experiment with X-ray crystallographic observation. We show that ultrafast structural changes observed in TR-SFX of a reversibly photoswitchable fluorescent protein can be assigned with the application of optical control. The evidence illustrates that the data from conventional femtosecond PP measurements of the structural dynamics of the chromophore and protein are unrelated to the photoisomerization coordinate in our experiments. We present extensive experimental evidence that the PP and PDP data collected within the vibrational dephasing time in effect measured impulsively prepared electronic ground-state coherent wave-packet motion. The results are relevant to typical experimental conditions for TR-SFX that use a 50–100-fs optical pulse duration that limit the frequency of impulsively driven modes. Because the crystallographic differences are dominated by modes that have large displacement, increasing the experimental bandwidth will probably still contain such low-frequency modes^27^. Furthermore, the results clearly show that the commonly used technique of rate kinetics analysis, which is restricted to model concentration differences of static species, does not apply to the assignment of structural dynamics on the ultrafast timescale of vibrational dephasing that additionally have heterogeneous contributions. This report offers fundamental tools and methods for experimental execution and theoretical analysis of ultrafast crystallography. We emphasize the use of phase-space representation of the density matrix using the Wigner transform to visualize the coherent motions that are seen. The Wigner transform provides the evolution of nuclear position from explicit quantum dynamics simulations that use experimental conditions including coherent control and electronic and vibrational coherence. These methods are generally applicable, and their purpose is fully demonstrated with this example of ultrafast structural dynamics of a reversibly photoswitching fluorescent protein.

## Methods

### Protein expression and purification

Recombinant expression of the rsKiiro sequence cloned into pEGFP-N1 (Clontech), containing L62A and M159T mutations of the monomeric reversibly switchable mEos3 (ref. ^39^) construct based on the EosFP fluorescent protein from the stony coral *Lobophyllia hemprichii*^28^, was carried out in *Escherichia coli* BL21(DE3). Large-scale fermentation was carried out using a 50-l culture of Terrific broth (48.2 g l^−1^) (Sigma-Aldrich, T9179) containing 50 mg l^−1^ kanamycin and 10.1 g l^−1^ glycerol at 37 °C. When the culture reached an optical density at 600 nm (OD_600_) of ~2, the bioreactor was cooled to 15 °C, and induction of expression was carried out by the addition of isopropyl β-d-1-thiogalactopyranoside (0.12 g l^−1^; Sigma-Aldrich, CAS 367-93-1). Lysis was carried out using a buffer (50 mM Tris-HCl pH 8.0, 100 mM NaCl) per gram of wet cell mass including 0.1 mg ml^−1^ DNase I (Sigma-Aldrich, CAS 9003-98-9) and one tablet of EDTA-free protease inhibitors (Sigma-Aldrich) per 20 g of cell mass. The protein was purified using His-Pur Ni-NTA chromatography resin (Thermo Fisher Scientific) by elution in a single isocratic step with elution buffer (50 mM Tris-HCl, pH 8.0, 100 mM NaCl, 350 mM imidazole). The eluate was concentrated and exchanged into gel filtration buffer (50 mM Tris-HCl pH 8.0, 100 mM NaCl) and purified using an XK26/70 chromatography column (GE Lifesciences) packed with 300 ml of Superdex S75 gel filtration resin (GE Lifesciences). Fractions were concentrated to ~60 mg ml^−1^ and exchanged in final buffer (50 mM Tris-HCl pH 8.0, 50 mM NaCl) before storing at −20 °C.

### Crystallization and sample preparation

Crystallization conditions were based on those previously described^38^. Micro-crystals were grown using the seeded batch method. Two different conditions were used—10 mg ml^−1^ protein concentration and 25% (wt/vol) PEG 3350 (Sigma-Aldrich, CAS 25322-68-3) and 15 mg ml^−1^ and 30% (wt/vol) PEG 3350—and both sets of conditions included 0.1 M Tris (Sigma-Aldrich, CAS 77-86-1) pH 8.5, 0.2 M lithium sulfate (Sigma-Aldrich, CAS 10377-48-7) and 1.5% (vol/vol) seed. Seed stocks were prepared using either condition and subsequently filtered with 50- and 30-μm CellTrics filters (Sysmex Partec). Crystallization was performed in 2-ml round-bottom Eppendorf tubes, and the addition of reagents followed the order ‘buffer, precipitant, protein and seed’ in a final 1-ml volume followed by gentle mixing. Needle-shaped micro-crystals with dimensions of ~3 × 5 × 10–100 μm matured after 24 h at 20 °C. To reduce issues with blockages during crystal injection, especially with the Gas Dynamic Virtual Nozzle (GDNV) during LCLS^40^ injection, further size optimization was performed by breaking up the micro-crystals using glass beads (Supplementary Section 1 describes the procedure).

### SFX sample injection

At LCLS CXI, micro-crystals were delivered into the interaction region using a GDVN^40^ with 75-μm aperture, which was modified by removing the opaque coating surrounding the inner capillary to allow pre-illumination of the sample before injection. The flow rate was varied between 10 and 30 μl min^−1^ to maximize the hit rate and stability. At SACLA, a piezo-driven droplet-on-demand injector with an 80-μm nozzle (MICROJET, IJHDS-1000) was used. Approximately 300 μl of crystal slurry at 1 × 10^8^ Xtal ml^−1^ was reverse-loaded through the jetting aperture to reduce the chance of blockages and crystal settling in the lines and reservoir. The piezo was driven with 110-V, 100-μs current pulses, which were slightly varied depending on the jetting behaviour. With optimization of the driving current pulse, jet position, jet timing and sample concentration, a hit rate of 50–80% was maintained for the majority of the data collection. This was greatly assisted by the synchronized live imaging of the droplets and almost live (few second delay) hit rate provided by the SACLA pipeline^41^. At PAL-XFEL, microcrystal slurry was resuspended in monoolein (1-oleoyl-*rac*-glycerol, Sigma-Aldrich) in a 1:2 ratio of slurry (6.4 × 10^7^ Xtal ml^−1^) to monoolein, chosen for stable jetting and hit rate. The monoolein suspension was injected into the interaction region using a modified 100-μl syringe (Hamliton Gastight 1710 with 22-G style 3 needle), whose plunger was coupled to a second larger-diameter (5 mm) water syringe driven by a high-performance liquid chromatography pump. The water syringe was driven at 75.3 μl min^−1^, corresponding to 6.42 μl min^−1^ in the Hamilton syringe, which in turn corresponds to an extrusion velocity of ~6 mm s^−1^ or ~200 μm per pulse at 30 Hz out of the 150-μm needle aperture. This high flow rate was necessary to ensure sufficient sample exchange between laser shots to prevent double exposure of sample due to the pump laser spot size of ~140 μm (FWHM) (see below) and possible ‘light piping’ up the continuous monoolein stream.

### SFX data collection

This work includes data from four XFEL beamtimes, LCLS CXI LR23 (February 2018), SACLA BL3 EH2 2017B8008 (March 2018), 2019B8021 (November 2019) and PAL-XFEL 2020-2nd-NCI-007 (September 2020). A summary of the conditions and delays collected at each beamtime is provided in Extended Data Table 1.

At CXI, X-ray diffraction data were collected using the Cornell Stanford Pixel Area Detector (CS-PAD)^42^ at a distance of 50.7 mm, determined through optimization of unit-cell distributions (Supplementary Figs. 1 and 2). LCLS was operated at 9.5 keV with ~1 mJ of X-ray energy. At SACLA, the MPCCD^43^ detector was used, and the increased quantum efficiency of the phase III version of this detector allowed the SACLA to be operated at 10.5 keV, increasing the measurable resolution without significant reduction in signal to noise. The detector distance was determined to be 50.6 mm (Supplementary Figs. 3 and 4). Data were collected, interleaved pumped and unpumped with a ratio of 5:1, throughout the SACLA and LCLS beamtimes, and a comparison of ‘only pre-illuminated’ (that is, no femtosecond illumination) and interleaved unpumped datasets showed no significant differences, demonstrating that scatter from the pump or dump pulses did not impact subsequent shots. This allowed all dark data to be merged for creating difference maps. PP and PDP TR-SFX data were collected at picosecond and sub-picosecond delays, as well as dump only and negative delay controls. At PAL-XFEL, the MICOSS^44^ sample chamber was used with a Rayonix MX225-HS detector in 4 × 4 binning mode to allow 30-Hz operation. The XFEL was operated at 12.4 keV with a bandwidth of 17.5 eV (r.m.s.) and pulse duration of 21.47 fs (FWHM), and the beam was attenuated to ~0.6 mJ as a compromise between X-ray flux and stable jetting. Due to the slower jetting speed of the viscous media injector, the interleaving pattern was set at 1:1 to more accurately characterize the pump scatter onto subsequent crystals.

### TR-SFX optical excitation

At LCLS LR23, optical excitation pulses were provided by the on-site synchronized Ti:sapphire laser system. The 515-nm dump pulses were produced using an optical parametric amplifier (OPA) (TOPAS-Prime, Spectra Physics), and the remaining fundamental was taken after the final stage of the OPA and doubled using a 100-μm second-harmonic generation (SHG) beta-barium borate (BBO) nonlinear crystal (EKSMA Optics) to create the 400-nm pump pulses. Temporal delay between the two pulses was controlled using a motorized mirror inside the OPA. The pump and dump optical arms each had their own linear polarizer and *λ*/2 waveplate to allow independent control of polarization and pulse energy. The ‘needle-like’ nature of the crystals causes a tendency to align vertically out of the GDVN jet, and to reduce potential pumping suppression due to electric-field decomposition inside the crystals^45^, both the pump- and dump-pulse polarizations were inclined at the same 45° to the jetting direction. Spatial overlap between the pulses and the X-rays was achieved using the fluorescence on a Ce:YAG scintillator screen, and this overlap was checked at the start and end of every shift. The final energy densities on the target of the pump and dump were 2 ± 0.1 mJ mm^−2^ and 4 ± 0.1 mJ mm^−2^, respectively. Samples were pre-illuminated using an array of five 10-W, 490-nm LEDs on a 1.5-m loop of tubing before injection. In addition, light from a single 3-W, 490-nm LED, installed outside the experimental chamber, was focused onto the transparent tip of the GDVN using a series of lenses. Course temporal overlap between the pump and dump pulses was obtained using a fast photodiode and oscilloscope. The final timing between the pump and dump pulses (~350 fs) and time zero with the XFEL was achieved by performing cross-correlations in a scintillator screen (Supplementary Fig. 6).

During SACLA 2017B8008 and 2019B8021, the optical pulses were provided by an onsite synchronized Ti:sapphire laser. The 515-nm pulses were generated by an OPA (HE-TOPAS), and for the 400-nm pulses a proportion of the fundamental was taken before the OPA and sent on a long optical delay to compensate the path inside the OPA, and then frequency-doubled using a 100-μm SHG BBO crystal. The pump–dump temporal delay was controlled by a motorized delay stage installed in the 515-nm arm. The samples were focused on the X-ray interaction region using a 300-mm lens, achieving the same energy densities as those used at LCLS. Pre-illumination was performed by illuminating the glass tip of the droplet injector with the unfocused beam (5 × 3 mm) of a 60-mW 488-nm c.w. diode laser. Both the power density and the pump–dump delay of ~500 fs, which is well within vibrational dephasing, matched the previous LCLS conditions, but frequency-resolved optical gating (FROG) characterization showed the pulse durations to be significantly shorter at 40 fs and 52 fs for the pump and dump, respectively (Supplementary Table 1).

### Data processing

The rsKiiro crystals were indexed in space group *P*2_1_2_1_2_1_, with *a* = 39.44 ± 0.05, *b* = 74.18 ± 0.12, *c* = 78.83 ± 0.12, *α* = 90°, *β* = 90° and *γ* = 90°. Analysis of TR-SFX data at LCLS LR23 was performed using the Cheetah^46^ and CrystFEL^47^ software packages. Dark scans were taken approximately every 20–40 runs, which recorded images from the CS-PAD detectors in the absence of illumination to establish bad/damaged pixels, which were subsequently masked out before running indexing algorithms. The Cheetah package was used to perform hit finding. Before indexing and integration, nearly every run was manually masked to remove substantial shrouding caused by the shadow of the nozzle on the detected on images. The CrystFEL package was used to index and integrate the hits identified by Cheetah. Two indexing algorithms, DIRAX^48^ and MOSFLM^49^, were compared to identify which performed better, with both being fed the previously determined unit cell. The beam centre was monitored to account for any drift in the beam position relative to the input detector geometry file, with any offset being corrected. The detector distance was also refined in steps of 0.1, 0.05 and finally 0.01 and 0.001 mm by monitoring the detected distribution of unit cells from the output of the indexing algorithm until it narrowed and became symmetric (Supplementary Figs. 1 and 2). The optimized geometry yielded single narrow distributions with standard deviations of <0.5 Å for lattice axes and ~1° for the angles, while the unoptimized geometry showed deviations >1 Å in addition to showing a bimodal distribution for the *c* axis of the lattice. An L-test was also used in conjunction to monitor for crystal twinning, which appeared to be very high until the proper detector distance was determined. With unoptimized geometry, a twin fraction of 16–17% was observed, which improved to 6% upon optimization. This value remained slightly higher than expected, but was acceptable given a single set of narrow lattice distributions and appropriate *R*-factors. Although the detector was moved and replaced on a number of occasions, it was not found to be necessary to optimize the detector distance for each run individually, as a single optimized distance produced reasonable cell distributions and indexing results for the entire dataset. Indexing results were also found to be relatively insensitive to algorithm parameters such as integration radii or minimum signal-to-noise ratio requirements. For a number of runs, an electronic gain mask was used to avoid saturation of pixels at low resolution while maintaining sensitivity at high resolution. After indexing all runs with the optimized detector geometry, masked images and gain mask applied, the individual intensities were merged using the Monte Carlo method from the routine process_hkl included in CrystFEL, without consideration of a partiality model.

Data from SACLA 2019B were analysed in a manner similar to the data from LCLS with a number of minor differences. First, the nozzle in the SACLA experiment was sufficiently far away to avoid the shrouding issues from LCLS, and so no additional masking was needed beyond the removal of damaged pixels. Second, all SACLA data were collected in a single-gain mode, so no gain mask was included for the analysis. Finally, two additional indexing algorithms were tested for the analysis, TakeTwo^50^ and XGANDALF^51^. The SACLA experiment had a particularly high hit rate (>60%), which typically means that recorded images contain diffraction patterns from multiple micro-crystals. These two algorithms were designed specifically for SFX experiments and a view to the indexing of multiple lattices per single image. For our dataset we found that XGANDALF performed the best, with an indexing rate of over 100% (that is, more than one lattice per image) for most runs. The same sensitivity to detector distance and systematic optimization was required (Supplementary Figs. 3 and 4).

At PAL-XFEL, Cheetah was only used for peak finding and for applying the beam-stop mask, not live hit-rate monitoring. Hit-finding parameters were initially optimized based on CrystFEL’s indexing performance and manual inspection of laser off-condition images. The PAL-XFEL experiment used a commercial Rayonix MX225-HS detector. Unlike the detectors at SACLA and LCLS, this is a commercial detector, which has a pre-applied geometry correction and automatically subtracts a dark image, negating the need for manual hot/dark pixels masking in Cheetah. This meant that only the beam-centre and detector distance had to be optimized, not the geometry of individual detector panels. To maximize the merged data quality, two indexing programs, MOSFLM^49^ and XGANDALF^51^, were directly compared. XGANDALF was found to provide the best results, so it was selected as the indexing algorithm.

### Crystallographic analysis

Bash scripts based on ref. ^52^ processed each light and dark bin to generate so-called *Q*-weighted electron difference density maps (maps based on Bayesian statistical analysis by Ursby and Bourgeois^53^, and Terwilliger and Berendzen^54^), which were then interpreted by generating extrapolated electron density, for which ‘light’ coordinates were refined too. The PHENIX reflection conversion function, using the massage option, was used to convert intensities to structure factors^55^. Calculated structure factors from SFALL (CCP4i^56^) using the ground-state refinement model were combined with observed structure factors and combined into one file using CAD (CCP4i^56^). The observed structure factors were then scaled onto an absolute scale using anisotropic scaling in SCALEIT (CCP4i^56^), using a resolution cutoff at 1.5 Å for all experimental conditions. For coordinate refinement, relevant low- and high-resolution limits were applied depending on the experiment (maximum of 1.35, 1.44 and 1.42 Å at LCLS, SACLA and PAL-XFEL, respectively). The low-resolution limit was set between 20 and 30 Å. Difference structure factors were then weighted as described by Pandey et al.^52^, and fast Fourier transform (CCP4i^56^) was used to convert the structure factors into a real-space map.

Extrapolated electron-density maps were generated, and extrapolated coordinates then refined to these maps. Difference structure factors, Δ*F*, were added in a linear combination in multiples of *N*_EXT_ to the calculated dark structure *F*_Dark_ to generate extrapolated structure factors, *F*_EXT_. The negative electron density above the 3 r.m.s. level and a 7-Å radius around the chromophore were then integrated using published Fortran code^52^. The point where negative density started to build up was used to determine a characteristic *N*_EXT_ value, which was then used to generate the final extrapolated structure factors. Coordinates were refined against the extrapolated structure factors using five cycles of rigid body refinement in REFMAC^57^ and further real-space refinement using Coot^58^ real-space refinement. The population transfer (PT) can be approximated from the *N*_EXT_ value as PT = 200/*N*_EXT_.

Crystallographic statistics are shown in Table 1 and Extended Data Table 2 for LCLS, Supplementary Tables 4 and 5 for SACLA and Supplementary Table 6 for PAL-XFEL.

### Solution steady-state spectroscopy

The highly fluorescent ‘on’ state of rsKiiro, corresponding to the *cis* conformation of the chromophore, has a characteristic absorption peak at 486 nm with a calculated absorption coefficient of 117,500 M^−1^ cm^−1^. When illuminated within this band, it photoswitches to a metastable ‘off’ state with the *trans* conformation, with peak absorption at 388 nm (44,000 M^−1^ cm^−1^) and highly reduced fluorescence (Extended Data Fig. 1b). The UV–vis solution spectra of the on and off states, collected with a spectrophotometer (Agilent 8453), are provided in Extended Data Fig. 1a. Extinction coefficients were calculated from the amino-acid aromatic ring absorption at 280 nm relative to the parent molecule (Skylan-NS^59^). Once converted, the off state thermally recovers to the on state with a time constant of ~40 min at 293 K. The thermal recovery rates were measured as a function of temperature (284–323 K) using a water feed cuvette holder coupled to a water bath (Julabo F12). Excited-state barrier heights (*E*_*α*_) were determined by fitting of the Arrhenius equation:

$${\frac{1}{\tau }={{\mathscr{A}}}_{1}+{{\mathscr{A}}}_{2}{\rm{e}}^{-\frac{{E}_{\alpha }}{{RT}}}}$$

where ${{\mathscr{A}}}_{1}$ and ${{\mathscr{A}}}_{2}$ are temperature-independent constants, *R* is the gas constant, *T* is the temperature and *τ* is the inverse rate constant. A plot and fitting are shown in Extended Data Fig. 1c, where the fitting yields an energy barrier between the off and on states of 91 ± 5 kJ mol^−1^_._

### Microsecond TA spectroscopy

TA spectroscopy was performed on solution samples of rsKiiro using a home-built device^60^. A 6-μl volume of 6 mg ml^−1^ rsKiiro solution was added to 50 μl of buffer to give an OD_400_ of ~0.2 with the 3-mm path length of the three-windowed quartz cuvette (Quartz SUPRASIL High Precision Cell, Hellma Analytics). The sample was pre-illuminated using an unfocused 15-mW, 473-nm, c.w. Transistor-transistor logic trigger laser for 10 ms at 20 Hz. A xenon pump lamp was filtered using a blue filter (FGB25 Thorlabs), resulting in ~150 μJ per flash in the 300–450 nm range, focused to a 3 × 1-mm elliptical spot inside the sample cuvette. The thermal recovery time constant of rsKiiro is ~40 min at 20 °C, so the sample was left with all three light sources flashing for 1 h to equilibrate before data collection. Approximately 4,000 PP spectra were collected at each delay. The time-resolved spectra are provided in Extended Data Fig. 3i. There is an increasing absorption at 486 nm with a time constant of 40 ± 10 µs, corresponding to formation of the *cis* anion (Extended Data Fig. 1a).

### Femtosecond optical TA spectroscopy

Femtosecond TA spectroscopy of rsKiiro was performed using a previously described system^61^. The 400-nm pump pulses were generated by doubling the 100-fs fundamental at 800 nm (Hurricane, Spectra Physics) in a 100-μm-thick SHG-BBO device (EKSMA optics), and 515-nm dump pulses were generated using an OPA (OPA-C Spectra Physics). White-light probe pulses were generated using filamentation in a CaF_2_ glass window.

A 20-μl solution sample of rsKiiro at 62 mg ml^−1^ was loading into a liquid cell (Harrick Scientific Products) between 1-mm (front) and 2-mm (back) sapphire windows (Crystran), using a 25.6-μm spacer corresponding to a peak absorption of ~0.3 at 380 nm when converted to the off state. The sample was continuously translated and illuminated using an unfocused 50-mW, 488-nm diode laser to convert the sample to the target off state. A second-order polynomial and linear interpolation was used to correct the majority inherent spectral chirp of the white-light probe. The Ultrafast Spectroscopy Modelling Toolbox^62^ was used to globally fit the corrected TA datasets and recover the time-independent evolution associated difference spectra (EADS).

The weak pumping (7 GW cm^−2^) PP transient absorption difference spectra of the off state with 400-nm pumping are shown in Extended Data Fig. 3d. Two sequential spectra with similar forms were recovered, with time constants of 7.6 ps and 50 ps. The negative signal observed at 385–400 nm is assigned to the ground-state bleach (GSB), the positive signal between 420 and 475 nm to excited-state absorption (ESA) and the negative signal at >480-nm to stimulated emission (SE). It is of particular note that there is no signal corresponding to the formation of the ground *cis* state at 490 nm within the maximum delay of the system (~2.5 ns). Temperature-controlled PP TA spectroscopy was performed using a home-built temperature-controlled liquid cell with a range of 10–50 °C and accuracy of 1 °C.

The addition of a 515-nm dump pulse after the 400-nm pump pulses causes substantial modification of the spectra. Comparison of temporal lineouts (Extended Data Fig. 3f) for the different regions for the first 50 ps and globally fitted spectra recovered after the dump pulse (Extended Data Fig. 3e) shows the remaining signal in the GSB and ESA exhibits a reduction in decay constant from ~8 ps to ~4.5 ps.

To analyse the effect of different short pump–dump delays, the PP delay was fixed to 1.5 ps and the pump–dump delay varied (Extended Data Fig. 3g). It can be seen that, for negative pump–dump delay, the TA signal is the same as that of the regular PP spectra, and the pump–dump delays of 150 fs, 275 fs and 400 fs are almost indistinguishable in the GSB and ESA signals.

To confirm that 515 nm is a suitable dumping wavelength and rule out the possibility of off-resonant pumping, PP TA measurements were performed using 515 nm as the pump. Extended Data Fig. 3h shows the dispersion-corrected lineouts for several pump–probe delays at two different energy densities, and it can be seen that a 0.007 mJ mm^−2^ 515-nm pulse (that is, sufficient to induce >0.1 OD TA differences in the on state) produces no measurable signals within the 0.1-mOD sensitivity of the instrument. A further order-of-magnitude increase in the energy density of the 515 nm pulse still did not produce any measurable signals.

### Femtosecond infrared TA spectroscopy

Due to a weak optical signature associated with isomerization in the visible TA, mid-infrared femtosecond TA spectroscopy measurements were carried out on rsKiiro to look for vibrionic signatures. A 95 mg ml^−1^ rsKiiro solution in low-salt buffer (50 mM Tris-HCl, 100 mM NaCl at pH 8) was loaded into a 1-inch-diameter liquid cell, with two CaF_2_ windows and 6-μm spacer giving a final OD of ~0.7 at 490 nm. Deuterated samples were made using the same buffers but with deuterated water at pD 8.4, and the normal protein solution was diluted at 500 μl of protein to 2 ml of deuterated buffer, reconcentrated and loaded into the liquid cell, giving a final OD_490_ of ~0.8.

To inform the ultrafast infrared measurements and assignments, steady-state Fourier-transform infrared (FTIR) spectra were collected using a Bruker ISF 66/S spectrometer. The samples were illuminated with a 100-mW 488-nm diode laser to collect both the on and off states. Comparison of the deuterated and non-deuterated spectra, as well as those of from Dronpa and GFP, informed the peak assignments shown in Extended Data Fig. 4c. The steady-state difference spectra of deuterated rsKiiro for off minus on states are shown in Extended Data Fig. 4a.

Femtosecond infrared TA difference spectra were collected over a temporal range of −40 ps to 2 ns between 1,800 and 1,500 cm^−1^ using a previously described system^63^. Samples were pre-illuminated with a 488-nm diode laser and optically pumped using 100-fs, 400-nm pulses at an energy density of 0.018 mJ mm^−2^. The PP dataset was globally fitted with a sequential model to produce EADS with time constants of 5.79 ps, 60.65 ps and a long-lived component (Extended Data Fig. 4b).

In the fast, 5.78-ps EADS spectra, we observe GSBs corresponding to the *trans* neutral at 1,689, 1,644 and 1,513 cm^−1^, assigned to the C=O, C=C and phenol-3 bands. With induced absorption at 1,668 and 1,596 cm^−1^, these are assigned to the rearrangement of the Arg66 asymmetric and symmetric CN_3_H_5_^+^ modes, respectively, suggesting rearrangement of the Arg66 residue/hydrogen-bonding network—the first step in the photoreaction. It is also possible that the 1,668 cm^−1^ feature contains contributions from the C=O mode, as was seen in Dronpa^63^. The 60-ps EADS shows a new ESA at 1,632 cm^−1^, corresponding to *ν*(C=C), which is indicative of a change in the methyl bridge dihedral angle; this is further supported by a new GSB at 1,618 cm^−1^ over the phenol-1 stretch of the *trans*-neutral chromophore. This combination leads us to conclude that isomerization must occur within the 50/60-ps time constant. However, the C=C peak does not correspond to the final position of the steady *cis* state (1,626 cm^−1^), indicating an intermediate conformation relative to the final product. We note that cryo-trapping and macromolecular crystallography resolved a minor second chromophore conformation (Supplementary Fig. 50). The absorption strengths of the phenol-1 (1,576 cm^−1^) and phenol-3 (1,497 cm^−1^) peaks are linked to the protonation state, strongly absorbing in the anion but only weakly in the neutral. The complete absence of these peaks in all TA spectra (except 1 ms) supports the findings of the microsecond TA spectra (Extended Data Fig. 3i) whereby the protonation occurs on the microsecond timescale, as indicated in the photocycle scheme in Fig. 1a. Therefore, the time-resolved infrared measurements confirmed the *trans*–*cis* photoisomerization as well as the ~10% femtosecond flash yield that corresponds to the concentration measured by flash photolysis (see below) of the final product. Both the time-resolved visible TA and the time-resolved infrared measurements confirm the primary photoproduct of the off state to be the *cis* neutral chromophore, in agreement with the observed microsecond thermal deprotonation that generates the final deprotonated *cis* anion chromophore product (Fig. 1b).

### Flash photolysis photoproduct yield determination

To maximize the photoactive population in the XFEL experiments, flash photolysis measurements were conducted in the home laboratory to determine the ideal pumping conditions. A thin-film crystalline sample of rsKiiro was prepared using a previously described method^64^ at ODs between 0.2 and 0.5. For each measurement, the sample area was fully photoswitched forwards and then backwards using unfocused 473-nm and 400-nm c.w. laser illumination to measure the total photoswitchable population. The sample was fully switched to the off state and then exposed to a single 400-nm, 100-fs pump pulse to measure the photoconversion step, and then once again fully photocycled to measure the non-recoverable bleach. The process was repeated over a range of pulse energy densities at 100 fs (Extended Data Fig. 4a). A fresh sample region was used each time. A pump-pulse energy density of 2 mJ mm^−2^ was selected for XFEL experiments as this was the value that would produce a yield of >10% while keeping the irreversible bleach as small as possible. Using methods described in ref. ^64^, the photoproduct yield was fitted to recover the total effective nonlinear cross-section (Extended Data Fig. 4b,c). Analysis of the power density dependence provided values for the linear and nonlinear optical cross-sections (Extended Data Fig. 4b,c). Although excited-state absorption was not avoided, internal conversion followed Kasha’s rule to produce a vibrationally excited S_1_ population in addition to the S_1_ population formed by linear photoexcitation.

To confirm the effect of dumping on the final photoproduct yield, flash photolysis measurements were conducted at a fixed pulse energies while the pump–dump delay was scanned. Extended Data Fig. 4d shows that the photoproduct yield is suppressed by illumination of the dump pulse after the pump pulse and that the form of the suppression follows the approximate shape of the cross-correlation of the two 100-fs pulses and the excited-state decay time constants obtained from TA (Extended Data Fig. 3d).

## Online content

Any methods, additional references, Nature Portfolio reporting summaries, source data, extended data, supplementary information, acknowledgements, peer review information; details of author contributions and competing interests; and statements of data and code availability are available at 10.1038/s41557-023-01275-1.

### Supplementary Information

Supplementary Information Supplementary methods, discussion, Figs. 1–63, Tables 1–11 and references.

## Figures and Tables

**Extended Data Table 1:**
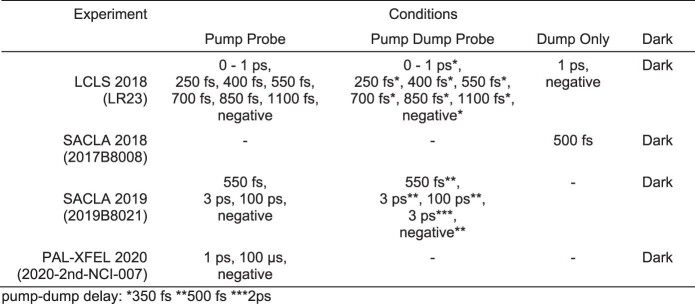
Summary of TR-SFX datasets collected during this work

**Extended Data Table 2:**
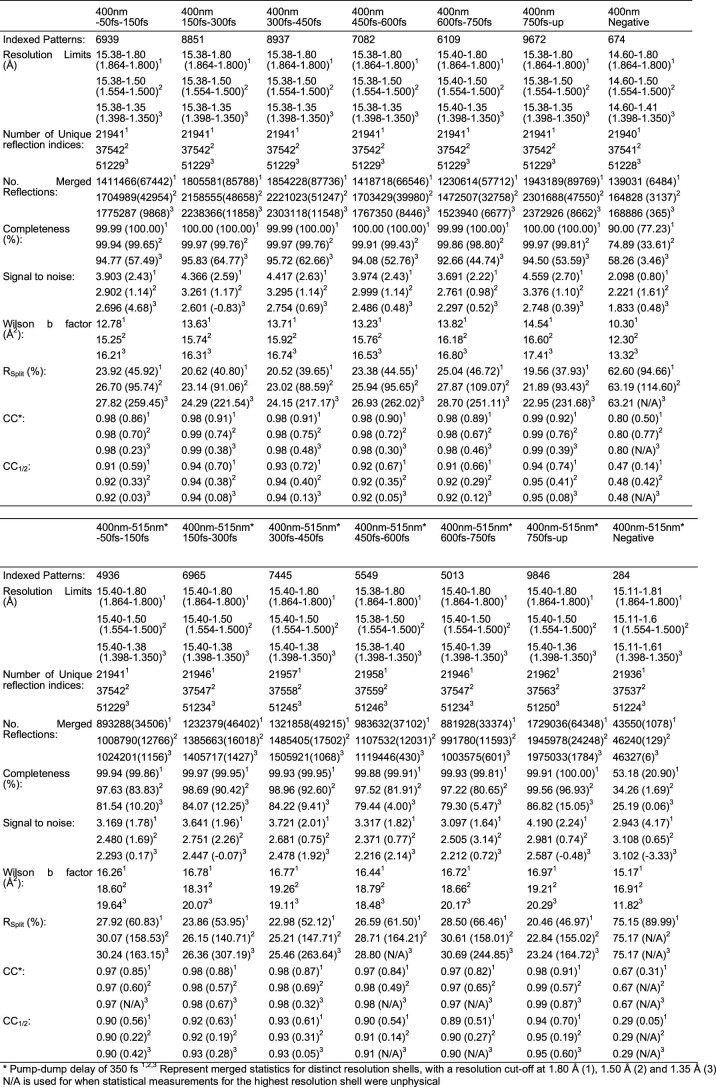
LCLS LR23 TR-SFX PP & PDP crystallographic statistics for sub-ps time bins

## Data Availability

The data that support the findings of this study are available on request from the corresponding author. The extensive data collection generated numerous raw data files. The results have been summarized in the main and supplementary figures and, due to the large number of raw data files, these are not provided with the Article, but are available from the corresponding author on request. Coordinates and structure factor amplitudes have been deposited to the PDB database. The rsKiiro *cis* structure at 290 K can be found at MX Data (PDB 7QLI). The rsKiiro *trans* structure with illumination at 290 K can be found at MX Data (PDB 7QLJ). The rsKiiro *cis* structure intermediate at 200 K can be found at MX Data (PDB 7QLK). The rsKiiro thermal annealing at 290 K of the 200 K *cis* intermediate can be found at MX Data (PDB 7QLL). The rsKiiro dark reference SFX coordinates for the *trans* off state are available at PDB 7QLM. The rsKiiro pump–probe picoseconds averaged coordinates are available at PDB 7QLN. The rsKiiro PDP picoseconds averaged coordinates are available at PDB 7QLO. Hydrogen-bonding configuration structures obtained from QM/MM (Extended Data Fig. 8) are available at 10.5281/zenodo.7887626.

## Code availability

Computer codes for the density matrix calculations and Wigner transforms used for the results shown in Fig. 4 and Supplementary Figs. 54, 55, 56, 57, 58, 59, 60, 61, 62 and 63 are available at https://github.com/perrettsamuel/Wigner-Transform-of-Density-Matrix.
