# Supplementary material for: Optical control of ultrafast structural dynamics in a fluorescent protein
Source: Nat Chem. 2023 Aug 10;15(11):1607–15. doi: 10.1038/s41557-023-01275-1 (PMC10624617; doi:10.1038/s41557-023-01275-1)
Supplement: Supplementary file 1 — Supplementary methods, discussion, Figs. 1–63, Tables 1–11 and references. [file 41557_2023_1275_MOESM1_ESM.pdf]

nature chemistry

Article https://doi.org/10.1038/s41557-023-01275-1

Optical control of ultrafast structural dynamics in a fluorescent protein

In the format provided by the authors and unedited

nature chemistry

Article https://doi.org/10.1038/s41557-023-01275-1

Optical control of ultrafast structural dynamics in a fluorescent protein

In the format provided by the authors and unedited

This PDF file includes:

Supplementary Methods **2**

1. Micro crystal breaking with glass beads 2
2. SFX detector distance optimization 3
3. Time zero determination by cross correlation. 7
4. Femtosecond Optical Pulse Characterization 8
5. TR-SFX datasets 12
   5.1. LCLS CXI LR23 12
   5.2. SACLA 2017B8008 27
   5.3. SACLA 2019B8021 29
   5.4. PAL XFEL 2020-2nd-NCI-007 35
6. Photo conversion quantum yield measurements 38
7. Time-correlated single photon counting (TCSPC) 38
8. Fluorescence spectra and fluorescence quantum yield 40
   8.1. Fluorescence lifetime estimate from Strickler Berg relation 41
9. Cyro-trapping of rsKiiro intermediates 41
   9.1. Steady state MX Cryo-structures 41

Supplementary Discussion **47**

10. rsKiiro development 47
11. Thermodynamic modelling of rsKiiro photo-switching 49
    11.1. Analytical solution to the overall off → on conversion process using the Master Rate Equation method. 49
    11.2. Summary of theoretical considerations for temperature dependence of the trans-cis photoisomerization 51
    11.3. Modelling of the temperature dependence of cis-trans and trans-cis photoisomerization 52
    11.4. Ground state evolution in the off → on direction 54
    11.5. Modelling of the convex Arrhenius behaviour of the on→off and off→on photoconversion 55
    11.6. Explicit thermodynamics treatment of barriers in the reactive coordinate and non-radiative transitions 57
12. Hybrid Quantum Mechanics / Molecular Mechanics Computations 61
13. Density Matrix Simulations & Wigner Phase Space Analysis 63
    13.1. Density Matrix Simulations 65
    13.2. Wigner Phase Space Analysis 66
    13.3. Simulation Parameters 68
    13.4. Pump vs Pump-Dump scheme 70
    13.5. Pump-Dump delay comparison 75
    13.6. Wavepacket assignment and discussion of coherence parameters and simulation 76
14. Supplementary references 82

Supplementary Methods

1. Micro crystal breaking with glass beads

To reduce issues with blockages during crystal injection particularly with the GDNV injection it was necessary to optimise the size distribution of the crystals, this was done by breaking up the microcrystals with glass beads.

1 mL of slurry in either 25% or 30% PEG 3350 condition was added to 0.5 mL of pre-rinsed glass beads of size <106 µm (Sigma-Aldrich) in a 2 mL round bottom Eppendorf tube before vortexing for 10 seconds at room temperature and keeping on ice for 1 minute. After a total of 15 breaking and cooling cycles, the mixture was spun down at 1000 x g for 10 s and the crystal-containing supernatant carefully pipetted away and poured in a 50 µm CellTrics filter (Sysmex Partec GmbH, Germany). The glass beads were then washed four times with 0.25 mL of 25% (or 30%) PEG 3350 in Tris and Lithium Sulfate buffer and spun down at 1000 x g and the supernatant loaded onto the 50 µm filter each time. The PEG 3350 concentration in the final filtered slurry was set as desired by changing that in the Tris and Lithium Sulfate buffer during the beads-washing step. The 2 mL filtered slurry was either left to settle at 20°C overnight or straight away loaded onto a 30 µm CellTrics filter. The crystal breakage procedure removed clumps and yielded a narrower crystal size range with maximum final crystal dimensions of the order of 3 x 5 x 30 µm.

Since crystals in slurries prepared with 25% PEG 3350 tended to settle in the reservoir and clog the injector tubing and GDVN injector, they were spun down at 1,000 x g for 10 s and exchanged in 30% PEG 3350 by adding a 32.5% PEG3350 buffer solution to the sedimented crystals in 2:1 ratio and adjusting the volume before measuring the crystal density.

2. SFX detector distance optimization

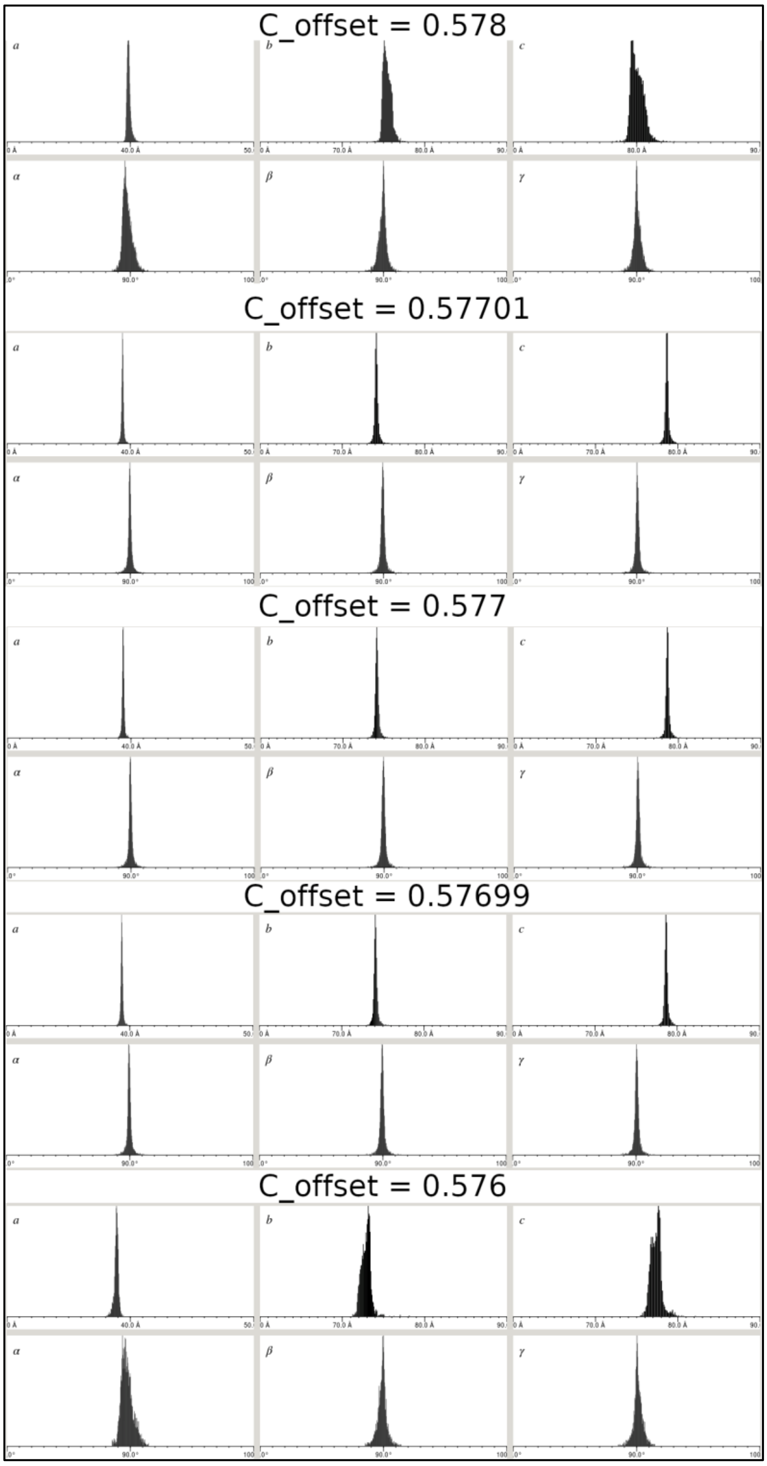

Supplementary Fig. 1 | Crystallographic unit cell optimization scan at LCLS LR23. The unit cell distribution data was highly sensitive to detector distance. Shown are the resulting distributions for the C_offset parameter values between 0.576 and 0.578.

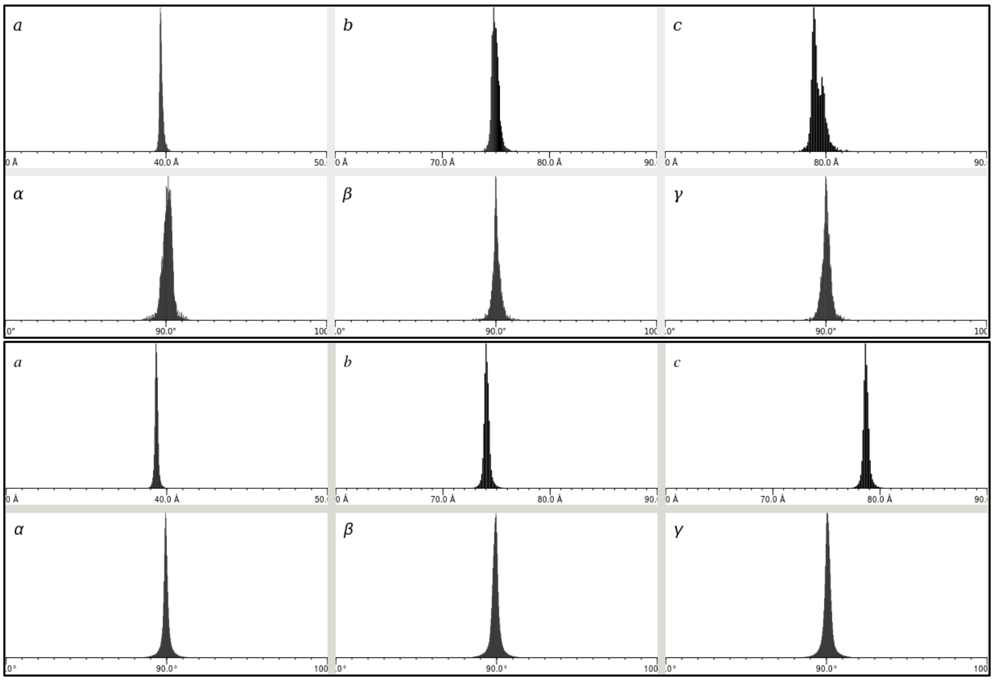

Supplementary Fig. 2 | Crystallographic unit cell optimization comparison at LCLS LR23. Comparison of the rsKiiro un-optimised (top) and optimized (bottom) unit cell distributions for LR23 beamtime.

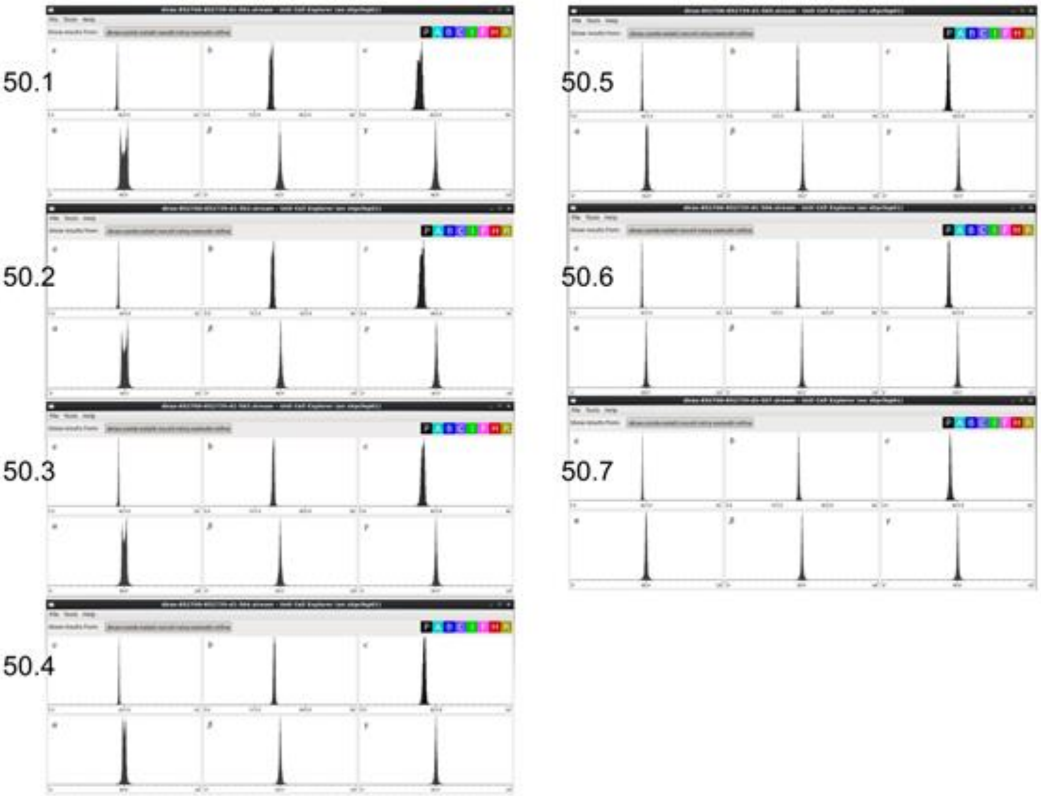

Supplementary Fig. 3 | Crystallographic unit cell optimization scan at SACLA 2019B8021. A systematic scan of the camera distance between 50.1 mm and 50.7 mm also revealed a very strong sensitivity of the cell distribution and splitting of primarily the c-dimension as well as the alpha angle. The narrowest distributions at 50.7 mm also led to the best L-test results with ~ 4% twin fraction calculated, and the optimum R-factors.

50.5

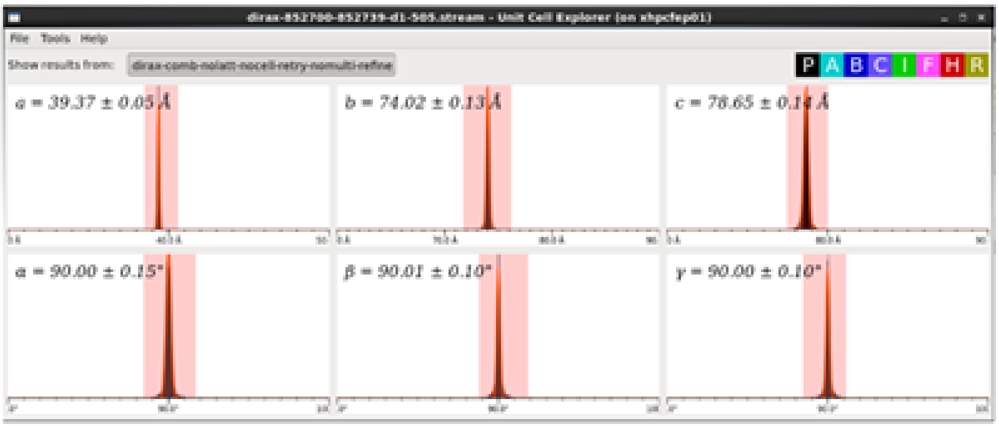

50.6

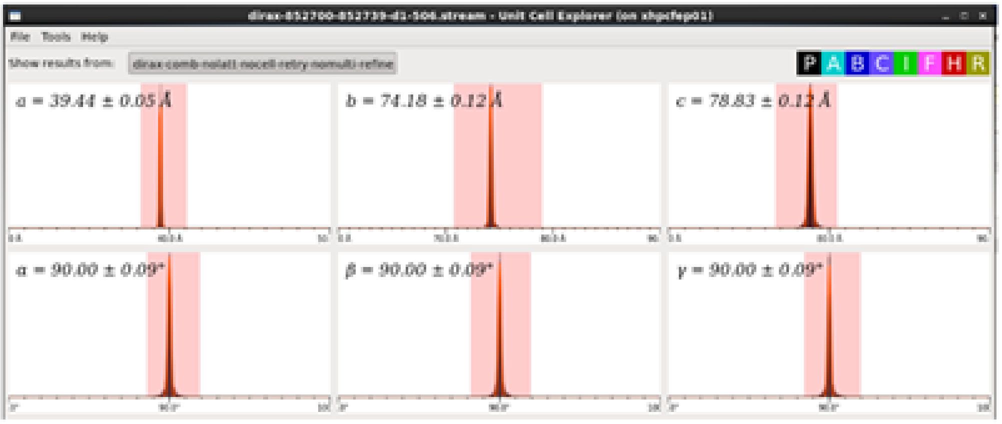

50.7

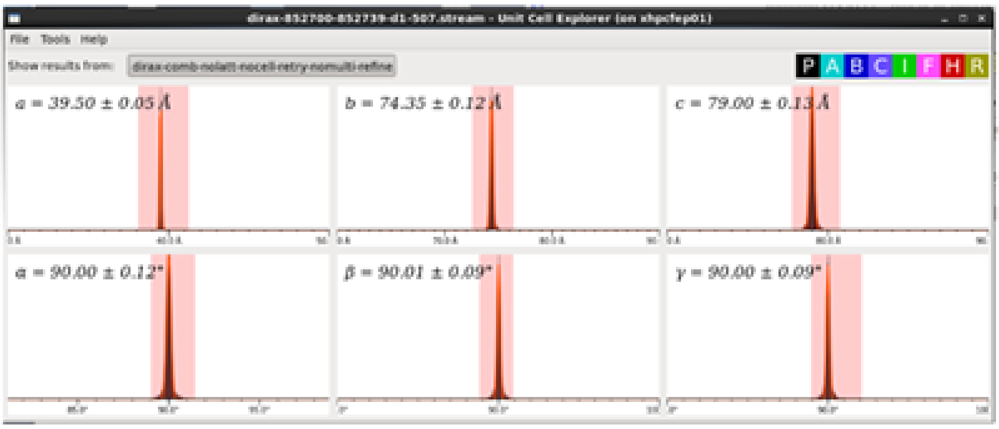

Supplementary Fig. 4 | Crystallographic unit cell optimization scan at SACLA 2019B8021,Values near 50.6 mm resulted in the narrowest distributions, loss of split cell parameters, and L-test calculation showed levels of potential twinning at or below 4%

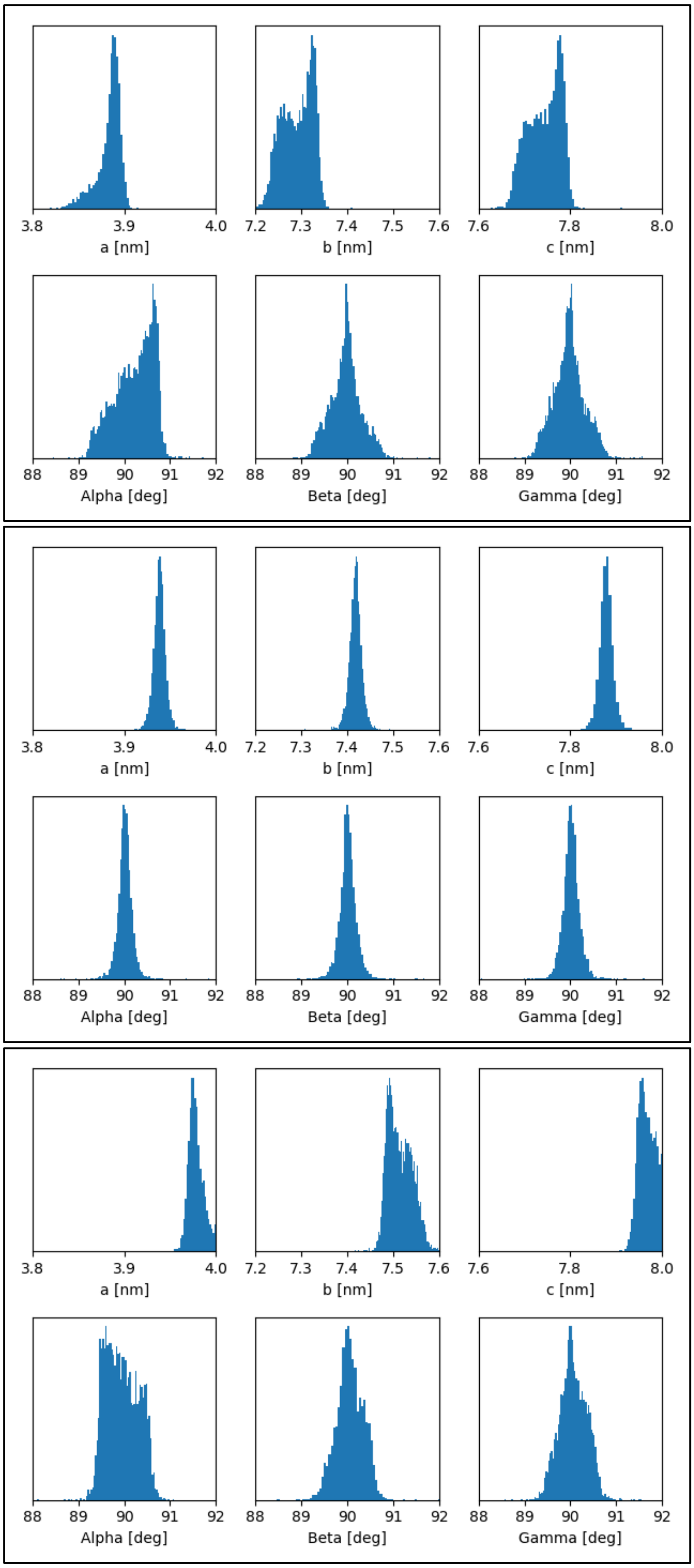

Supplementary Fig. 5 | Crystallographic unit cell optimization scan at PAL XFEL 2020-2nd-NCI-007. Histogram of unit cell distributions with different detector distances of 141.0, 142.9 and 144.5 mm for the top, middle, bottom figures respectively. For each detector distance the fitted cell dimensions were 39.36±0.06 Å, 74.15±0.13Å, 78.70±0.13 Å and angles of 90±0.13°, 90±0.15°, 90.02±0.19°.

3. Time zero determination by cross correlation.

At each of XFEL beamtimes the temporal delay between the X-rays and both the pump and dump optical pulses delay was measured using the standard cross-correlation technique exploiting the transient reflectivity [1] exhibited by semiconductors when exposed to hard X-rays. In each case a Ce:YAG scintillator screen that was used to achieve spatial overlap was used as the transient reflectivity medium, 20 µm thick at LCLS and 50 µm at SACLA. The XFEL beam was expanded by removing several lenses from the beryllium compound refractive lenses (CRL) stack to match the spot sizes of the optical beams to maximize the amplitude of the cross-correlation signal. The optical beam and X-rays were overlapped on the Ce:YAG screen and the transmission of the optical beam was measured using photodiode while the temporal delay between the two was scanned. With significant averaging a sigmoid-like change in transmission is seen roughly centred when the pulses are temporally overlapped. To improve the accuracy of the cross-correlation the output of each facility's timing tool was used to correct for the XFELs inherent temporal jitter (Supplementary Fig. 6). Finally the time corrected data was binned and fitted using the methods previous described in [2]. These cross correlations scans were made at the start of each beamtime and repeated multiple times throughout each data collection to check for slow temporal drift between the two optical arms and the X-rays. These measurements were also the most accurate way to set and confirm the relative delay between the pump and dump pulses at the interaction region which was ~350 fs at CXI LR23 and ~500 fs at 2019B8021 (Supplementary Fig. 6).

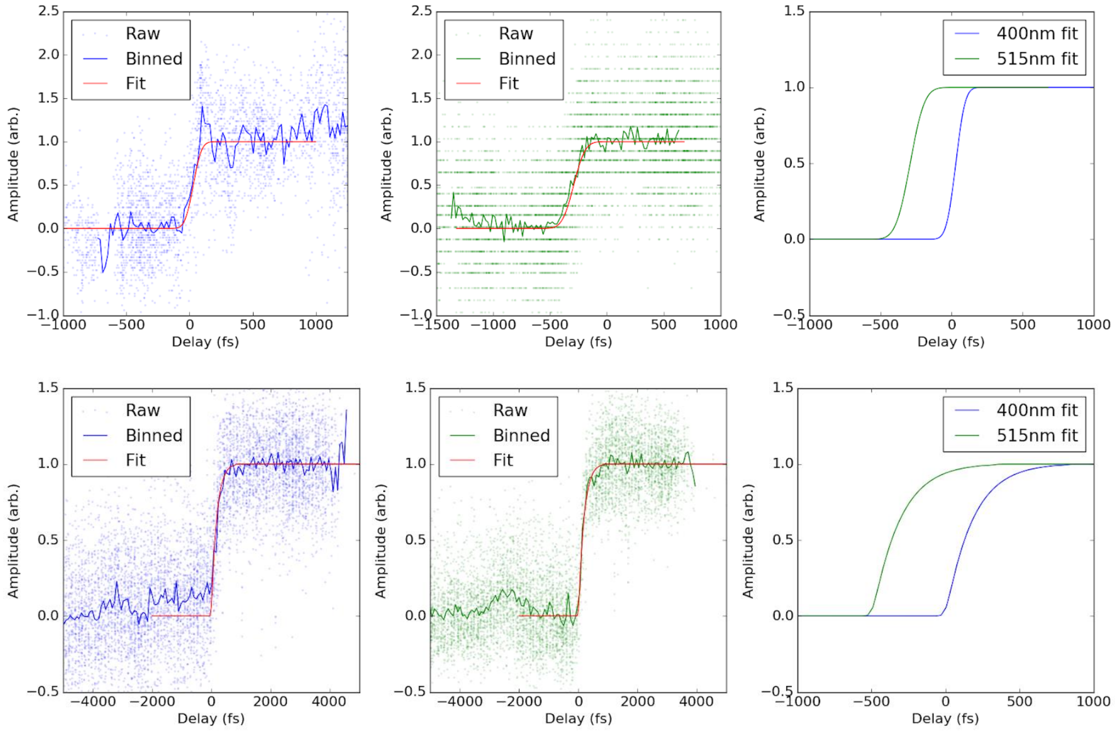

Supplementary Fig. 6 | TR-SFX cross correlation time zero determination. Shown for LCLS LR23 (top row) and SACLA 2019B8021 (bottom row). In the 400 nm pump (left column) and 515 nm dump (middle column) pulses, each show the raw photodiode signal after timing tool correction of the XFEL jitter was applied (dots), binned trace (line) and fitted sigmoid (red line). The fit was generated using the methods previous described[2]. The comparison of the fits (right column) shows the pump-dump delay. The delay at LR23 was determined to be ~350 fs while that at 2019B8021 was 500 fs. Note: the order of the optical pulses appears counter intuitive (i.e the 515 nm pulse appears first) in the cross-correlation measurement X-ray pulse is the “pump” and the optical pulse is the “probe” as opposed to the TR-SFX

experiment where the situation is reverse hence the temporal order of the optical pulses is effectively reversed when scanning the same optical delay stage.

4. Femtosecond Optical Pulse Characterization

To confirm spectral and temporal likeness of the optical excitation pulses provided by the onsite SACLA/LCLS lasers to those used for spectroscopic measurements in the homelab two compact homebuilt frequency resolved optical gating (FROG)[3] setups were deployed at the beamlines. A second harmonic generation based (SHG-FROG)[4] to characterize the 515 nm dump pulses and a cross correlation based (XFROG) for the 400 nm pump pulses. The more complicated XFROG was required for the 400 nm pulses due to the phase matching limitations of SHG non-linear crystals. Schematics of the two devices are shown in Supplementary Fig. 7. A type II 20 µm thick surface mounted BBO θ=29.2° (Newlight Photonics Inc.) was used for characterizing the 800 nm fundamental. A type II 20 µm thick surface mounted BBO (θ=55°) was used for SHG-FROG of the 515 nm and XFROG of the 400 nm, the phase matching angle of the crystal was adjusted in each case maximize the generated signal.

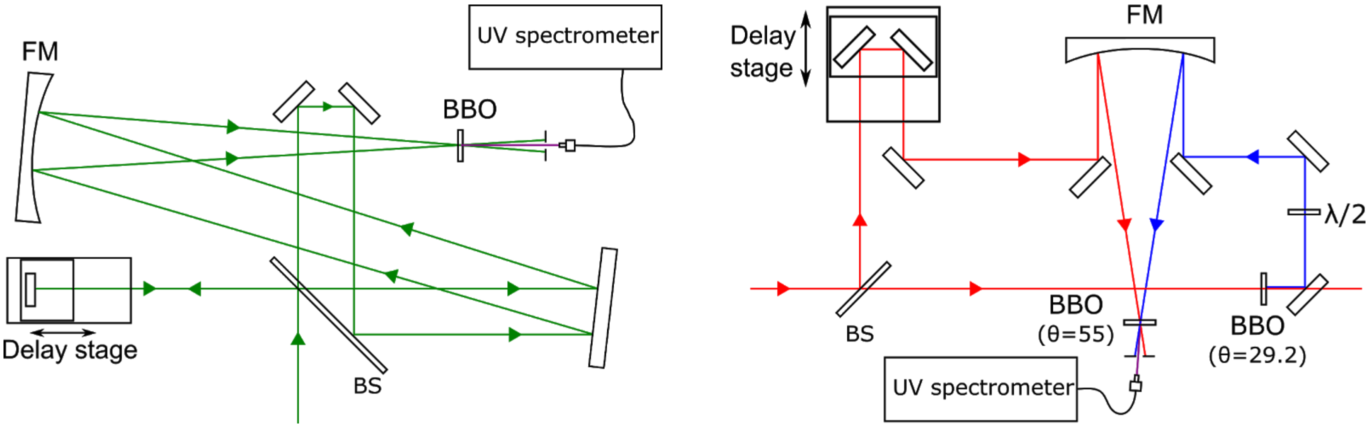

Supplementary Fig. 7 | FROG and XFROG schematics. SHG-FROG (left) and XFROG (right) used for pulse characterization during TR-SFX beamtimes. Where BS and FM are a pellicle beam splitter and f=200 mm focusing mirror. The SHG-FROG was used for both measurement of the 800 nm fundamental and 515 nm dump pulses by substituting the appropriate BBO crystal and spectrometer.

The results of the FROG retrieval for LR23 and 2019B8021 are shown in Supplementary Table. 1 and the individual fits are shown in Supplementary Fig. 8 - Supplementary Fig. 10. During LR23 difficulties with other parts of the experiment limited the time to perform FROG characterization as a result no signal was obtained from the XFROG therefore the 400 nm pump characteristics are estimated based on those obtained from the fundamental 800 nm measured after passing through the same 100 µm BBO used to generate the 400 nm pulses. At 2019B8021 the pulses are shorter than those used at LCLS this could help explain the reduced strength of signals in the SFX data[5]. In addition while an XFROG trace was obtained the retrieve a pulse width is far shorter than the fundamental used to generate it, we recognize that this is aphysical and more likely due to misalignment in the XFROG cropping the spectra.

In addition to the FROG characterisation of laser conditions at XFEL experiments, we have additionally measured and characterised the power density dependence of product formation. Analysis of the power density dependence provided values for the linear and non-linear optical cross sections (Extended Data Fig. 2b & c). While excited state absorption was not avoided, internal conversion followed Kasha's rule

to produce vibrationally excited S_1 population in addition to the S_1 population formed by linear photoexcitation.

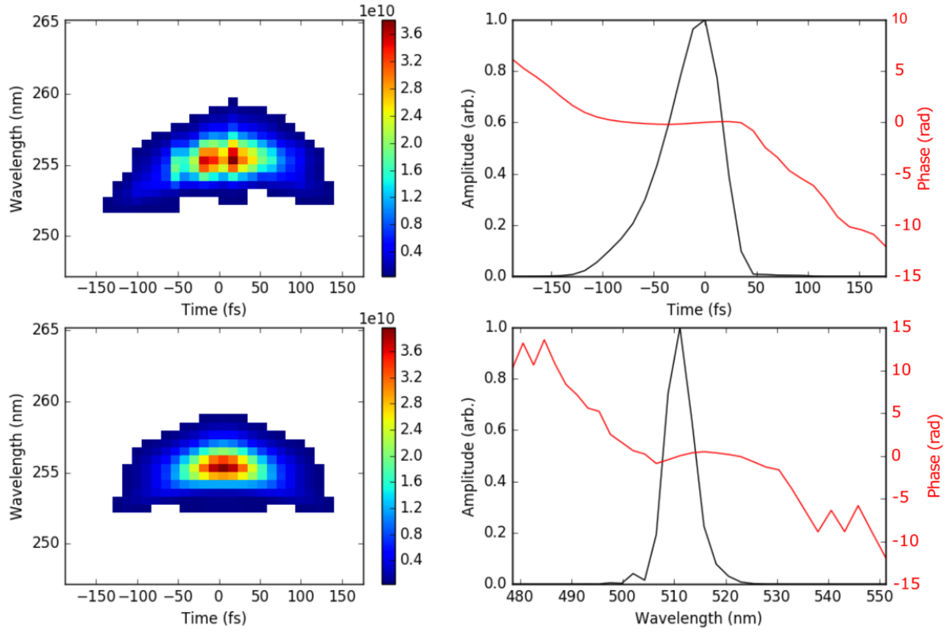

Supplementary Fig. 8 | LCLS LR23 515 nm dump FROG fitting. TOPAS output. SHG-FROG trace experiment (top left), reconstruction (bottom left), recovered pulse temporal profile (top right) and spectral profile (bottom right)

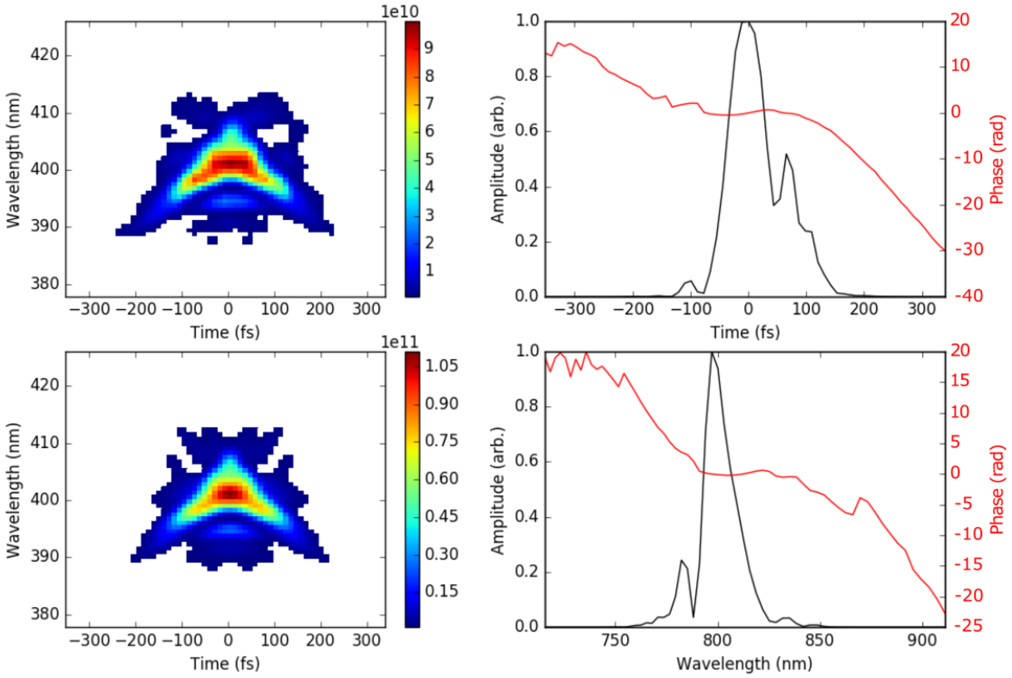

Supplementary Fig. 9 | LCLS CXI LR23 800 nm fundamental FROG fitting. SHG-FROG trace experiment (top left), reconstruction (bottom left), recovered pulse temporal profile (top right) and spectral profile (bottom right)

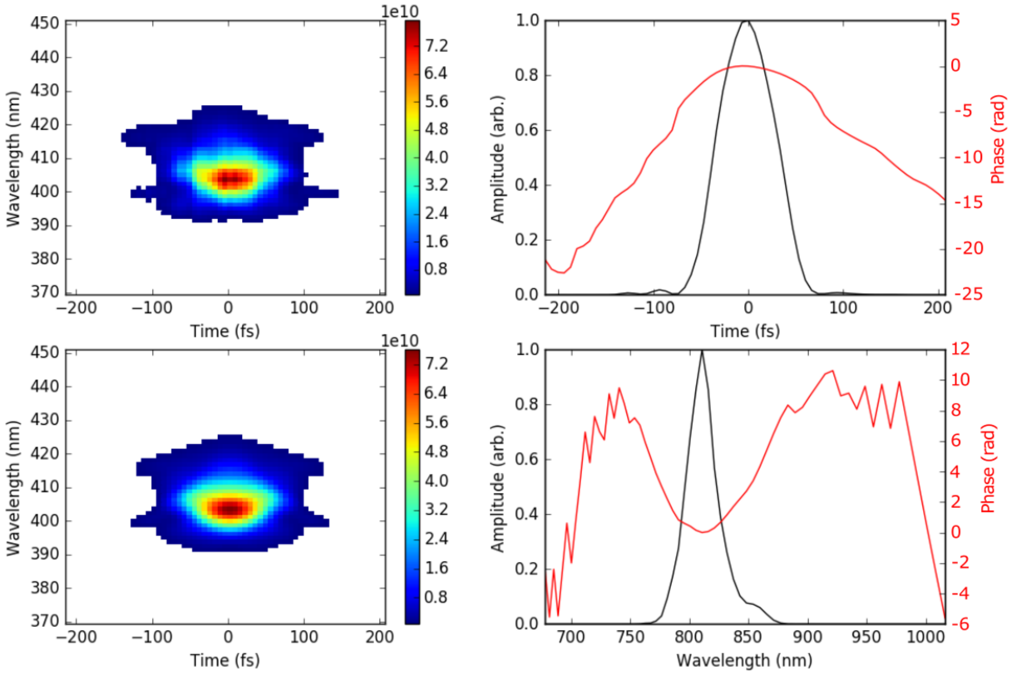

Supplementary Fig. 10 | SACLA 2019B8021 800 nm fundamental FROG fitting. SHG-FROG trace experiment (top left), reconstruction (bottom left), recovered pulse temporal profile (top right) and spectral profile (bottom right).

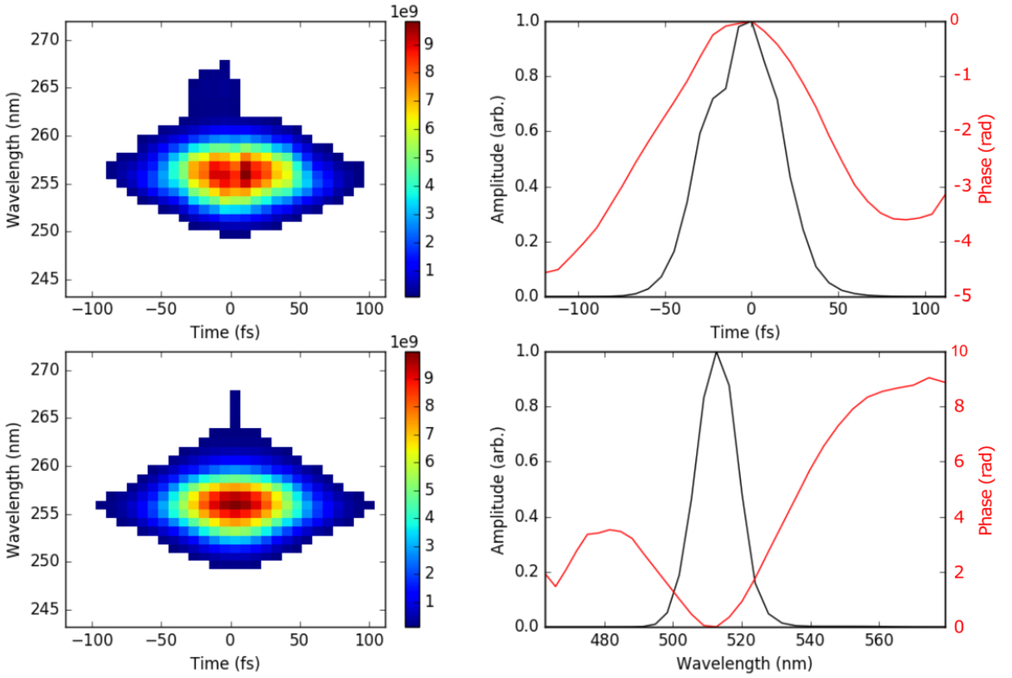

Supplementary Fig. 11 | SACLA 2019B8021 515 nm dump FROG fitting. Showing SHG-FROG trace experiment (top left), reconstruction (bottom left), recovered pulse temporal profile (top right) and spectral profile (bottom right).

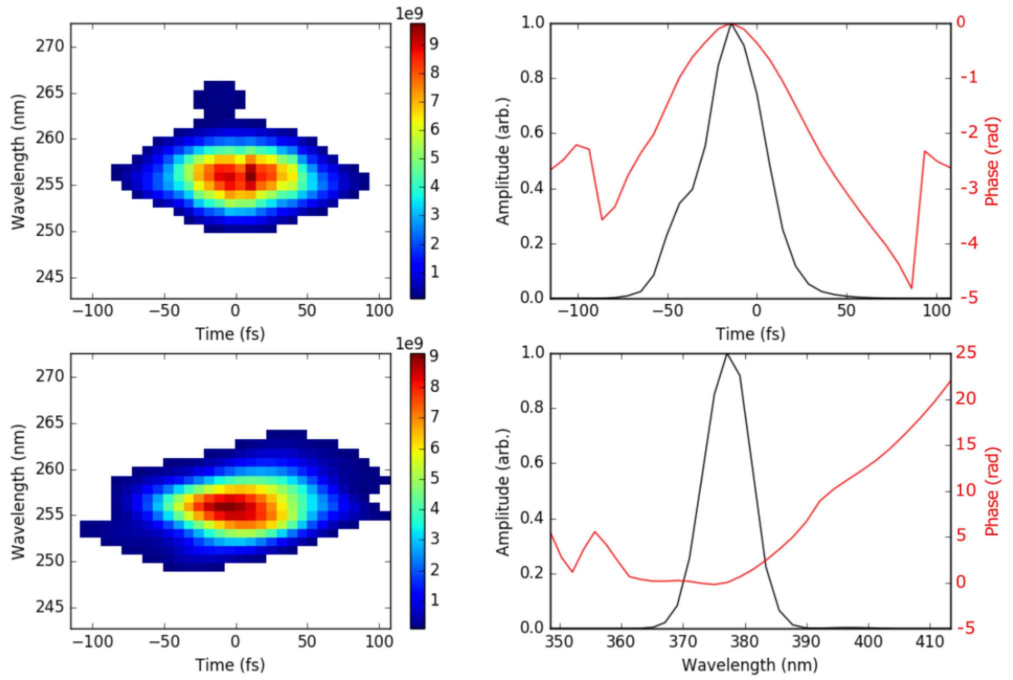

Supplementary Fig. 12 | SACLA 2019B8021 400 nm pump FROG fitting. Showing XFROG trace experiment (top left), reconstruction (bottom left), recovered pulse temporal profile (top right) and spectral profile (bottom right).

Supplementary Table. 1 | FROG fitting results. Results of SHG-FROG and XFROG pulse fitting for LCLS LR23 and SACLA 2019B8021 beamtimes.

		λ (nm)	Δλ (nm)	Δt (fs)	GDD (fs^2)	3rd Order (fs^3)
LCLS						
	Fundamental (after BBO)	800	15.5	100 ± 15	554 ± 50	2944 ± 600
	Pump	400	-	~100	-	-
	Dump	515	6.8	71 ± 15	480 ± 40	2000 ± 400
SACLA						
	Fundamental (after BBO)	800	27	72	262 ± 12	N/A
	Pump	400	9	40	135 ± 15	N/A
	Dump	515	14	52	355 ± 175	184 ± 20

5. TR-SFX datasets

A summary of all the different SFX datasets collected over the course of this study is shown in Extended Data Table 1.

5.1. LCLS CXI LR23

Figure 2 shows data from the LR23 experiment at LCLS beamline CXI. The Fourier shell Coefficient (FSC) and R-split for all merge laser off and dark data is shown in Supplementary Fig. 13. Estimation of the Dark (only preilluminated) occupancy is shown in Supplementary Fig. 29. N_{EXT} determination and occupancy refinement was performed on the combined 0-1 ps PP data (Supplementary Fig. 14 - Supplementary Fig. 15). The anisotropic ratio of the fully merged dark, PP and PDP is shown in Supplementary Table. 2. Using the output of the CXI timing tool the sub picosecond data was sorted into 150 fs wide bins. Occupancy refinement was performed for each of time bin for the PP (Supplementary Fig. 17 - Supplementary Fig. 22) and PDP (Supplementary Fig. 23 -Supplementary Fig. 28) datasets. The method used to determine occupancy for the pumped coordinates used here, is validated by scanning the occupancy of PDP coordinates for the dark unpumped data only. As expected, the addition of also small contributions of PDP coordinates increases the R-factor as expected, as shown in Supplementary Fig. 29.

Comparison of the PP and PDP bins shown in Extended Data Fig. 6, 7 and Supplementary Fig. 30. Supplementary Fig. 31 shows Pump-Probe and Pump-Dump-Probe Q-weighted Fo-Fo maps from the rsKiiro TR-SFX experiment. In the top row, Q-weighted Fo-Fo maps are shown for all of the collected PP, PDP, and dark data (as in Figure 2 of the main text). For the rows below, all datasets were split in two halves and Q-weighted Fo-Fo maps were recomputed for different combinations of the obtained structure factors. The amplification of signals in the Pump-Dump-Probe data compared to the Pump-Probe data is reproducibly shown across the different examples Supplementary Fig. 32 shows difference structure factor amplitudes for PP and PDP along with weighted assigned to each difference by Q-weighting. Crystallographic statistics are shown Extended Data Table 2 & 3.

Fo-Fo difference maps are calculated with the Q-weighted technique, and the weighted differences are used to calculated the extrapolated structure factors. With Q-weighting, the differences are found in the following way

$$WDF = w \times \Delta F$$

Where

$$\Delta \mathrm{F} = \left|\mathrm{F}_{\mathrm{obs}_\mathrm{L}}\right| - \left|\mathrm{F}_{\mathrm{obs}_\mathrm{D}}\right|$$

And

$$w = \left(1 + \frac{\sigma_{\Delta \mathrm{F}}^2}{\overline{\sigma_{\Delta \mathrm{F}}^2}} + \alpha \frac{|\Delta \mathrm{F}|^2}{\overline{|\Delta \mathrm{F}|^2}}\right)^{-1}$$

The scale factor α decreases the influence of structure factor differences that have very large magnitudes while maintaining what may be erroneously small uncertainties. In this work, a value of α= 1 was implemented as in Šrajer et al. (2001)[6]. Earlier work on Q-weighting by Ursby and Bourgeois (1997)[7] used a value of α= 0.

Extrapolated structure factors were calculated using the Q-weighted differences according to Pandey et al.(2020)[8] and Gorel, Schlichting, & Barends(2021)[9]

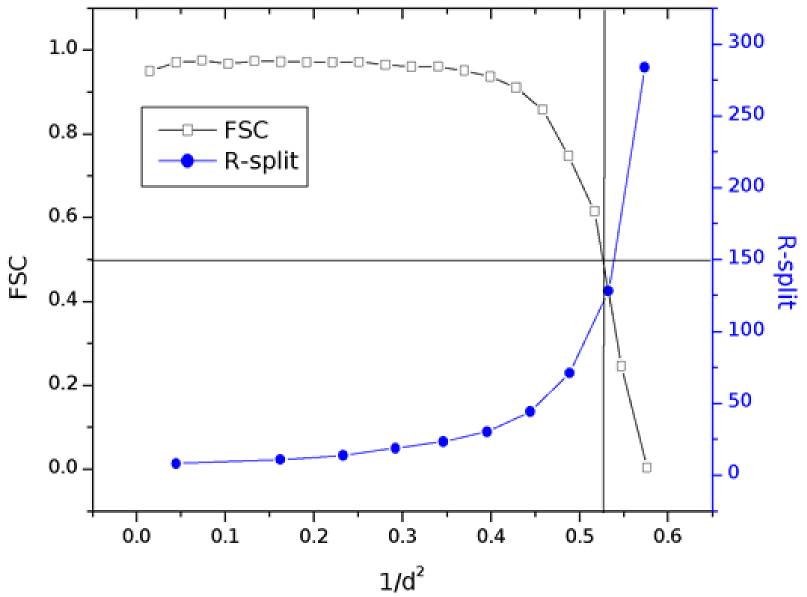

Supplementary Fig. 13 | LCLS LR23 FSC and R-Split for the all merged laser off, dark data. An FSC of 0.5 is chosen for a limiting 1.38 Å.

Supplementary Table. 2 | Anisotropic analysis by STARANISO[10] for merged 0-1 ps PP and PDP datasets.

Dataset	Anisotropic ratio	Diffraction limits of anisotropic ellipsoid (a*,b*,c*) [Å]
Dark	0.161	1.489, 1.400, 1.404
PP (400 nm)	0.141	1.489, 1.410, 1.404
PDP (400 and 515 nm)	0.140	1.572, 1.518, 1.498

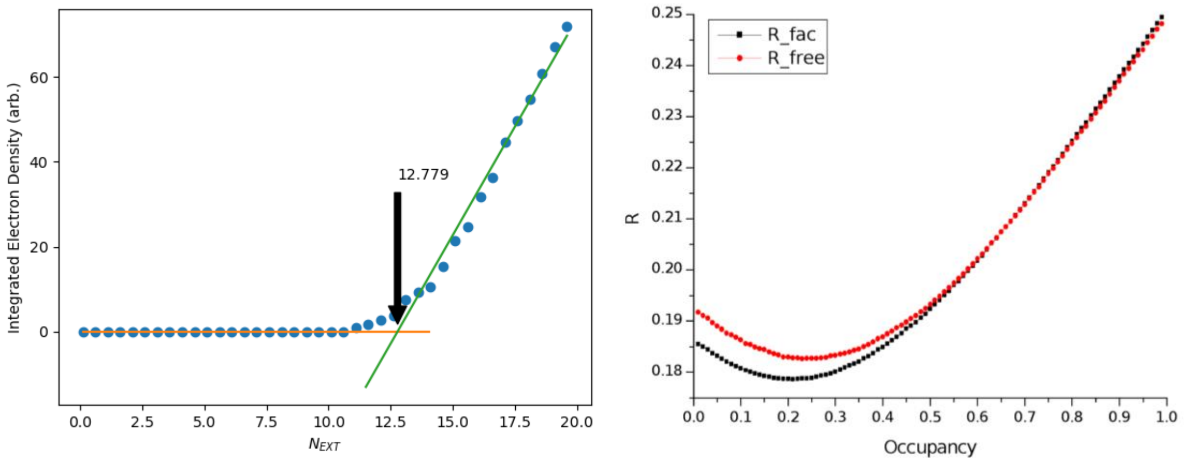

Supplementary Fig. 14 | LCLS LR23 PDP 400-515 nm merged 0-1 ps: Left: Extrapolated factor, N_{EXT}, determination through the intersection point of two linear fits to the magnitude of integrated negative electron density, shown as blue circles. Right: R_fac/R_free calculation for varied occupancy

of refined coordinates. Occupancy refers to the proportion of extrapolated coordinates (light) compared to ground state coordinates (dark). Both R_{fac} and R_{free} minimise at dark=0.79 and light=0.21.

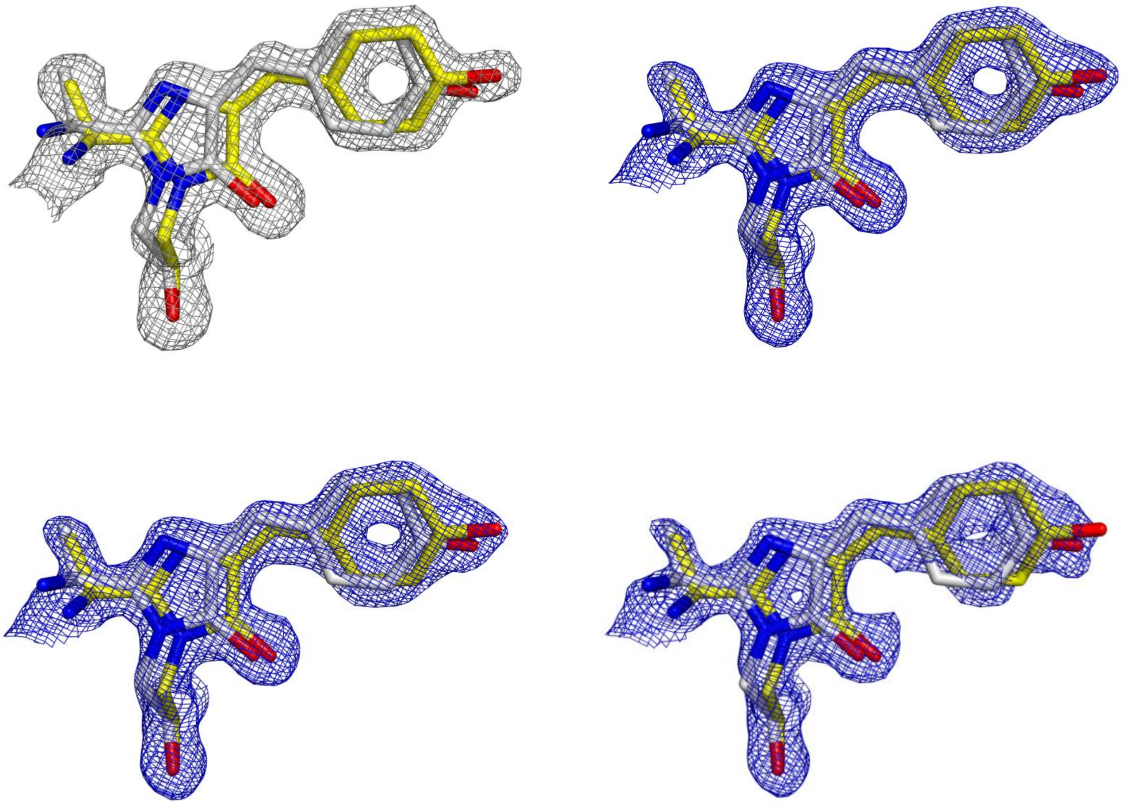

Supplementary Fig. 15 | LCLS LR23 PDP 400-515 nm merged 0-1 ps: Refined coordinates for the ground state (yellow) and the extrapolated map coordinates for PDP (grey). Top left: 2Fo -Fc electron density for at 1.5 rms contouring (white) for the 400 nm 0 - 1 ps bin with occupancy of 0.86 ground-state and 0.14 extrapolated. Top Right: F_o electron density for at 1.5 rms contouring (blue) for the extrapolated value of N_{EXT} = 5.1. Bottom Left: F_o electron density for at 1.5 rms contouring (blue) for the extrapolated value of N_{EXT} = 7.6. Bottom Right: F_o electron density for at 1.5 rms contouring (blue) for the extrapolated value of N_{EXT} = 12.6.

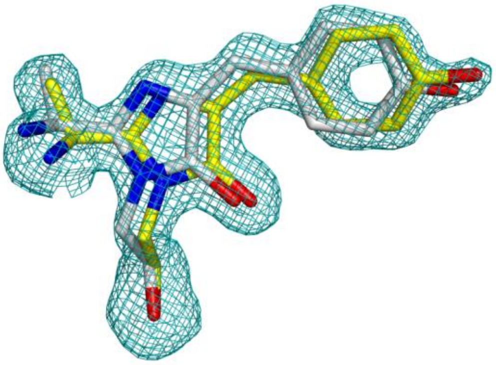

Supplementary Fig. 16 | LCLS LR23 PDP 400-515 nm merged 0-1 ps: Chromophore omitted Fo-Fc density contoured at +3σ level (teal) for 0.79:0.21 (ground:light) occupancy model. The coordinates refined to an extrapolated value of N_{EXT} = 9 are shown in yellow (Ground) and in grey (PDP).

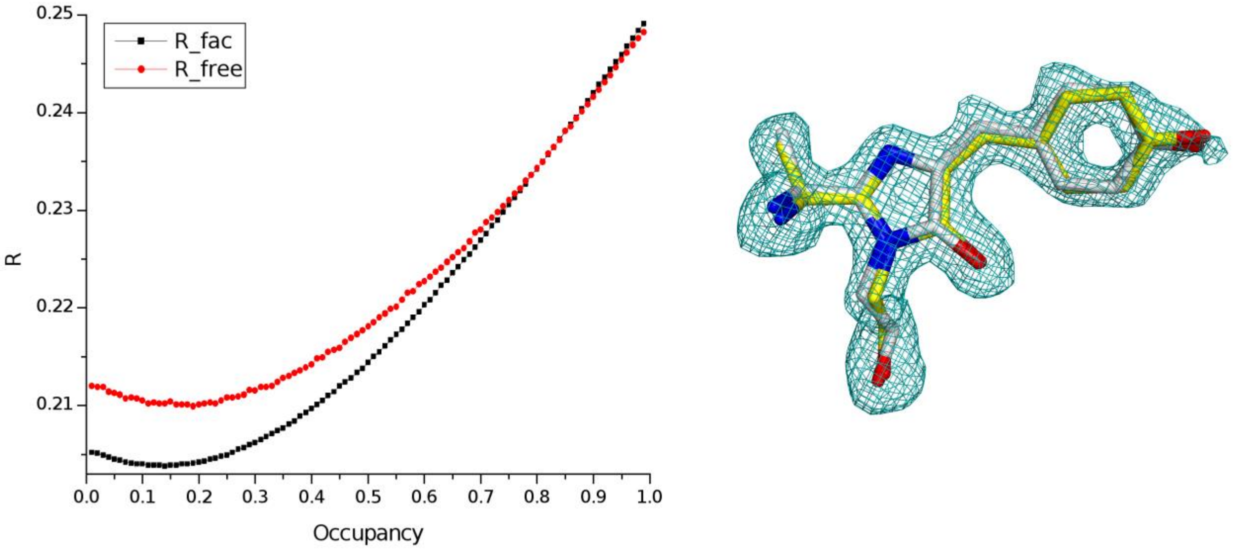

Supplementary Fig. 17 | LCLS LR23 PP 400 nm 250 fs. Left: R_{fac}/R_{free} calculation for varied occupancy of refined coordinates. Occupancy refers to the proportion of extrapolated coordinates (light) compared to ground state coordinates (dark). Both R_{fac} and R_{free} minimize at dark = 0.86 and light = 0.14. Right: Chromophore omitted F_o-F_c density contoured at +3σ level (teal) for 0.86:0.14 (ground:light) occupancy model. The coordinates refined to an extrapolated value of N_{EXT} = 8.6 are shown in grey (light) and ground state in yellow.

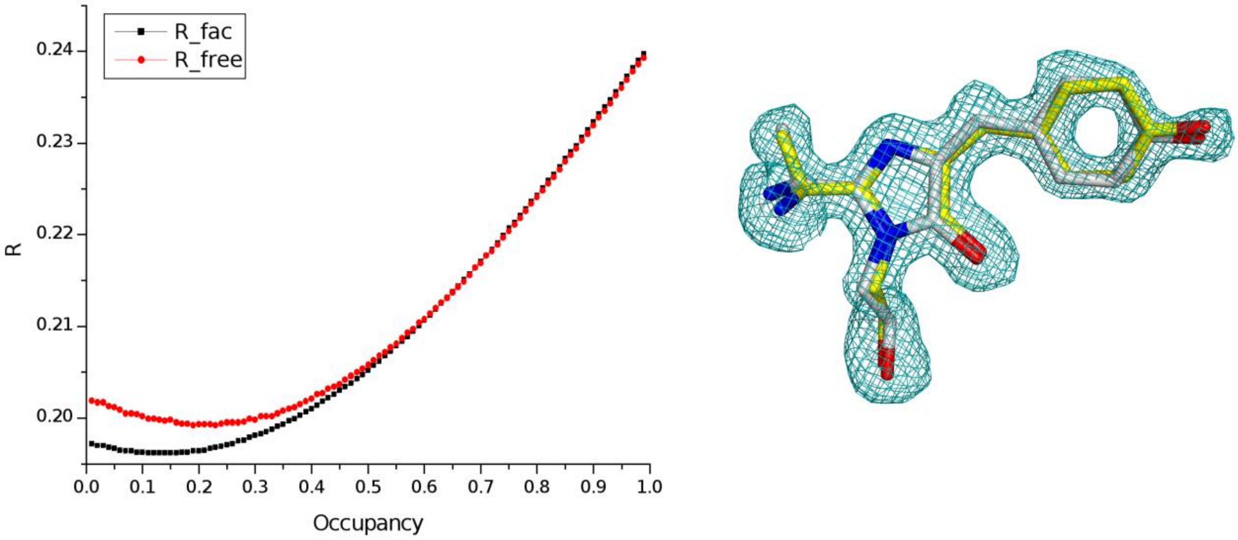

Supplementary Fig. 18 | LCLS LR23 PP 400 nm 400 fs. Left: R_{fac}/R_{free} calculation for varied occupancy of refined coordinates. Occupancy refers to the proportion of extrapolated coordinates (light) compared to ground state coordinates (dark). Both R_{fac} and R_{free} minimize at dark = 0.87 and light = 0.13. Right: Chromophore omitted F_o-F_c density contoured at +3σ level (teal) for 0.87:0.13 (ground:light) occupancy model. The coordinates refined to an extrapolated value of N_{EXT} = 8.6 are shown in grey (light) and ground state in yellow.

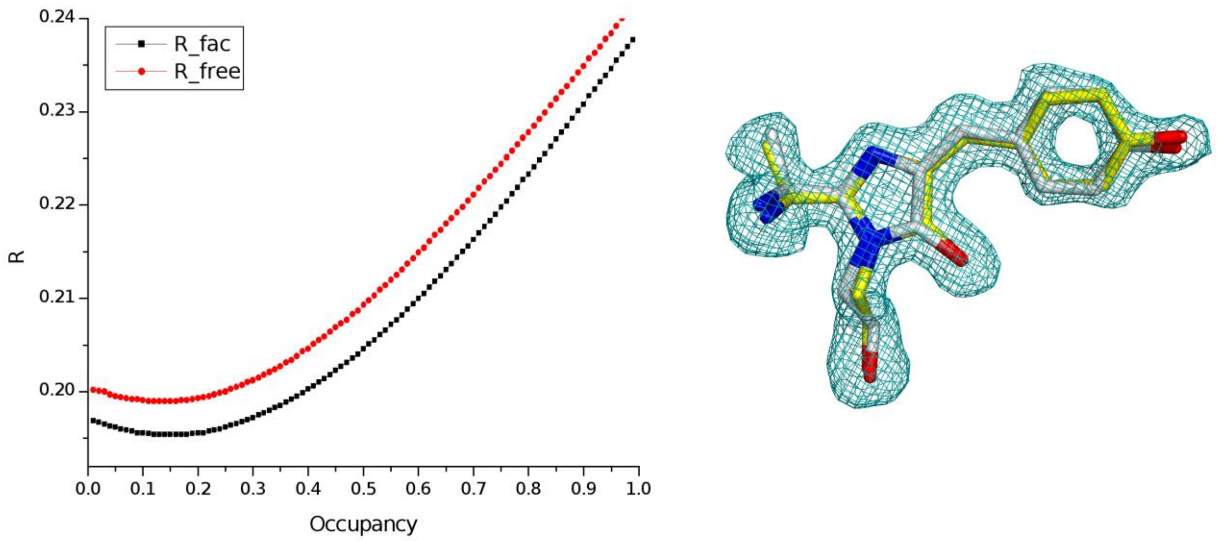

Supplementary Fig. 19 | LCLS LR23 PP 400 nm 550 fs. Left: R_{fac}/R_{free} calculation for varied occupancy of refined coordinates. Occupancy refers to the proportion of extrapolated coordinates (light) compared to ground state coordinates (dark). Both R_{fac} and R_{free} minimize at dark = 0.86 and light = 0.14. Right: Chromophore omitted F_o-F_c density contoured at +3σ level (teal) for 0.86:0.14 (ground:light) occupancy model. The coordinates refined to an extrapolated value of N_{EXT} = 8.6 are shown in grey (light) and ground state in yellow. R-free was calculated using 10% reflections

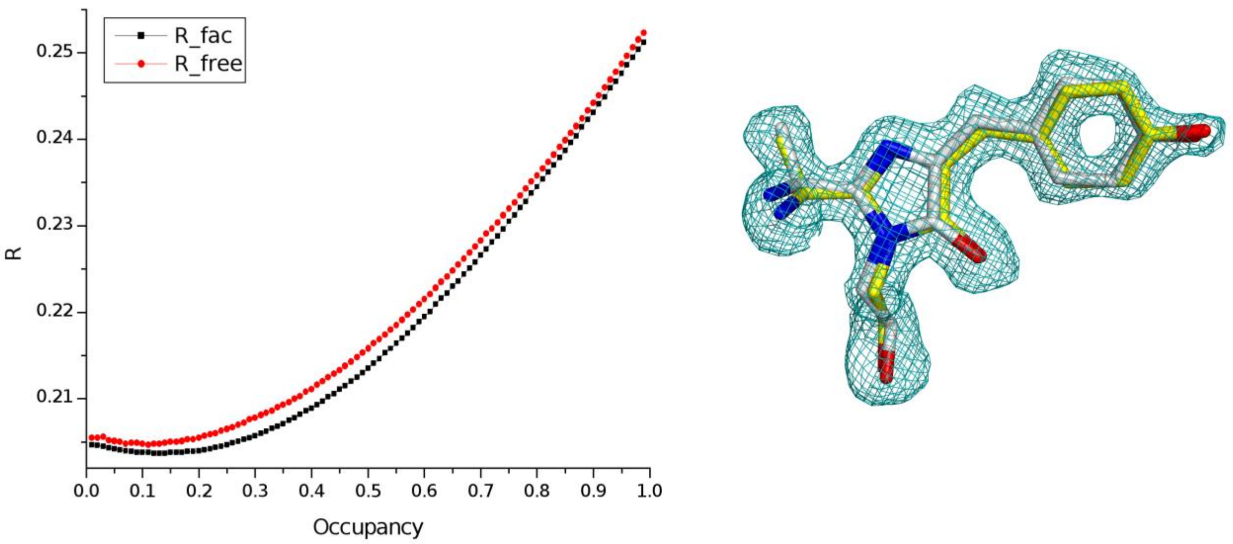

Supplementary Fig. 20 | LCLS LR23 PP 400 nm 700 fs. Left: R_{fac}/R_{free} calculation for varied occupancy of refined coordinates. Occupancy refers to the proportion of extrapolated coordinates (light) compared to ground state coordinates (dark). Both R_{fac} and R_{free} minimize at dark = 0.87 and light = 0.13. Right: Chromophore omitted F_o-F_c density contoured at +3σ level (teal) for 0.87:0.13 (ground:light) occupancy model. The coordinates refined to an extrapolated value of N_{EXT} = 8.6 are shown in grey (light) and ground state in yellow.

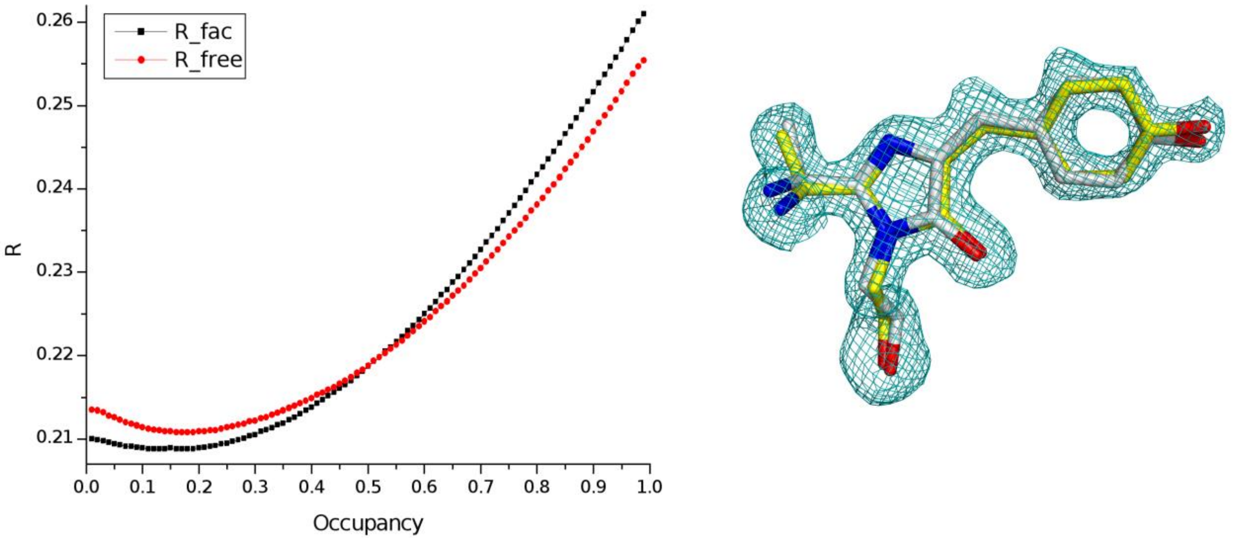

Supplementary Fig. 21 | LCLS LR23 PP 400 nm 850 fs. Left: R_{fac}/R_{free} calculation for varied occupancy of refined coordinates. Occupancy refers to the proportion of extrapolated coordinates (light) compared to ground state coordinates (dark). Both R_{fac} and R_{free} minimize at dark = 0.88 and light = 0.12. Right: Chromophore omitted F_o-F_c density contoured at +3σ level (teal) for 0.88:0.12 (ground:light) occupancy model. The coordinates refined to an extrapolated value of N_{EXT} = 8.6 are shown in grey (light) and ground state in yellow.

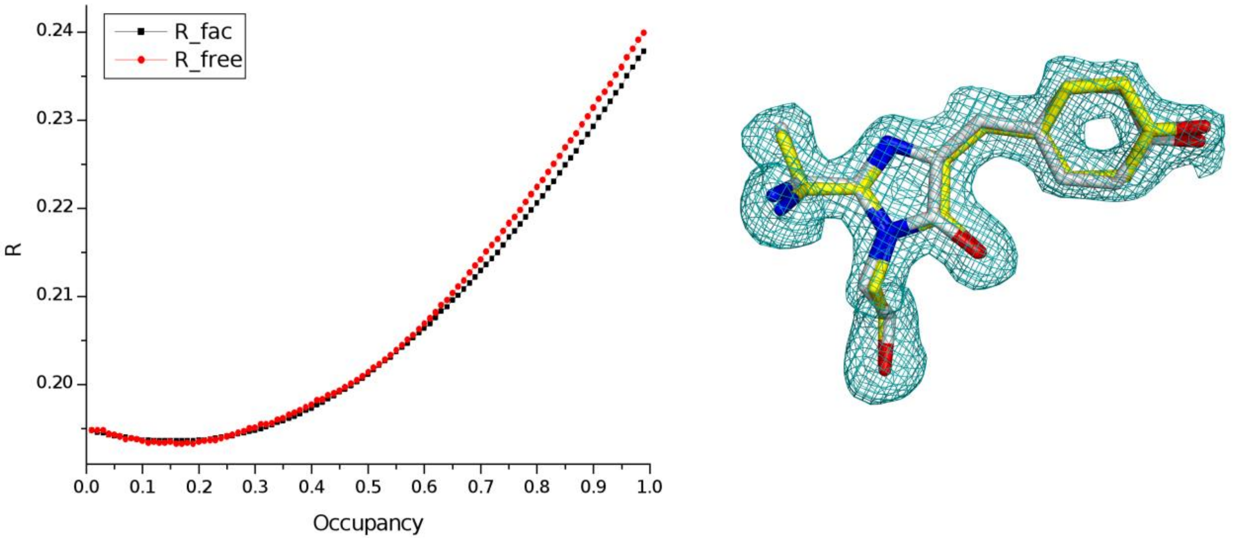

Supplementary Fig. 22 | LCLS LR23 PP 400 nm 1100 fs. Left: R_{fac}/R_{free} calculation for varied occupancy of refined coordinates. Occupancy refers to the proportion of extrapolated coordinates (light) compared to ground state coordinates (dark). Both R_{fac} and R_{free} minimize at dark = 0.83 and light = 0.17. Right: Chromophore omitted F_o-F_c density contoured at +3σ level (teal) for 0.83:0.17 (ground:light) occupancy model. The coordinates refined to an extrapolated value of N_{EXT} = 8.6 are shown in grey (light) and ground state in yellow.

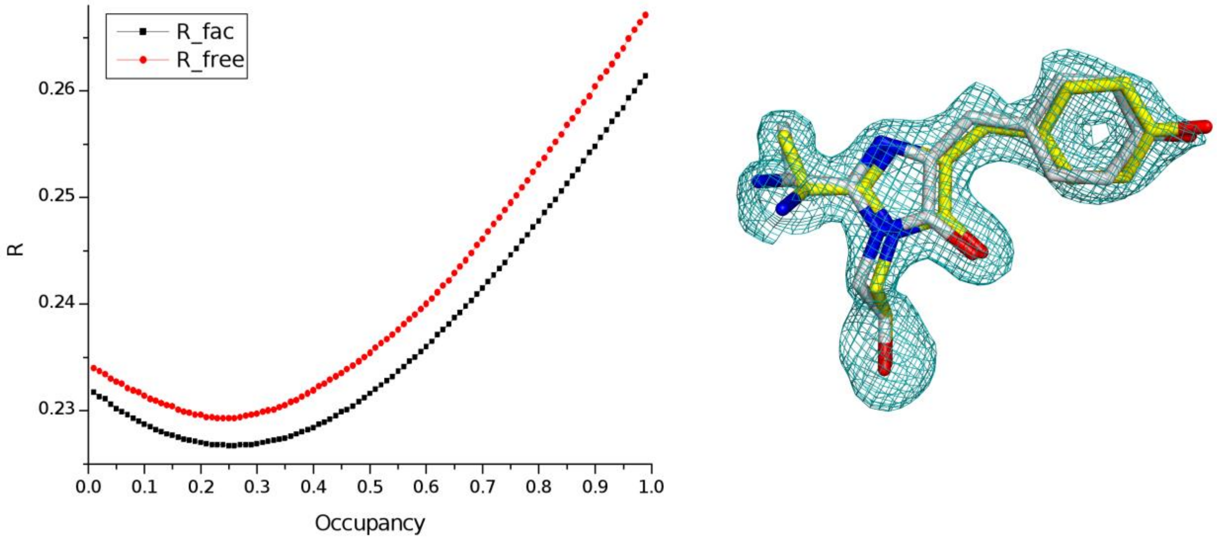

Supplementary Fig. 23 | LCLS LR23 PDP 400-515 nm 250 fs. Left: R_{fac}/R_{free} calculation for varied occupancy of refined coordinates. Occupancy refers to the proportion of extrapolated coordinates (light) compared to ground state coordinates (dark). Both R_{fac} and R_{free} minimize at dark = 0.75 and light = 0.25. Right: Chromophore omitted F_o-F_c density contoured at +3σ level (teal) for 0.75:0.25 (ground:light) occupancy model. The coordinates refined to an extrapolated value of N_{EXT} = 5.6 are shown in grey (light) and ground state in yellow.

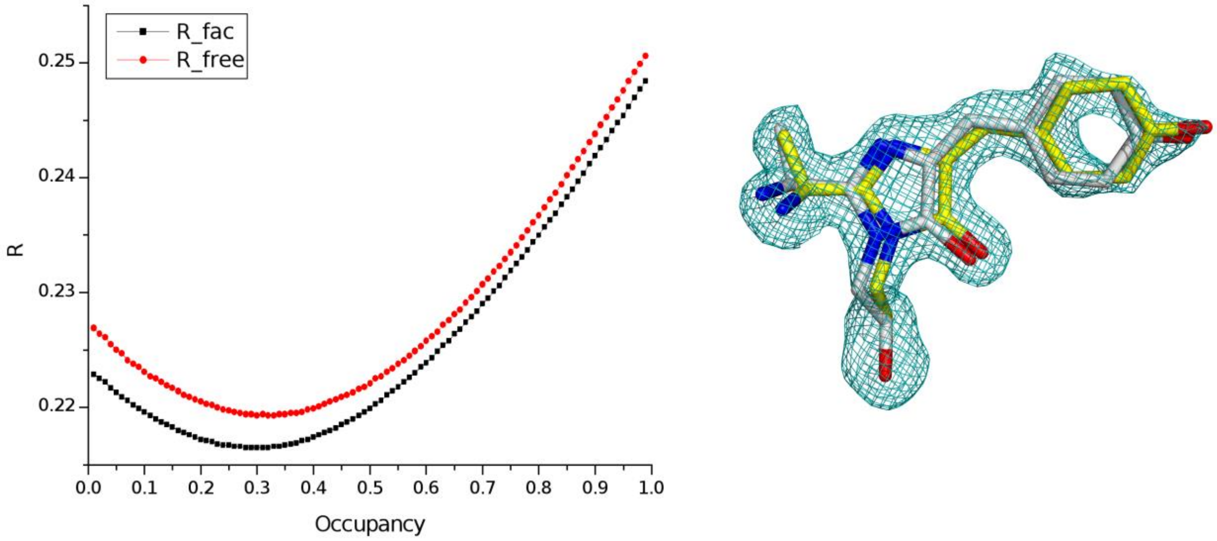

Supplementary Fig. 24 | LCLS LR23 PDP 400-515 nm 400 fs. Left: R_{fac}/R_{free} calculation for varied occupancy of refined coordinates. Occupancy refers to the proportion of extrapolated coordinates (light) compared to ground state coordinates (dark). Both R_{fac} and R_{free} minimize at dark = 0.70 and light = 0.30. Right: Chromophore omitted F_o-F_c density contoured at +3σ level (teal) for 0.70:0.30 (ground:light) occupancy model. The coordinates refined to an extrapolated value of N_{EXT} = 5.6 are shown in grey (light) and ground state in yellow.

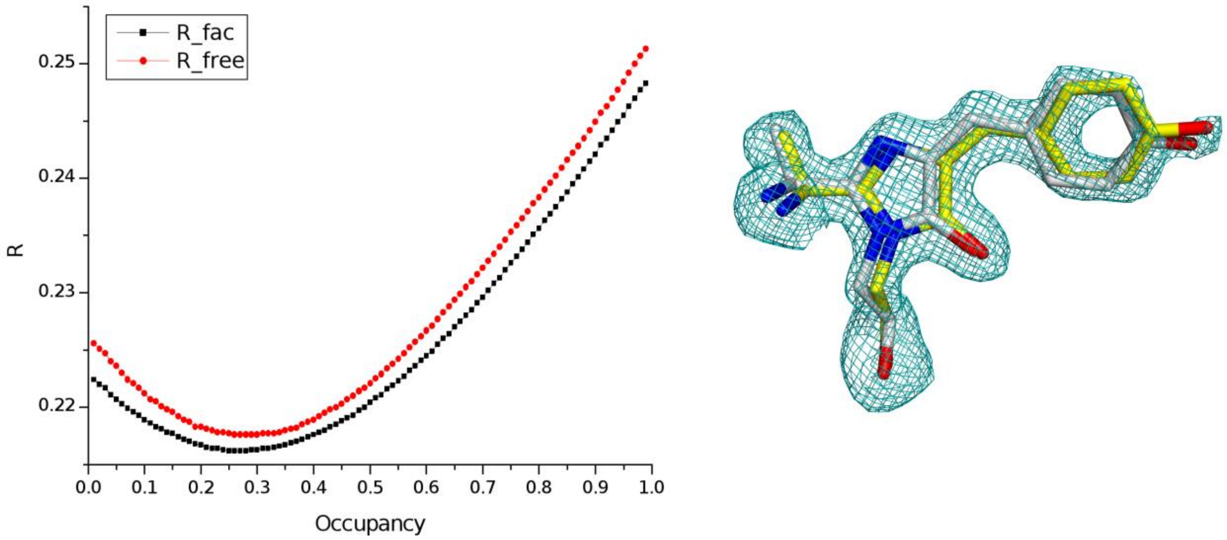

Supplementary Fig. 25 | LCLS LR23 PDP 400-515 nm 550 fs. Left: R_{fac}/R_{free} calculation for varied occupancy of refined coordinates. Occupancy refers to the proportion of extrapolated coordinates (light) compared to ground state coordinates (dark). Both R_{fac} and R_{free} minimize at dark = 0.84 and light = 0.16. Right: Chromophore omitted F_o-F_c density contoured at +3σ level (teal) for 0.84:0.16 (ground:light) occupancy model. The coordinates refined to an extrapolated value of N_{EXT} = 5.6 are shown in grey (light) and ground state in yellow.

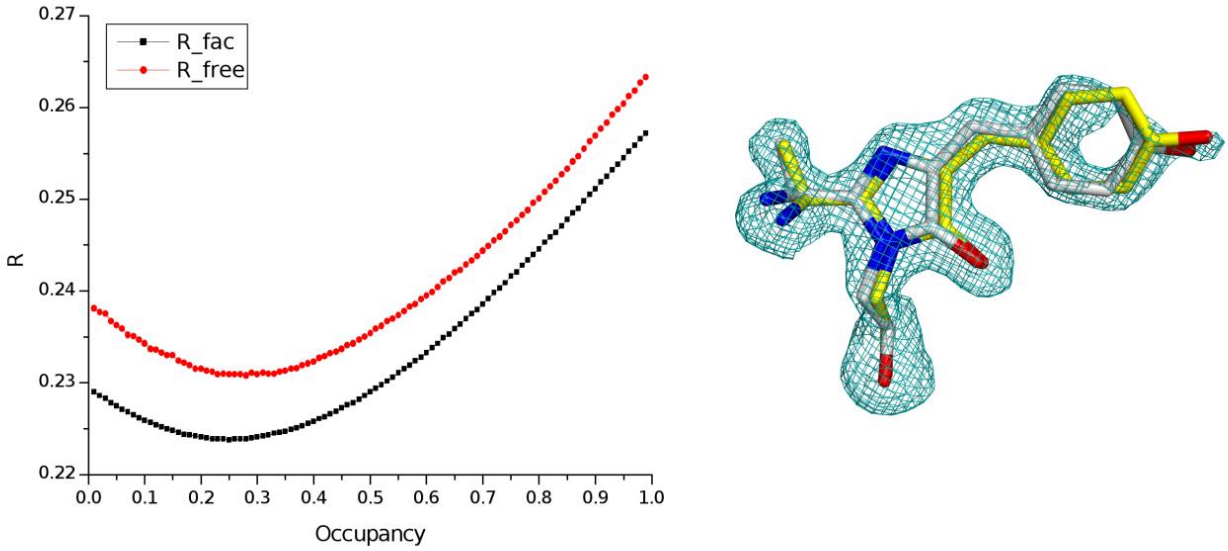

Supplementary Fig. 26 | LCLS LR23 PDP 400-515 nm 700 fs. Left: R_{fac}/R_{free} calculation for varied occupancy of refined coordinates. Occupancy refers to the proportion of extrapolated coordinates (light) compared to ground state coordinates (dark). Both R_{fac} and R_{free} minimize at dark = 0.75 and light = 0.25. Right: Chromophore omitted F_o-F_c density contoured at +3σ level (teal) for 0.75:0.25 (ground:light) occupancy model. The coordinates refined to an extrapolated value of N_{EXT} = 5.6 are shown in grey (light) and ground state in yellow.

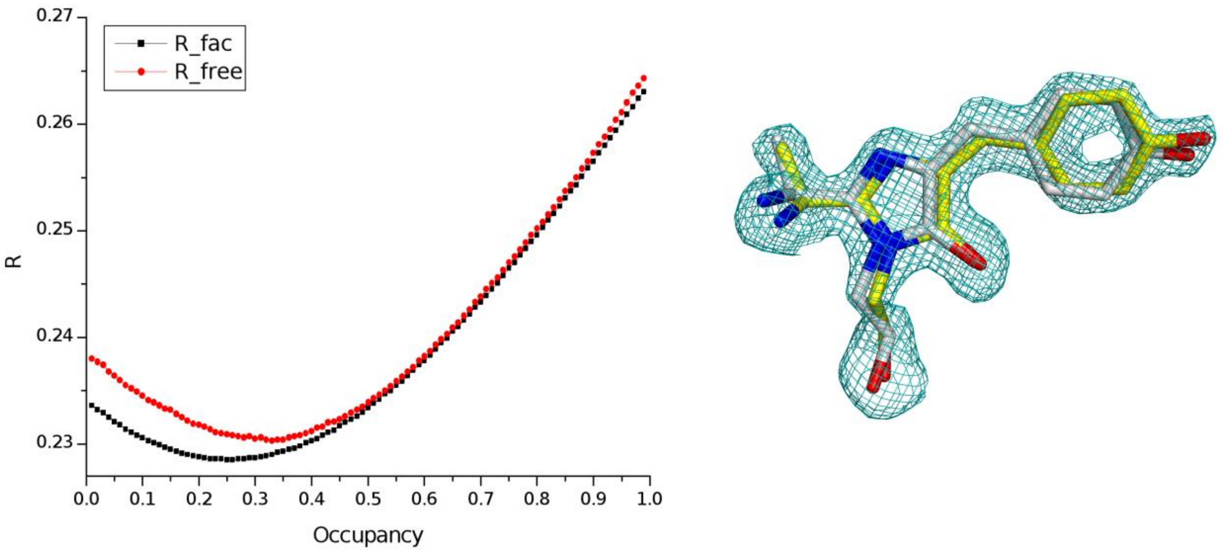

Supplementary Fig. 27 | LCLS LR23 PDP 400-515 nm 850 fs. Left: R_{fac}/R_{free} calculation for varied occupancy of refined coordinates. Occupancy refers to the proportion of extrapolated coordinates (light) compared to ground state coordinates (dark). Both R_{fac} and R_{free} minimise at dark = 0.75 and light = 0.25. Right: Chromophore omitted F_o-F_c density contoured at +3σ level (teal) for 0.75:0.25 (ground:light) occupancy model. The coordinates refined to an extrapolated value of N_{EXT} = 5.6 are shown in grey (light) and ground state in yellow.

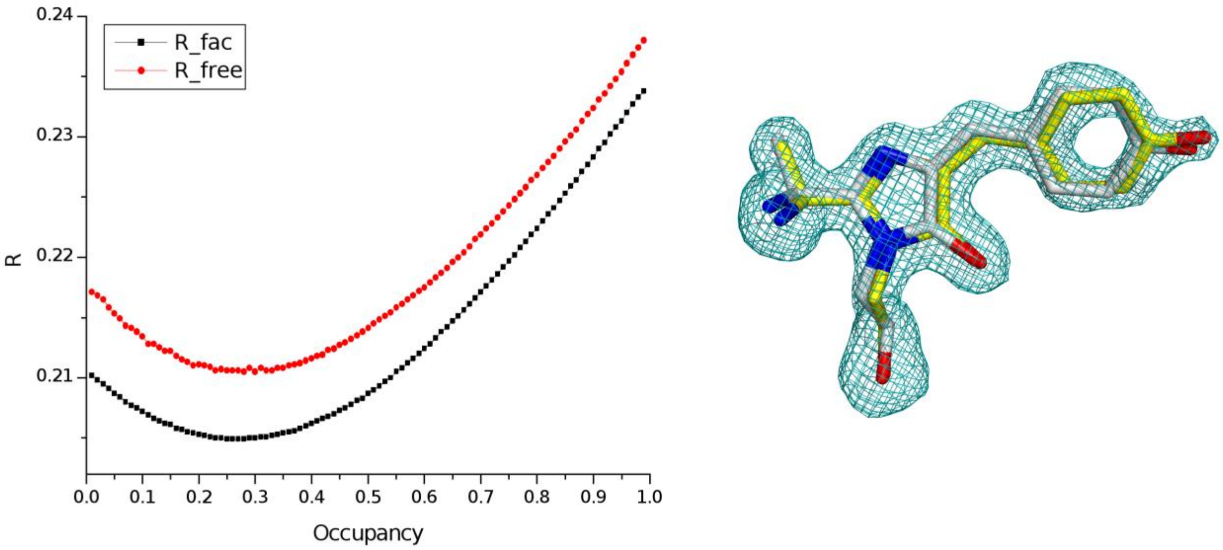

Supplementary Fig. 28 | LCLS LR23 PDP 400-515 nm 1100 fs. Left: R_{fac}/R_{free} calculation for varied occupancy of refined coordinates. Occupancy refers to the proportion of extrapolated coordinates (light) compared to ground state coordinates (dark). Both R_{fac} and R_{free} minimize at dark = 0.74 and light = 0.26. Right: Chromophore omitted F_o-F_c density contoured at +3σ level (teal) for 0.74:0.26 (ground:light) occupancy model. The coordinates refined to an extrapolated value of N_{EXT} = 5.6 are shown in grey (light) and ground state in yellow.

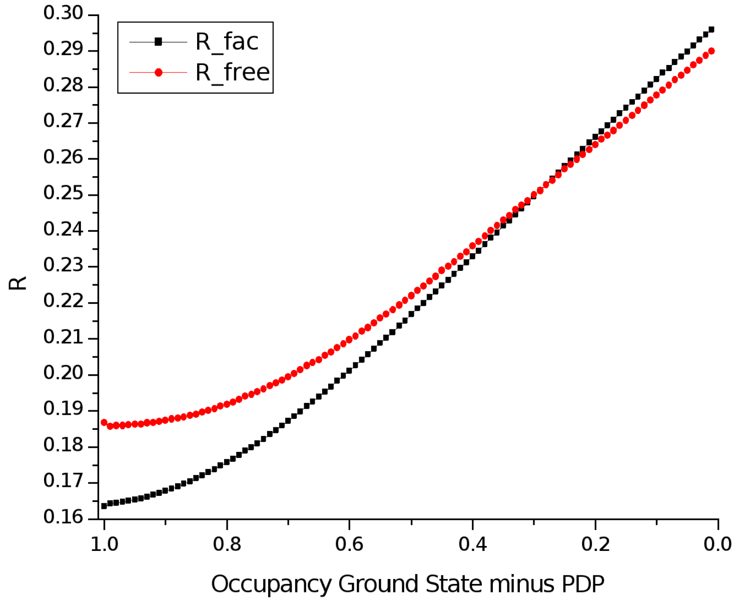

Supplementary Fig. 29 | LCLS LR23 Dark Occupancy Determination. Using Dark, Laser OFF data only (Extended Data Table 2). The R-factors were analysed with a resolution limit of 1.5 Å. The additional occupancy of PDP coordinates degrades the R-factor and R-free factor.

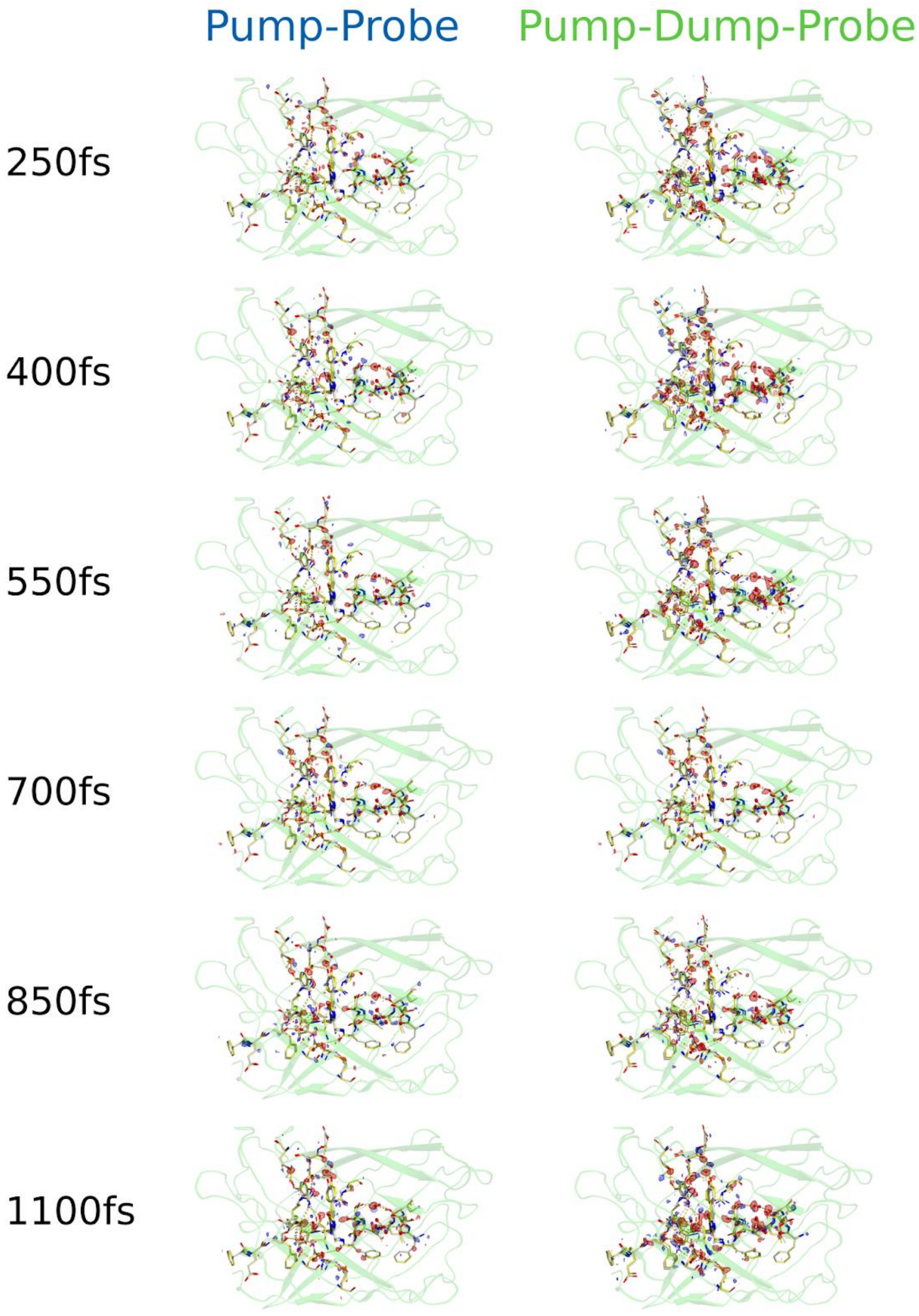

Supplementary Fig. 30 | LCLS LR23 TR-SFX PP vs PDP. Q-weighted difference electron density for each bin of the pump probe (400 nm) on the left and of the pump-dump probe (400-515 nm) on the right. The secondary structure (green) of the ground state coordinates (yellow) and PP (grey) and PDP (grey) coordinates are shown with electron density contoured at +3σ rms (blue) and -3σ rms (red).

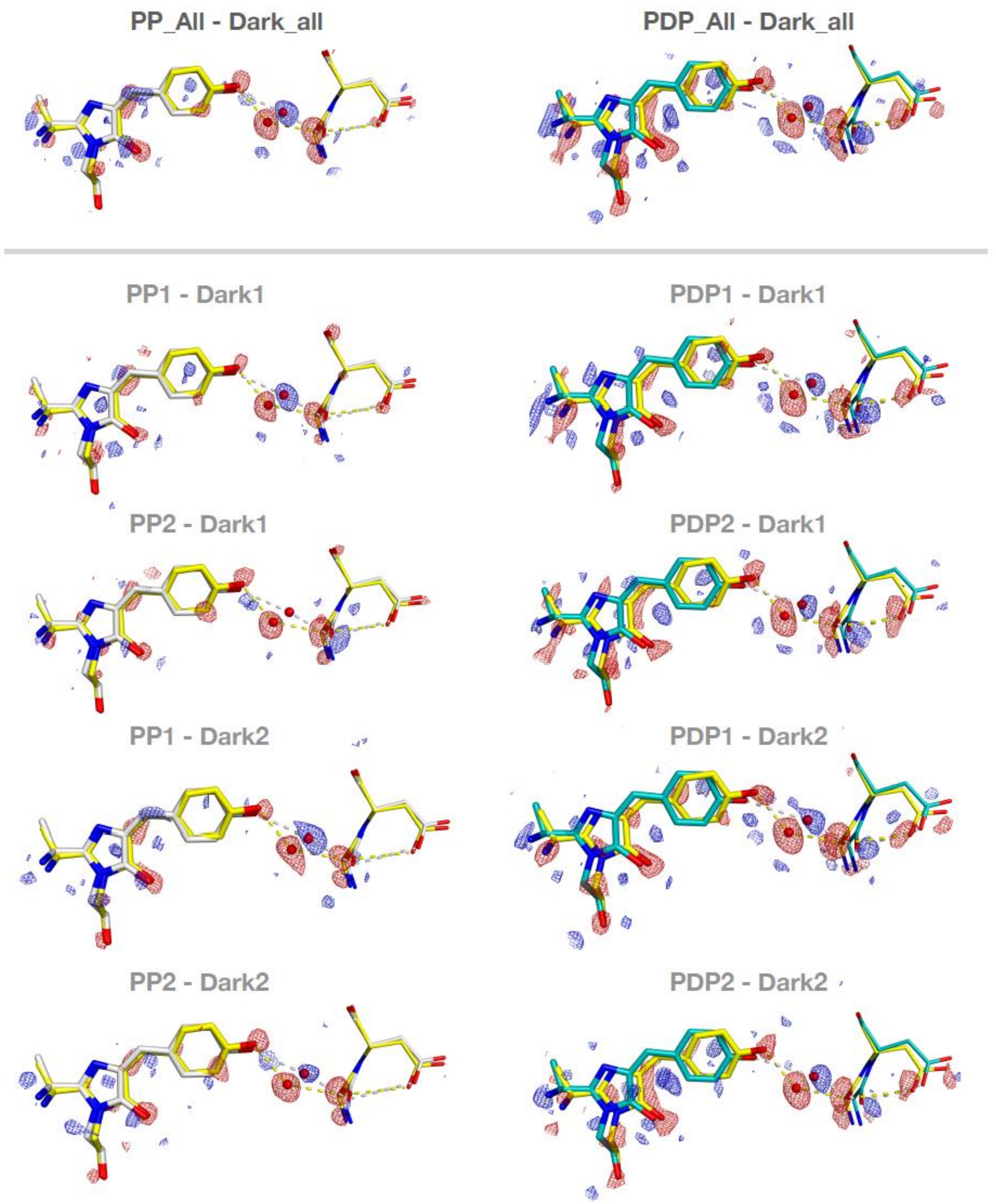

Supplementary Fig. 31 | LCLS LR23 PP vs PDP split data comparison. Femtosecond time resolved Pump-Probe (PP: left) and Pump-Dump-Probe (PDP: right) TR-SFX experiment of rsKiiro off state with a trans neutral chromophore. In the top row, Q-weighted Fo-Fo maps are shown for all of the collected PP, PDP, and dark data (as in Figure 2 of the main text). For the rows below, all datasets were split in two to demonstrate the reproducibility of the signals.

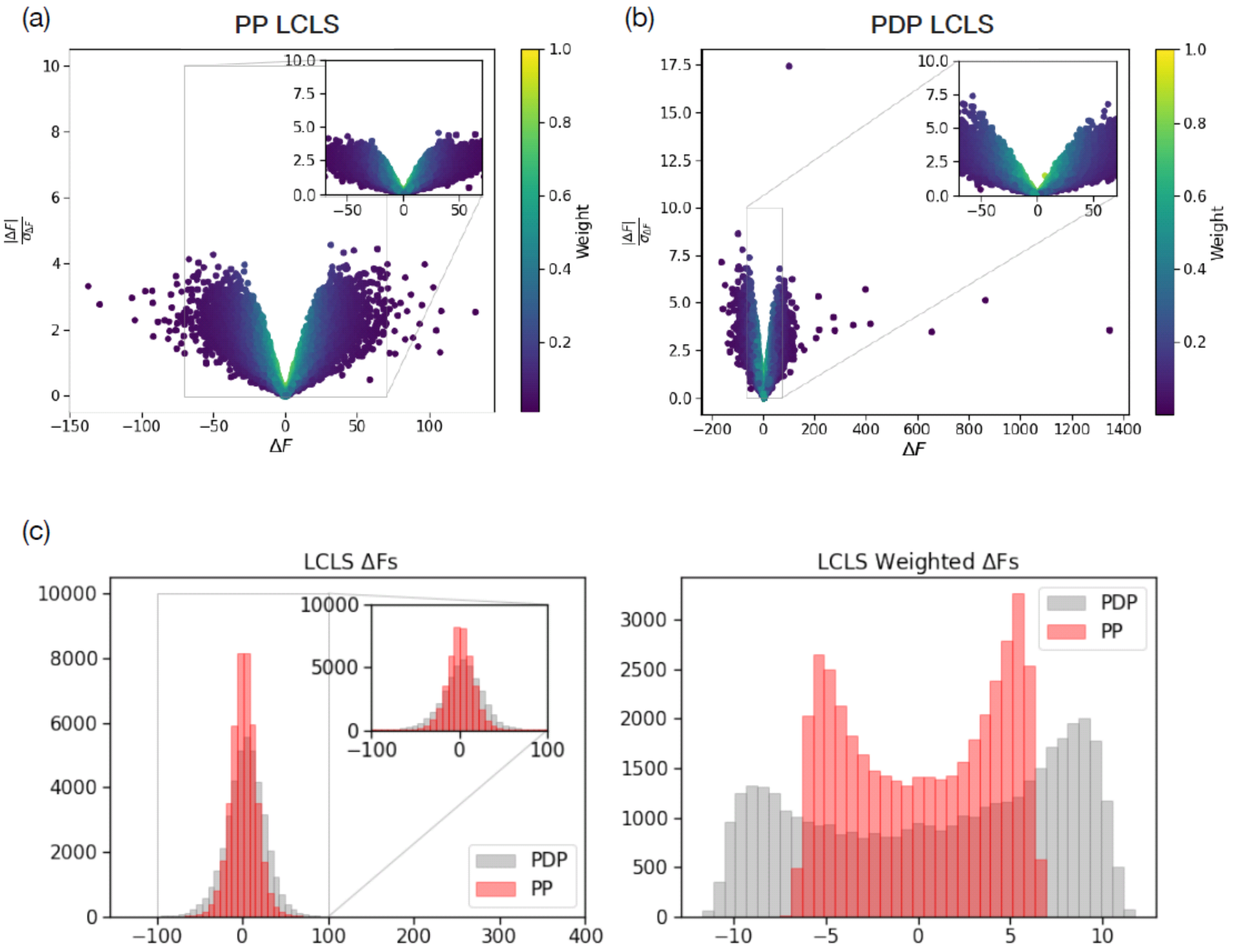

Supplementary Fig. 32 | LCLS LR23 Difference Structure Factors. Difference structure factor amplitudes for the femtosecond time resolved Pump-Probe (a) and Pump-Dump-Probe (b) LCLS TR-SFX experiment of rsKiiro are plotted, along with the weight assigned to each difference by the Q-weighting scheme. In (c), the distributions of difference structure factors amplitudes before and after Q-weighting are shown. Larger amplitudes are observable for the PDP dataset. Structure factors were plotted using the Reciprocal Spaceship package.

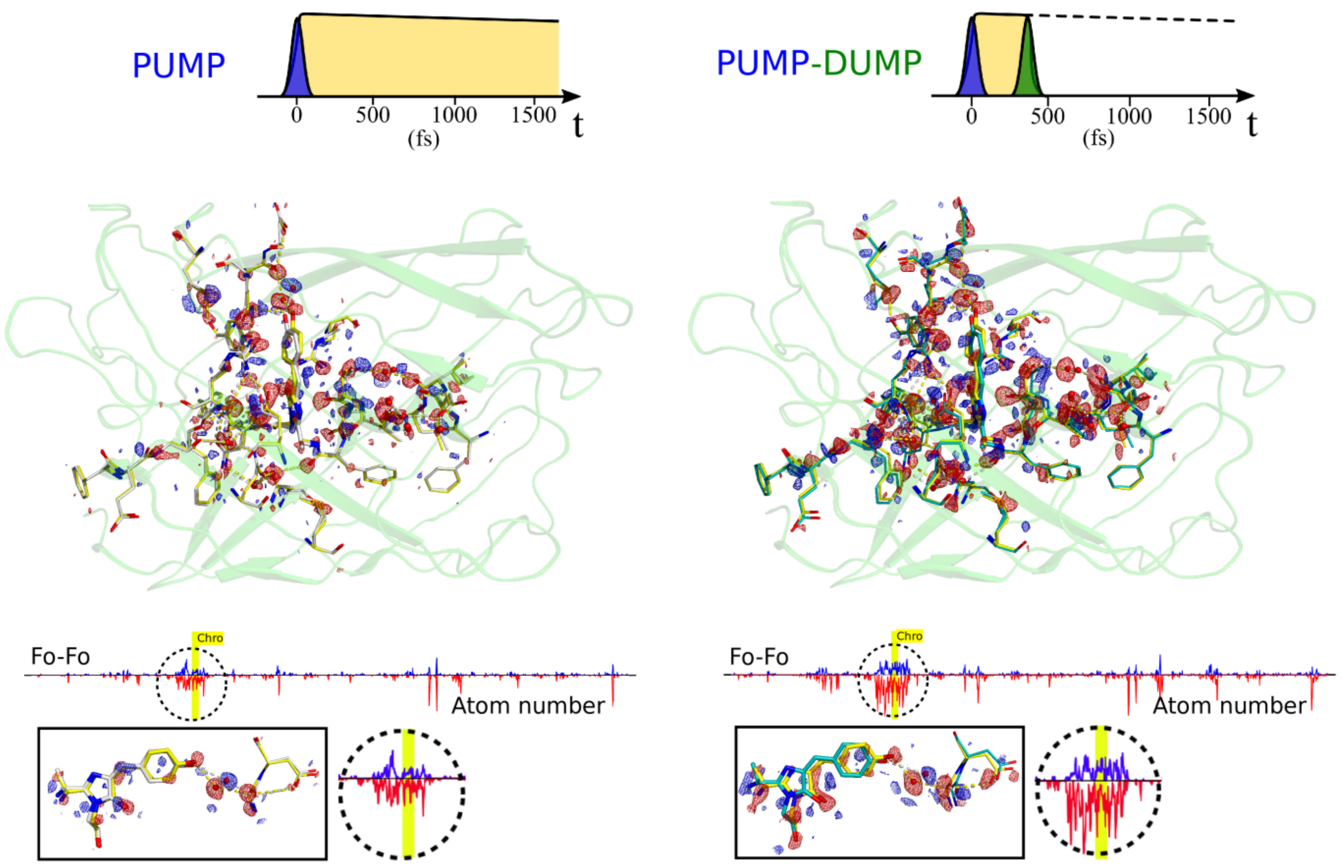

Supplementary Fig. 33 | LCLS LR23 Full protein overview of the PP and PDP electron density differences shown in Figure 2. The Fo-Fo difference maps are contoured at 3 sigma, as in Figure 2. The zoomed view of the integration of the electron density differences in the chromophore region emphasizes the amplification of the PDP data relative to the PP data.

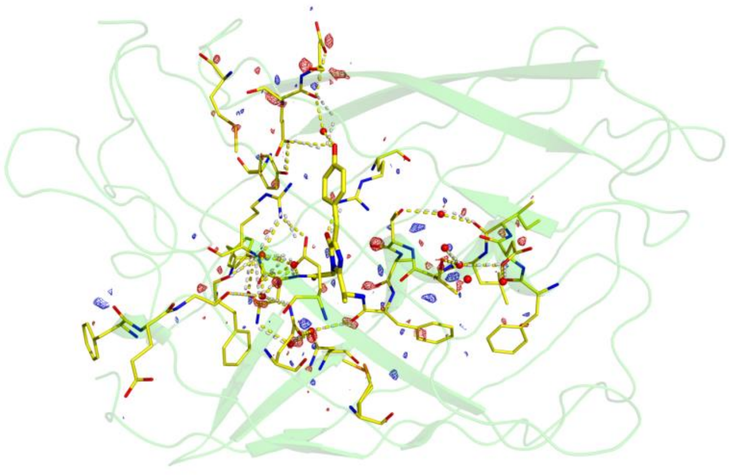

Supplementary Fig. 34 | LCLS LR23 Non-resonant pumping control. Fobs-Fobs Q weighted difference map for non-resonant 515nm pulse only, for LCLS experiment LR23. The data is for all positive sub-picosecond Dump-Probe delays. The map is contoured at 3 rms level. The difference data is calculated from merging 15,949 indexed Laser-ON diffraction images and 35,030 reference dark, indexed Laser-OFF diffraction images. Crystallographic statistics are shown in Supplementary Table. 3.

Supplementary Table. 3 | LCLS LR23 Dump only crystallographic table.

Dataset	Nonresonant-515nm-1ps
Wavelength (eV)	9,097.5
Resolution (Å)	31.94–1.8(1.90-1.80)[1]<br>31.94–1.5(1.58-1.53)[2]<br>31.94–1.32(1.38-1.33)[3]
Space group	$P2_12_12_1$
Unit cell (Å)	39.36 74.06 78.68<br>90 90 90
No. of patterns	23,216
No. of merged patterns	15,949
Total reflections	3,487,139[1]<br>4,113,940[2]<br>4,251,050[3]
Unique reflections	56,742[3]
Completeness	100.0[1]
Mean I/σ(I)	6.088(3.43)[1]<br>4.463(1.39)[2]<br>3.476(0.04)[3]
Wilson B-factor ($Å^{-2}$)(sigma)	13.390(0.466)
R-split	14.19(28.45)[1]<br>15.96(74.02)[2]<br>17.13(274.98)[3]
CC* correlation	0.9924(0.9620)[1]<br>0.9930(0.8398)[2]<br>0.9932(0.3243)[3]
$CC_{1/2}$ correlation	0.9701(0.8613)[1]<br>0.9725(0.5446)[2]<br>0.9732(0.0554)[3]

[1,2,3] Represent merged statistics for distinct resolution shells

5.2. SACLA 2017B8008

Additional experiments were aimed at evaluating the high peak power used in the TR-SFX experiments. Considering the high peak power of the Stokes field, TA spectroscopy measurements provide confidence on the resonant nature with stimulated emission that is highly selective, and also does not create detectable transient absorption in the absence of the pump interaction (Extended Data Fig.3h). However we addressed potential concern regarding non-resonant 3-photon ionization of water[11] that has been shown to occur already at an excitation of 100 GW cm^{-2} . Negative control experiments at LCLS already confirmed an absence of photoinduced signals for Stokes-only conditions at an average ~500 fs delay time (Supplementary Fig. 34) In addition, we collected 71,055 diffraction patterns with Stokes-only pumping (4 mJ/mm^2) and a 0-1 ps delay and 101,378 dark frames at 1.5 Å resolution at SACLA, which could be processed and scaled with excellent statistics (Supplementary Table. 4). Also for this data set, refinement did not reveal Q-weighted electron density differences due to the interaction with the Stokes field, in spite of its high intensity, the pressure differential, and possible 3-photon water ionization background reactions.

A non-resonant PP control experiment was conducted using only 515 nm illumination pulses on the preilluminated off state. Q-weight difference map between merged 0-1 ps laser on and merged laser off, dark data (Supplementary Fig. 35) shows no significant light induced differences consistent with TA results (Extended Data Fig. 3h). Crystallographic statistics are shown in Supplementary Table. 4.

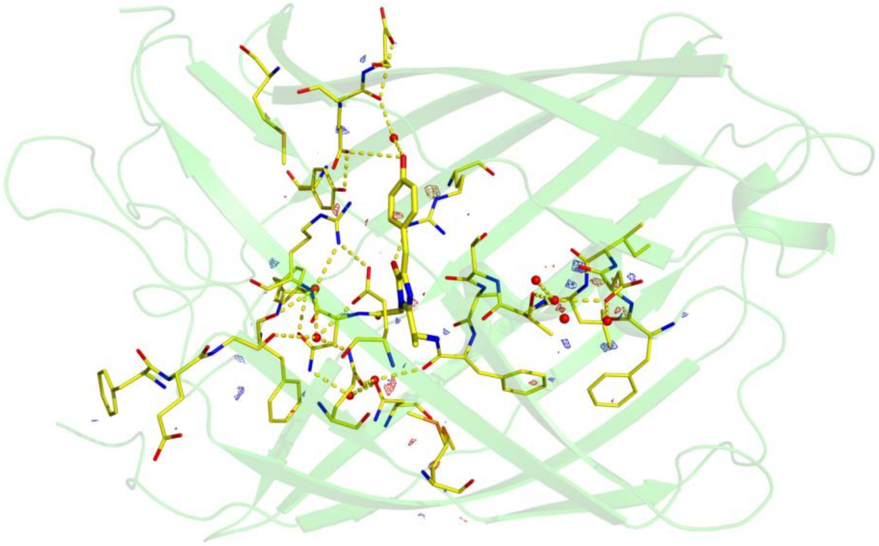

Supplementary Fig. 35 | SACLA 2017B8008 non-resonant pumping. Negative control map showing the Q-weighted difference electron density between the non-resonant, 515 nm 0-1 ps pumping of the off state and merged laser-off, dark data. The secondary structure (green) of the ground state coordinates (yellow) are shown with electron density contoured at +3σ rms (blue) and -3σ rms (red). The experiment used merging of 101,387 dark frames and 71,055 pumped frames with a resolution extending to 1.35Å and excellent statistics (Supplementary Table. 4).

Supplementary Table. 4 | SACLA 2017B8008 crystallographic statistics

	Dark	515nm 0-1ps
Indexed Patterns:	101378	71055
Resolution Limits (Å)	31.77-1.80 (1.864-1.800)[1]	31.77-1.80 (1.864-1.800)[1]
	31.77-1.50 (1.554-1.500)[2]	31.77-1.50 (1.554-1.500)[2]
	31.77-1.43 (1.398-1.350)[3]	31.77-1.43 (1.398-1.350)[3]
Number of Unique reflection indices:	21985[1]	21985[1]
	37586[2]	37586[2]
	51273[3]	51273[3]
No. Merged Reflections:	18598709 (1011010)[1]	13424238 (730782)[1]
	21443852 (114082)[2]	15442639 (77620)[2]
	21475550 (0)[3]	15462354 (0)[3]
Completeness (%):	100.00 (100.00)[1]	100.00 (100.00)[1]
	99.52 (95.32)[2]	98.92 (89.62)[2]
	79.11 (0.00)[3]	77.18 (0.00)[3]
Signal to noise:	15.995 (7.51)[1]	13.262 (5.91)[1]
	10.524 (1.02)[2]	8.709 (0.86)[2]
	9.768 (N/A)[3]	8.248 (N/A)[3]
Wilson b factor:	20.83[1]	21.11[1]
	24.54[2]	25.09[2]
	23.51[3]	24.66[3]
R_{Split} (%):	5.66 (13.62)[1]	6.64 (17.52)[1]
	6.75 (152.39)[2]	8.06 (250.45)[2]
	6.96 (N/A)[3]	8.28 (N/A)[3]
CC*:	1.00 (0.99)[1]	1.00 (0.99)[1]
	1.00 (0.56)[2]	1.00 (0.36)[2]
	1.00 (N/A)[3]	1.00 (N/A)[3]
$CC_{1/2}$:	0.99 (0.97)[1]	0.99 (0.96)[1]
	0.99 (0.19)[2]	0.99 (0.07)[2]
	0.99 (N/A)[3]	0.99(N/A)[3]

[1,2,3] Represent merged statistics for distinct resolution shells N/A is used for when statistical measures for the inner shell where unphysical.

5.3. SACLA 2019B8021

Figure 3 shows data from experiments collected during SACLA 2019B8021. This was a repeat of LR23 experiment as well as measuring longer PDP delay of 2 ps. PP and PDP datasets were collected. FSC and R-split for merged laser off and dark data are shown in Supplementary Fig. 36. All sub-ps data was merged in a single dataset with an average delay of 500fs for both PP and PDP. N_{EXT} determination is shown in Supplementary Fig. 37. The PP (Supplementary Fig. 38) signals show a decay of the light induced difference after 3ps. Sub-picosecond PP and PDP (Supplementary Fig. 39) show the same increase signals due to the presence of the dump pulse and in the same locations as those from LCLS (Supplementary Fig. 40). The PDP shows a moderate decay of the electron density signals after 3ps and 100ps (Supplementary Fig. 41). Negative time control maps are shown in Supplementary Fig. 42. Crystallographic statistics for the PP and PDP are shown in Supplementary Table. 5.

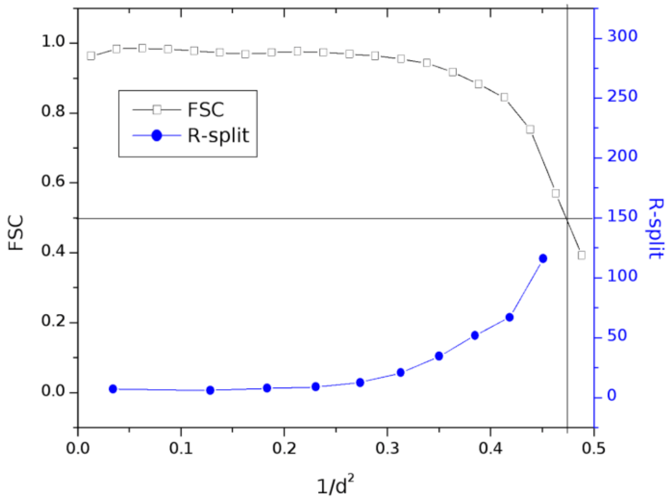

Supplementary Fig. 36 | SACLA 2019B8021 FSC and R-Split for the all merged laser off, dark data. An FSC of 0.5 is chosen for a limiting 1.44 Å.

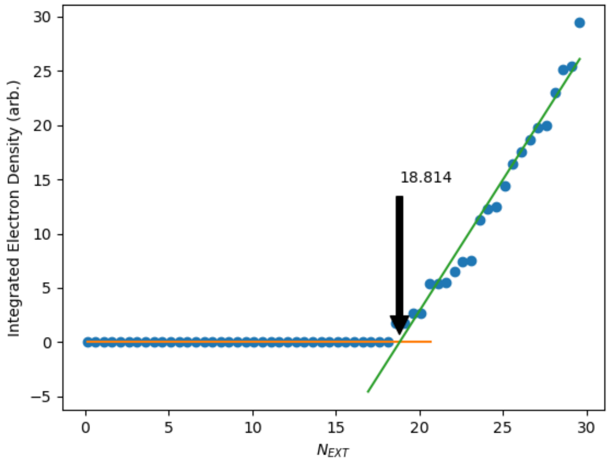

Supplementary Fig. 37 | SACLA 2019B8021 occupancy refinement. Pump Probe <1 ps (left) and Pump-Dump Probe <1 ps (right): Extrapolated factor, N_{EXT}, determination through the intersection point of two linear fits to the magnitude of integrated negative electron density, shown as blue circles.

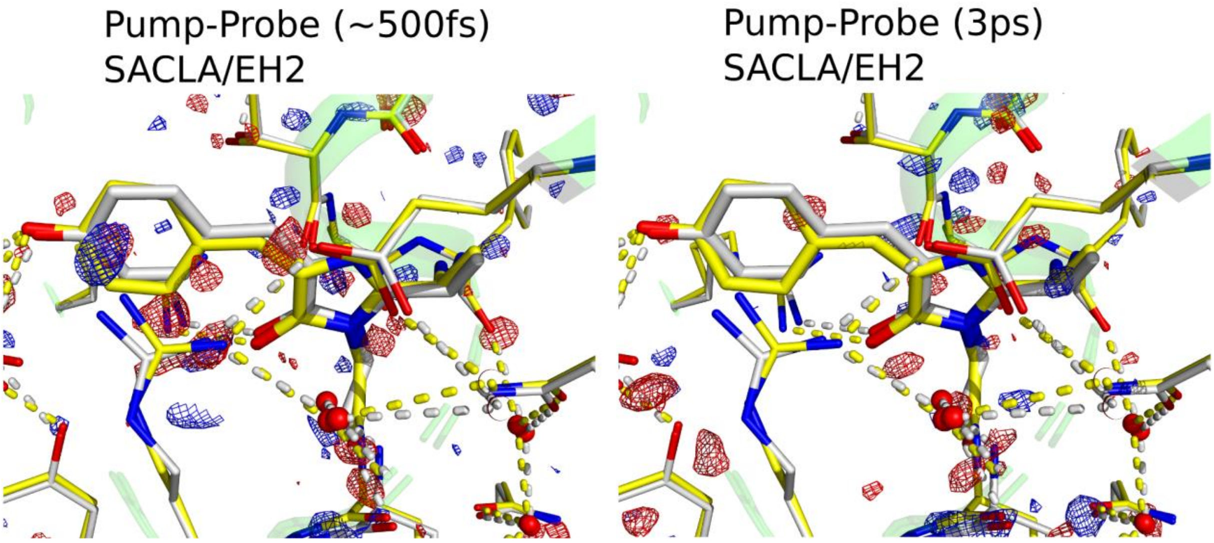

Supplementary Fig. 38 | SACLA 2019B8021 TR-SFX PP difference maps. A comparison of the average ~500 fs delay and 3 ps delay pump-probe data shows a clear decay of Q-weighted difference density features. The secondary structure (green) of the ground state coordinates (yellow) are shown with electron density contoured at +3σ rms (blue) and -3σ rms (red).

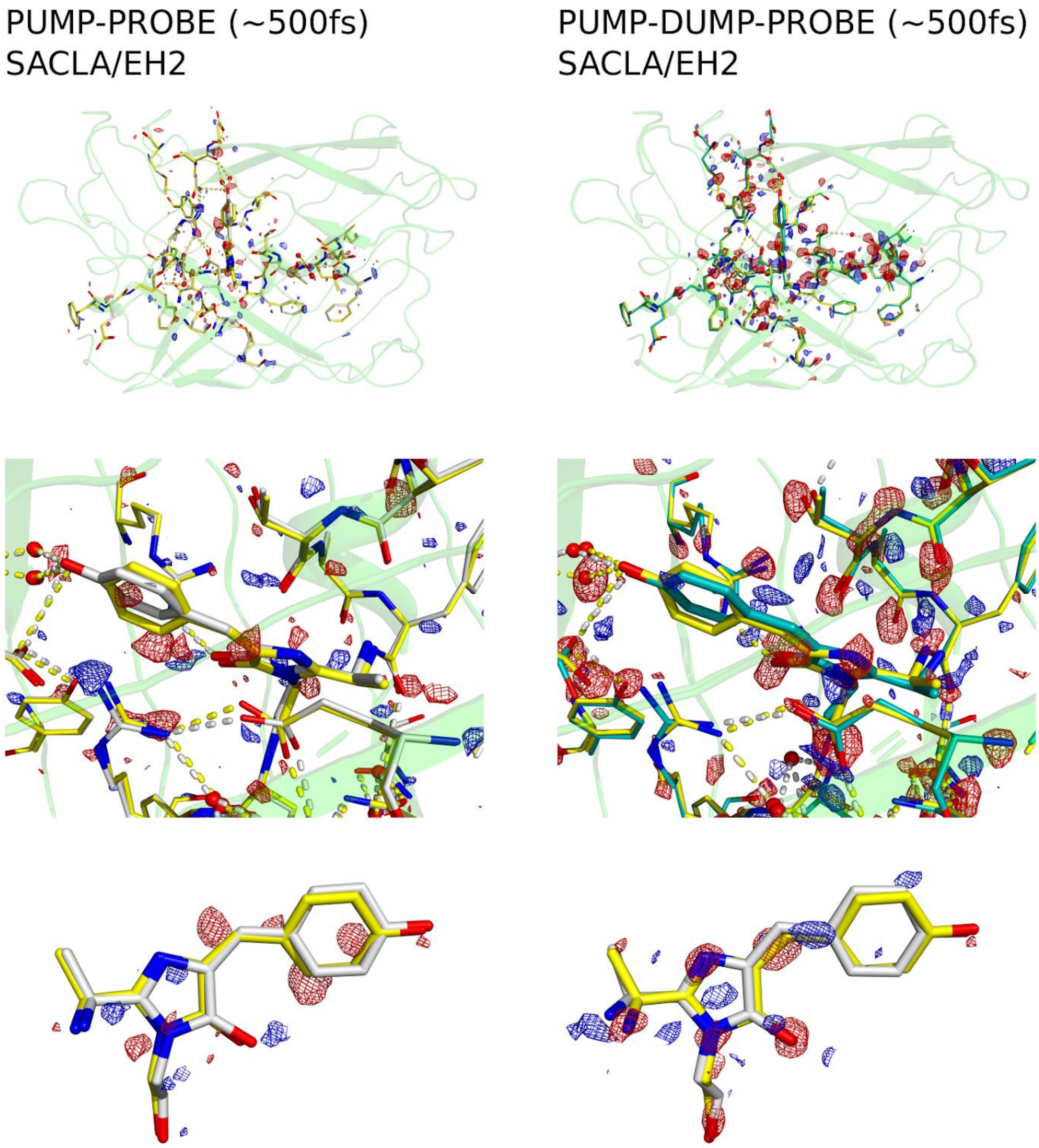

Supplementary Fig. 39 | SACLA 2019B8021 TR-SFX PP vs PDP difference maps. Q-weighted difference electron density, on the left: differences between the pump probe average delay 500 fs and dark (laser off). On the right: differences between the pump-dump probe average delay 500 fs and dark (laser off). The addition of the dump-pulse 350 fs after the pump, increases the electron density differences amplitudes, and larger shifts of the fitted coordinates to extrapolated structure factors (shown in grey and green), taken as N_{EXT} = 18.6 for the pump probe and N_{EXT} = 17.1 for pump-dump. The secondary structure (green) of the ground state coordinates (yellow) are shown with electron density contoured at +3σ rms (blue) and -3σ rms (red).

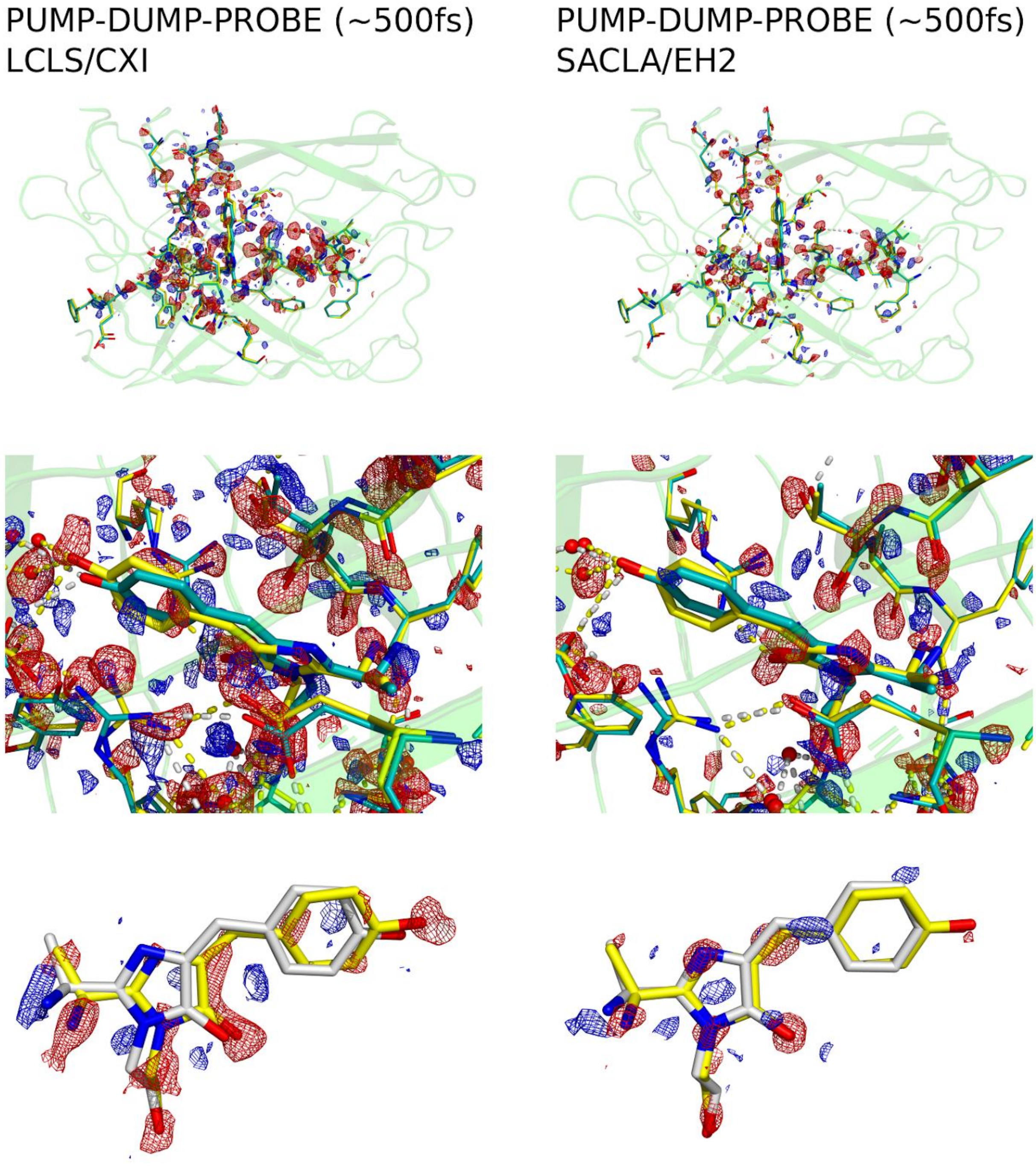

Supplementary Fig. 40 | LCLS LR23 PDP vs SACLA 2019B8021 PDP difference maps. Comparison of Q-weighted difference electron density differences of the pump-dump probe signals at LCLS/CXI and SACLA/EH2. Left: differences between the pump dump-probe 0-1 ps probe delay and dark (laser off). Right: differences between the pump-dump probe average probe delay 500 fs and dark (laser off). Extrapolated structures for each structure are shown in grey / teal, the secondary structure (green) of the ground state coordinates (yellow) are shown with electron density contoured at +3σ rms (blue) and -3σ rms (red).

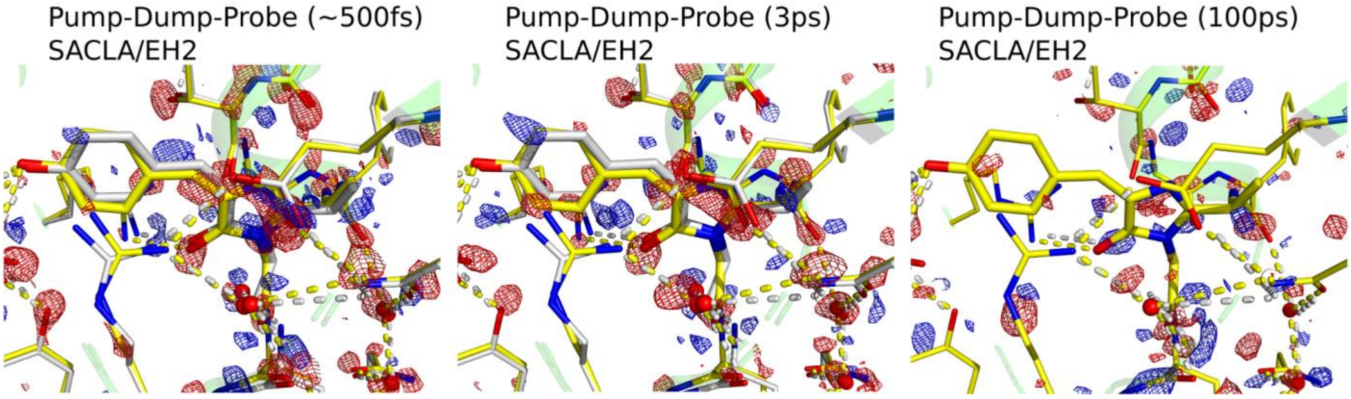

Supplementary Fig. 41 | SACLA 2019B8021 TR-SFX PDP difference maps. The comparison of the average ~500 fs delay, 3 ps and 100 ps delay pump-dump-probe data shows a moderate decay of the electron density signals from 500 fs to 3 ps, corresponding to the 4 ps decay time observed for the ground state intermediate induced absorption at 440 nm (Extended Data Fig. 3e). By 100 ps the signals are reduced to mostly noise. Extrapolated structures for each structure are shown in grey / teal, the secondary structure (green) of the ground state coordinates (yellow) are shown with electron density contoured at +3σ rms (blue) and -3σ rms (red).

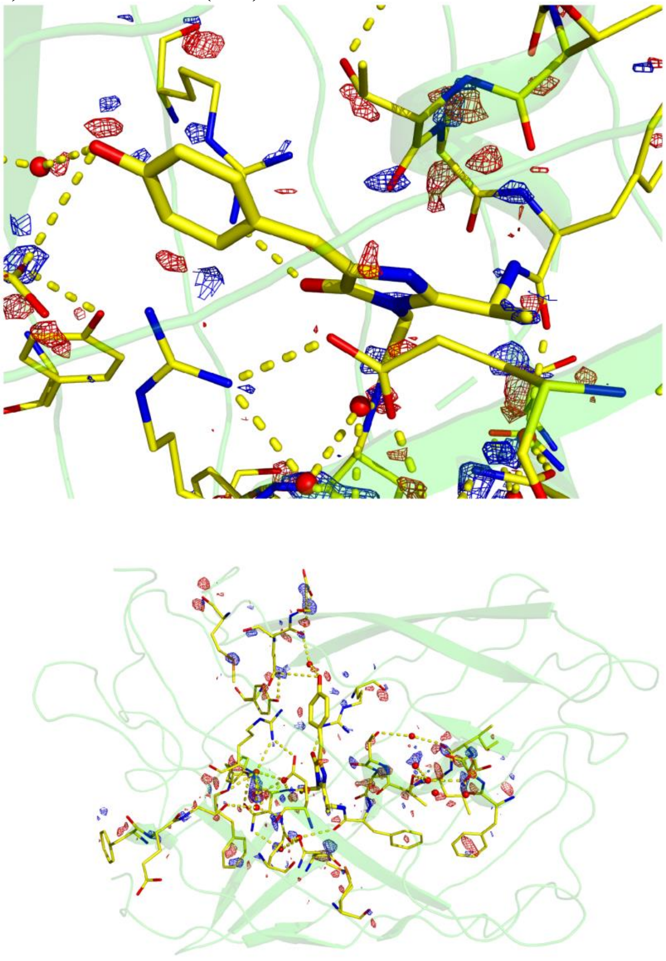

Supplementary Fig. 42 | SACLA2019B8021 TR-SFX PP Negative time control. Difference maps for minus 5 ps PP data with 400nm excitation. The X-ray probe arrives 5 ps before the 400nm laser pulse. Q-weighted electron density differences, contoured at +3σ rms (blue) and -3σ rms (red) are shown for the negative 5 ps laser on data, merging 17,627 frames, and the dark laser off data, merging 77,581 frames (Supplementary Table. 5). Coordinates are shown for the refined dark data.

Supplementary Table. 5 | SACLA 2019B8021 TR-SFX PP & PDP crystallographic statistics

	Dark	400nm 0 - 1 ps	400nm 3 ps	400nm 100 ps	400nm -5 ps	400nm-515nm 0 - 1 ps*	400nm-515nm 3 ps*	400nm-515nm 100 ps*	400nm-515nm 3 ps†
Indexed Patterns:	77581	18886	20472	18886	17627	54743	83187	25204	21796
Resolution Limits (Å)	18.84-1.70 (1.761-1.700)[1]	18.53-1.70 (1.761-1.700)[1]	18.53-1.70 (1.761-1.700)[1]	18.53-1.70 (1.761-1.700)[1]	18.53-1.70 (1.761-1.700)[1]	18.84-1.70 (1.761-1.700)[1]	18.84-1.70 (1.761-1.700)[1]	18.53-1.70 (1.761-1.700)[1]	18.53-1.70 (1.761-1.700)[1]
	18.84-1.65 (1.709-1.650)[2]	18.53-1.65 (1.709-1.650)[2]	18.53-1.65 (1.709-1.650)[2]	18.53-1.65 (1.709-1.650)[2]	18.53-1.65 (1.709-1.650)[2]	18.84-1.65 (1.709-1.650)[2]	18.84-1.65 (1.709-1.650)[2]	18.53-1.65 (1.709-1.650)[2]	18.53-1.65 (1.709-1.650)[2]
	18.84-1.46 1.491-1.440)[3]	18.53-1.50 (1.491-1.440)[3]	18.53-1.53 (1.491-1.440)[3]	18.53-1.53 (1.491-1.440)[3]	18.53-1.53 (1.491-1.440)[3]	18.84-1.46 (1.491-1.440)[3]	18.84-1.46 (1.491-1.440)[3]	18.53-1.55 (1.491-1.440)[3]	18.53-1.47 (1.491-1.440)[3]
Number of Unique reflection indices:	26206[1]	26206[1]	26205[1]	26205[1]	26206[1]	26206[1]	26209[1]	26206[1]	26205[1]
	28578[2]	28578[2]	28577[2]	28577[2]	28578[2]	28578[2]	28581[2]	28578[2]	28577[2]
	42704[3]	42704[3]	42703[3]	42703[3]	42704[3]	42704[3]	42707[3]	42704[3]	42703[3]
No. Merged Reflections:	9886523 (135932)[1]	4822444 (50790)[1]	2327708 (29358)[1]	2258197 (18609)[1]	1841923 (19475)[1]	7427749 (120500)[1]	10902871 (150894)[1]	3553008 (32192)[1]	2387057 (28810)[1]
	9957691 (88288)[2]	4847105 (30746)[2]	2342594 (18535)[2]	2266705 (10743)[2]	1850853 (11375)[2]	7492574 (80164)[2]	10982523 (98384)[2]	3567097 (17641)[2]	2401810 (18347)[2]
	10044009 (1397)[3]	4872185 (182)[3]	2356435 (47)[3]	2272675 (25)[3]	1857695 (18)[3]	7576103 (1587)[3]	11079889 (1945)[3]	3575821 (4)[3]	2416044 (37)[3]
Completeness (%):	100 (100)[1]	99.93 (99.42)[1]	99.86 (98.80)[1]	99.37 (94.80)[1]	99.41 (95.38)[1]	100 (100)[1]	99.99 (100)[1]	99.90 (99.07)[1]	99.75 (98.10)[1]
	99.99 (99.93)[2]	99.75 (97.92)[2]	99.33 (94.18)[2]	97.78 (81.99)[2]	97.90 (82.93)[2]	100 (100)[2]	99.99 (99.96)[2]	99.28 (93.24)[2]	99.20 (93.93)[2]
	88.38 (13.59)[3]	79.58 (2.09)[3]	75.54 (0.55)[3]	70.45 (0.29)[3]	71.09 (0.22)[3]	88.49 (14.60)[3]	89.46 (17.89)[3]	72.85 (0.05)[3]	75.53 (0.43)[3]
Signal to noise:	12.242 (4.19)[1]	8.796 (3.18)[1]	6.303 (2.59)[1]	6.574 (2.75)[1]	6.017 (3.88)[1]	10.637 (3.99)[1]	12.716 (4.26)[1]	7.900 (2.86)[1]	6.406 (2.96)[1]
	11.500 (3.37)[2]	8.295 (2.67)[2]	6.013 (2.57)[2]	6.547 (5.65)[2]	5.823 (4.10)[2]	10.016 (3.23)[2]	11.936 (3.38)[2]	7.486 (2.62)[2]	6.121 (3.11)[2]
	9.357 (4.59)[3]	7.482 (1.69)[3]	5.711 (2.14)[3]	6.453 (8.15)[3]	5.836 (0.74)[3]	8.305 (7.19)[3]	9.669 (4.27)[3]	7.648 (7.35)[3]	5.828 (2.64)[3]
Wilson b factor:	22.08[1]	22.28[1]	22.25[1]	21.80[1]	22.40[1]	22.07[1]	22.31[1]	21.95[1]	22.36[1]
	22.23[2]	22.42[2]	22.31[2]	21.93[2]	22.39[2]	22.21[2]	22.48[2]	22.08[2]	22.58[2]
	21.18[3]	21.50[3]	21.84[3]	16.17[3]	21.28[3]	21.73[3]	21.73[3]	22.54[3]	23.62[3]
R_{Split} (%):	7.45 (28.00)[1]	10.98 (39.35)[1]	15.12 (48.15)[1]	14.57 (50.17)[1]	16.92 (52.66)[1]	8.81 (28.97)[1]	7.09 (27.15)[1]	10.91 (43.80)[1]	13.78 (50.14)[1]
	7.61 (36.70)[2]	11.16 (51.44)[2]	15.26 (49.46)[2]	14.63 (49.95)[2]	16.98 (53.67)[2]	8.97 (37.75)[2]	7.25 (35.41)[2]	11.08 (56.75)[2]	13.95 (59.70)[2]
	8.06 (126.90)[3]	11.35 (N/A)[3]	15.32 (N/A)[3]	14.65 (N/A)[3]	16.99 (N/A)[3]	9.40 (90.12)[3]	7.71 (62.87)[3]	11.12 (N/A)[3]	14.07 (N/A)[3]
CC*:	1.00 (0.96)[1]	0.99 (0.92)[1]	0.98 (0.90)[1]	0.99 (0.89)[1]	0.98 (0.85)[1]	1.00 (0.96)[1]	1.00 (0.97)[1]	0.99 (0.90)[1]	0.99 (0.86)[1]
	1.00 (0.94)[2]	0.99 (0.88)[2]	0.98 (0.89)[2]	0.99 (0.88)[2]	0.98 (0.88)[2]	1.00 (0.93)[2]	1.00 (0.95)[2]	0.99 (0.84)[2]	0.99 (0.83)[2]
	1.00 (N/A)[3]	0.99 (N/A)[3]	0.98 (N/A)[3]	0.99 (N/A)[3]	0.98 (N/A)[3]	1.00 (0.79)[3]	1.00 (0.92)[3]	0.99 (N/A)[3]	0.99 (N/A)[3]
$CC_{1/2}$:	0.99 (0.87)[1]	0.97 (0.74)[1]	0.94 (0.68)[1]	0.95 (0.65)[1]	0.92 (0.56)[1]	0.98 (0.86)[1]	0.99 (0.88)[1]	0.98 (0.68)[1]	0.96 (0.59)[1]
	0.99 (0.80)[2]	0.97 (0.64)[2]	0.94 (0.67)[2]	0.95 (0.63)[2]	0.92 (0.63)[2]	0.98 (0.76)[2]	0.99 (0.81)[2]	0.98 (0.55)[2]	0.96 (0.52)[2]
	0.99 (0.05)[3]	0.97 (N/A)[3]	0.94 (N/A)[3]	0.95 (N/A)[3]	0.92 (N/A)[3]	0.98 (0.45)[3]	0.99 (0.74)[3]	0.98 (N/A)[3]	0.96 (N/A)[3]

*Pump-dump delay: 500 fs †Pump-dump delay: 2ps [1,2,3] Represent merged statistics for distinct resolution shells N/A is used for when statistical measures for the inner shell where unphysical.

5.4. PAL XFEL 2020-2nd-NCI-007

Figure 1 shows data from experiments collected at PAL XFEL 2020-2nd-NCI-007. This was a control experiment, collecting TR-SFX 100 µs pump-probe delay to confirm that photoisomerization was complete by this time. The FSC is shown in Supplementary Fig. 43. Q-weight difference maps of 100 µs and negative laser on data in Supplementary Fig. 44 clearly shows the emergence of cis photoproduct after 100 µs. Occupancy refinement for the 100 µs and negative data sets are shown in Supplementary Fig. 45 & Supplementary Fig. 46 respectively. Crystallographic statistics are shown in Supplementary Table. 6.

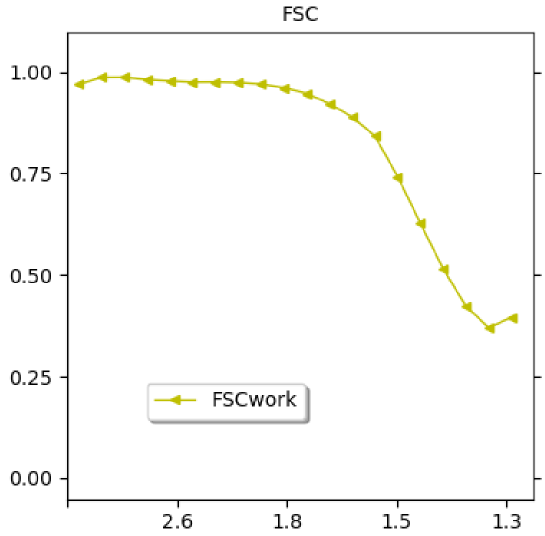

Supplementary Fig. 43 | PAL-XFEL 2020-2nd-NCI-007 The Fourier shell coefficient (FSC) and all merged laser off, dark data. An FSC of 0.5 is chosen for a limiting 1.42 Å.

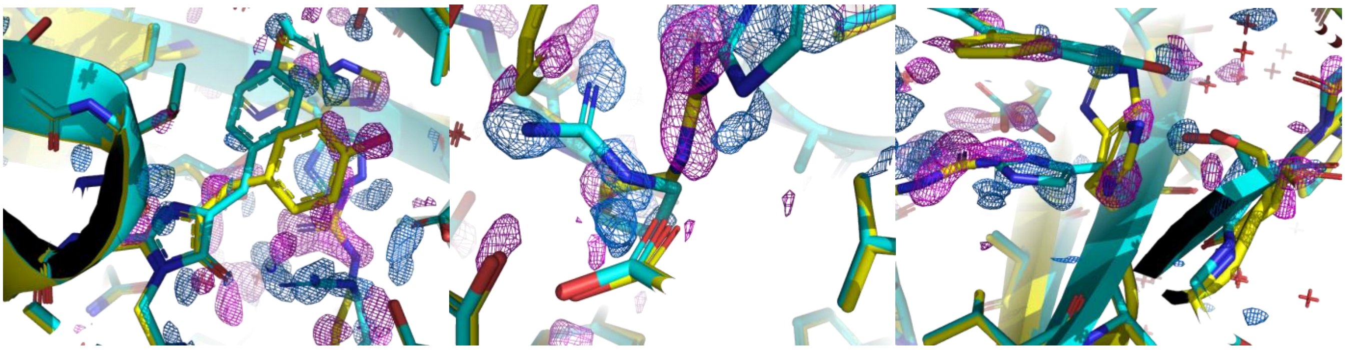

Supplementary Fig. 44 | PAL-XFEL TR-SFX PP 100 µs difference maps. Q-weighted difference electron density between the 400 nm 100 µs and negative 1 ps laser-on, dark data. The ground state coordinates are shown in yellow and cis, photproduct coordinates are shown in blue. Electron density contoured at +2 rms (blue) and -2 rms (magenta). Signals clearly show emergence of the photoproduct after 100 µs.

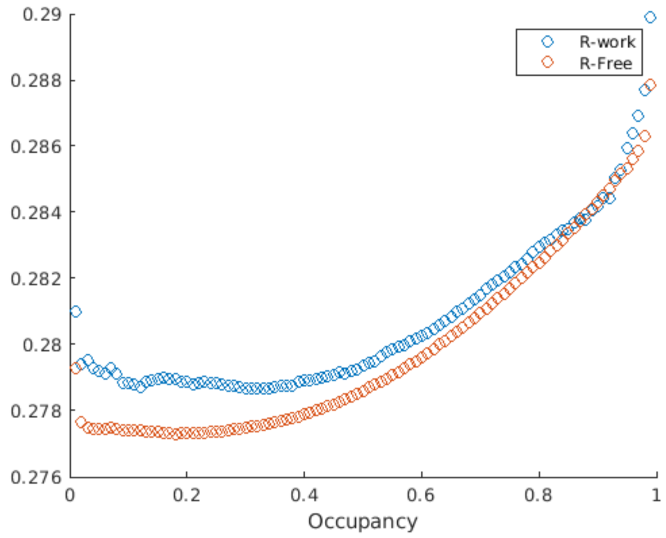

Supplementary Fig. 45 | PAL-XFEL TR-SFX PP 100 µs occupancy refinement. 100 µs-laser-on: R_{work}/R_{free} calculation for varied occupancy of refined coordinates. Occupancy refers to the proportion of ground state (trans) coordinates (dark) compared to the photoproduct (cis) coordinates. R_{fac} (and R_{free}) minimize at photoproduct = 0.18 (0.21) and dark = 0.82 (0.79).

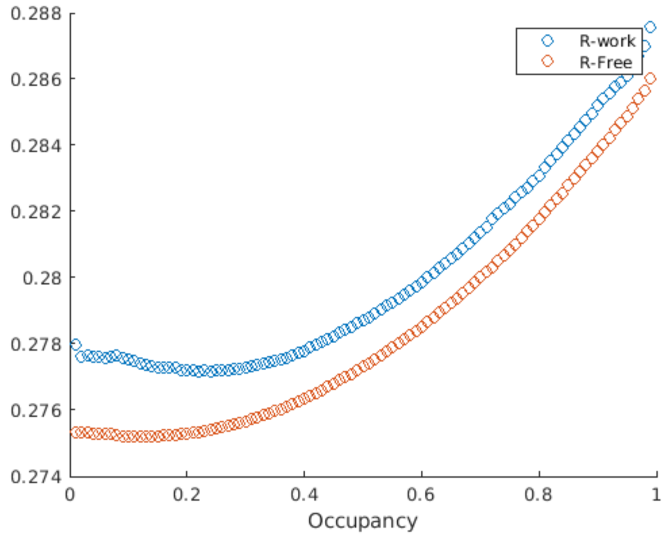

Supplementary Fig. 46 | PAL-XFEL TR-SFX PP negative delay occupancy refinement. Neg-1ps-laser-on: R_{work}/R_{free} calculation for varied occupancy of refined coordinates. Occupancy refers to the proportion of ground state (trans) coordinates (dark) compared to the photoproduct (cis) coordinates. R_{fac} (and R_{free}) minimize at photoproduct = 0.12 (0.16) and dark = 0.88 (0.84).

Supplementary Table. 6 | PAL-XFEL TR-SFX PP crystallographic statistics

	neg-1ps-laser-on	100us-laser-on
Indexed Patterns:	49465	60993
Resolution Limits (Å):	78.69-1.55 (1.605-1.550)[1]	78.69-1.55 (1.605-1.550)[1]
	78.69-1.45 (1.502-1.450)[2]	78.69-1.45 (1.502-1.450)[2]
	78.69-1.35 (1.398-1.350)[3]	78.69-1.35 (1.398-1.350)[3]
Number of Unique reflection indices:	34233[1]	34233[1]
	41638[2]	41638[2]
	51398[3]	51398[3]
No. Merged Reflections:	11752976 (247091)[1]	14106334 (252617)[1]
	12005396 (92501)[2]	14357015 (91162)[2]
	12060432 (14662)[3]	14403050 (10532)[3]
Completeness (%):	100.00 (100.00)[1]	100.00 (100.00)[1]
	99.96 (99.59)[2]	99.98 (99.78)[2]
	95.90 (64.79)[3]	95.02 (56.33)[3]
Signal to noise:	6.648 (1.95)[1]	7.601 (1.81)[1]
	5.685 (0.97)[2]	6.434 (0.76)[2]
	4.905 (0.56)[3]	5.537 (0.18)[3]
Wilson b factor:	23.39[1]	25.62[1]
	23.52[2]	26.28[2]
	22.87[3]	26.54[3]
R_{Split} (%):	10.18 (53.79)[1]	8.64 (56.11)[1]
	10.58 (114.57)[2]	8.96 (146.34)[2]
	10.85 (236.85)[3]	9.16 (419.81)[3]
CC*:	1.00 (0.90)[1]	1.00 (0.91)[1]
	1.00 (0.69)[2]	1.00 (0.63)[2]
	1.00 (0.55)[3]	1.00 (0.06)[3]
$CC_{1/2}$:	0.99 (0.68)[1]	0.99 (0.69)[1]
	0.99 (0.31)[2]	0.99 (0.25)[2]
	0.99 (0.18)[3]	0.99 (0.00)[3]

[1,2,3] Represent merged statistics for distinct resolution shells

6. Photo conversion quantum yield measurements

The photo conversion (photo-product) yields of both the off→on and on→off reaction were measured in both solution and in crystalline samples of rsKiiro. Solution sample was prepared in a Harrick cell with 25.6 µm spacer producing an OD of ~0.2 @ 488 nm, crystal "pancakes" were prepared in the method described[12] with final OD of ~0.3 @ 488 nm.

off → on: a 488 nm CW laser was tightly focused (41x47 µm FWHM) and directed onto an amplified photodiode (Thorlabs PDA36A2) and attenuated to be weakly actinic, (t_{conv} > 20 s). The sample was fully converted to the off state and then illuminated with a loosely focused 405 nm CW laser (177x178 µm FWHM) at a range of powers (8-22 µW) and the kinetic rate measured using a digital oscilloscope (Picoscope). The total switch rate from off→on:

$$off \xrightarrow{k_{FT},-k_{BT},k_{TH}} on$$

Where k_{FT}, k_{BT} & k_{Th} are the total forward and backwards rates and the thermal recovery rate. As the values of k_{BT} (>0.05 s^{-1}) & k_{Th} (4x10^{-4} s^{-1}at 293 K (Extended Data Fig. 1c) are significantly smaller than switching rates observed at the power densities used (1-5 s^{-1}) both can be disregarded and a single rate considered, therefore

$$k_{FT} = \phi_{off\rightarrow on} P_d \sigma_{405}$$

Where $\phi_{Off\rightarrow On}$, P_d and σ_{405} is the total off → on photoconversion quantum yield, photon density per second and single molecule cross section at 405 nm (1.38x10^{-16} cm^2) respectively. $\phi_{Off\rightarrow On}$ was found to be 15.9±1.2% in solution and 18.3±2.2% in the crystalline sample.

on → off: The measurement was repeated for other reaction, in this case the roles 488 nm and 405 nm. CW lasers were reversed and the focus sizes adjusted accordingly. $\phi_{On\rightarrow Off}$ was determined to be 0.63±0.03% in solution and 0.12±0.01% in the crystalline sample.

Interestingly this shows that the quantum yield in the on→off reaction is 5 times smaller in a crystalline sample, while the off→on is 1.15 times larger.

7. Time-correlated single photon counting (TCSPC)

Determination of the excited state barrier height using temperature-dependent fluorescence lifetime measurements was described previously in depth [13]. Briefly, fluorescence lifetime decays were measured on a Horiba Fluorolog-3 fluorescence spectrometer using time-correlated single photon counting (TCSPC). Sample temperature was controlled with a water bath (NESLAB RTE-7 circulating bath, Thermo Fisher Scientific) and measured with a thermocouple (870 Digital Thermometer, Keithley). Samples were diluted to 1.5 µM (A_{487} ~0.1) with buffer containing 10 mM Tris HCl (Fisher, CAS 1185-53-1) at pH 8.0, 50 mM NaCl (Fisher, CAS 7647-14-5), and 30% glycerol (Fisher, CAS 56-81-5) (v/v) in a 1.5 mL quartz cuvette (Hellma Analytics). The cuvette was equilibrated at the desired temperature for 5 minutes before data acquisition. The sample was excited with 487 nm light polarized at 0 degrees from a Fluorolog EXTREME supercontinuum laser (pulse width ~20-40 ps), and fluorescence emission polarized at the magic angle (55°) was detected at 504 nm. Three separate measurements were obtained at each temperature. The excitation and emission slit widths were set to 5 nm to keep the detection rate

below 2%. The instrument response function was measured at 487 nm by scattered light from a solution of LUDOX® HS-40 colloidal silica (Sigma, CAS 7631-86-9).

Representative fluorescence decays at each temperature from 278K to 298K are shown in Extended Data Fig. 1i. The decay data was analyzed using the DecayFit software in MATLAB[14]. In brief, the program performs an iterative reconvolution of the instrument response function (IRF) with a simulated decay model using a least squares analysis, allowing for a channel shift between the IRF and each decay. By analyzing the chi-squared values and the residuals plot, we determined that a triple exponential model best represents the data. Supplementary Table. 7 summarizes the average fluorescence lifetimes, relative populations, and standard deviations from the triplicate measurements at each temperature.

The excited state energy barrier was determined by fitting the temperature-dependent fluorescence lifetimes to Equation (S1), assuming an Arrhenius behaviour of barrier crossing:

$$\frac{1}{\tau} = \mathcal{A}_1 + \mathcal{A}_2 e^{-\frac{E_\alpha}{RT}} \qquad \text{(S1)}$$

Where $\mathcal{A}_1$ and $\mathcal{A}_2$ are temperature independent constants and E_α is the excited state energy barrier height. See Supplementary Text S6 in Romei et al.[13] for a derivation of this Arrhenius equation and validation of the Arrhenius behaviour of barrier crossing. As shown in Supplementary Table. 7, the population of the slow lifetime component is less than 3%. Lifetime components with minimal populations are broad and carry large uncertainties (see Supplementary Texts S6 and S7 in Romei et al.[13]), which prevents accurate extraction of any temperature dependence. The fast and middle lifetime components from the triple exponential decay, which comprise >97% of the population, were each fit to the Arrhenius equation. To accurately calculate the energy barrier and its corresponding confidence interval, a parametric bootstrap analysis was performed (see Materials and Methods in Romei et al.[13] for MATLAB code and further details). The fitting parameters from the bootstrap analysis are listed in along with confidence intervals that report one standard deviation from the median, and the fit from the bootstrap are shown in Supplementary Table. 8 and plotted in Extended Data Fig. 1j with the temperature-dependent fluorescence lifetimes for the fast and middle lifetime components.

	1st comp.			2nd comp.			3rd comp.		
Temp (K)	Avg. Pop	k*	SD*	Avg. Pop	k*	SD	Avg. Pop	k*	SD*
278.45	0.03	0.54	0.03	0.35	1.4	0.07	0.61	4.14	0.2
280.15	0.027	0.54	0.02	0.34	1.42	0.07	0.64	4.15	0.3
282.15	0.026	0.55	0.01	0.35	1.5	0.01	0.63	4.57	0.02
284.15	0.023	0.54	0.03	0.33	1.53	0.08	0.64	4.58	0.3
286.05	0.02	0.53	0.005	0.34	1.57	0.01	0.64	4.7	0.06
287.95	0.021	0.54	0.007	0.34	1.67	0.007	0.64	5.14	0.05
289.85	0.019	0.54	0.01	0.33	1.71	0.06	0.65	5.3	0.3
291.75	0.017	0.52	0.02	0.3	1.68	0.08	0.69	5.11	0.3
293.75	0.017	0.53	0.01	0.33	1.84	0.04	0.65	5.87	0.2
295.75	0.017	0.54	0.008	0.3	1.84	0.01	0.68	5.7	0.1
297.55	0.015	0.53	0.002	0.31	1.93	0.02	0.68	6.08	0.2

*Rate components k, and standard deviation SD, all in units of (ns^{-1})

Supplementary Table. 7 TCSPC rates. Temperatures, average populations, lifetime rates (k), and standard deviations (SD) are shown for the three components fitted to the traces in Extended Data Fig. 1i

The fast and middle fluorescence lifetimes as a function of temperature yielded excited state energy barriers of 3.6 and 4.0 kcal mol^{-1}(or 15.0 and 16.73 kJ mol^{-1}), respectively, which are similar within error. The observation of multiple lifetimes in fluorescent proteins is not unusual[15–17] may suggest the

presence of multiple ground state conformations or increased flexibility within the chromophore pocket[18,19](Supplementary Fig. 48 - Supplementary Fig. 50). Interestingly, regardless of the origin of this heterogeneity, the excited state energy barrier height is largely conserved.

Component	$\mathcal{A}_1$ (ns^{-1})	$\mathcal{A}_1$ CI* (1σ, ns^{-1})		$\mathcal{A}_2$ (ns^{-1})	$\mathcal{A}_2$ CI* (1σ, ns^{-1})		ESB† (kcal/mol)	ESB† CI* (1σ, kcal/mol)	
Fast	0.241	3E-14	1.417	2.40E+03	1.84E+03	8.83E+03	3.576	3.392	4.439
Middle	0.513	0.312	0.684	1.24E+03	8.79E+02	1.70E+03	4.008	3.749	4.252
Slow	N/A								

*CI: confidence interval upper and lower bounds, † ESB: Excited state barrier (kcal/mol)

Supplementary Table. 8 Fitting parameter from bootstrap Arrhenius plots of the TCSPC recovered rates.

This data further supports the efficient photoswitching behaviour of rsKiiro. Since fluorescence and energy barrier crossing are competing processes in the excited state, the observed low excited state energy barrier height explains the low fluorescence quantum yield compared to other fluorescent proteins. Instead of emitting a photon through fluorescence, the chromophore crosses the energy barrier along the bond rotation reaction coordinate. The magnitudes of the fluorescence lifetimes also support this conclusion. Typical fluorescence lifetimes in fluorescent proteins are on the order of ns and show strong positive correlations with fluorescence quantum yield[20]. The fast and middle components of rsKiiro are <300 ps and <750 ps, respectively, meaning that most of the chromophore population crosses over the excited state en

8. Fluorescence spectra and fluorescence quantum yield

The absolute fluorescence quantum yield (FQY) for the cis on state was determined as previously described [13,21]. Briefly, fluorescence spectra were measured on a PerkinElmer LS 55 fluorescence spectrometer. Excitation and emission slit widths were set to 1.0 nm. The sample was excited at 487 nm, and the emission spectrum was obtained every 0.5 nm at 120 nm/min. Sample absorption was determined using a PerkinElmer Lambda 365 UV-vis spectrophotometer and then converted into absorption factor according to the following equation: $f = 1 - 10^{-A_{488}}$, where is the absorption factor and is the absorbance at 488 nm. The absorption factor was plotted against the integrated emission spectrum for different protein concentrations, and the slope of the linear fit was compared against that of fluorescein (Aldrich, CAS 2321-07-5) in 0.1 M NaOH, a known standard with a reported absolute FQY of 0.90 at 488 nm [22]. The equation used to calculate FQY is as follows:

$$FQY_X = FQY_{ST}\left(\frac{Slope_X}{Slope_{ST}}\right)\left(\frac{\eta_X^2}{\eta_{ST}^2}\right) \qquad \text{(S2)}$$

where X and ST reference the sample of interest and the fluorescein standard, respectively, slope refers to the slope of the linear fit mentioned above, and η is the refractive index of the solution.

To measure the FQY of the trans off state, a similar protocol as described above was used, although samples were illuminated for one minute with a 30 mW 488 nm diode laser (85-BCD-030-115, Melles Griot) immediately prior to all absorption and fluorescence measurements. Emission spectra were measured by exciting at 387 nm. Coumarin 153 (Sigma, CAS 53518-18-6), which has a reported absolute FQY of 0.53 in ethanol [21], was used as the FQY standard to properly match the excitation wavelength with the protonated trans rsKiiro chromophore.

The fluorescence quantum yield (FQY) of rsKiiro in the native cis on state is 6.8 ± 0.1%. FQYs of traditional GFPs are an order of magnitude greater, roughly spanning a range between 50 and 90%[20].

Such a low FQY of rsKiiro suggests that nonradiative decay processes such as chromophore photoisomerization mostly outcompete fluorescence in the excited state, and the determination of fast fluorescence lifetime decays and a low excited state energy barrier height further supports this conclusion. The FQY of the trans off state is 0.49 ± 0.03%, an order of magnitude lower than that of the cis on state. The decreased FQY for the trans state could be attributed to increased flexibility of the protein and the chromophore and indicates that excited state proton transfer does not likely occur.

8.1. Fluorescence lifetime estimate from Strickler Berg relation

In order to evaluate the rate corresponding to fluorescence decay we use the Strickler-Berg relation[23]

$$Fk_{fl} = 3 \times 10^{-9} n^2 \langle \bar{\nu}^{-3} \rangle_{em}^{-1} \int \frac{\varepsilon(\bar{\nu})}{\bar{\nu}} d\bar{\nu} \, (s^{-1}) \quad \text{(S3)}$$

Using an extinction coefficient for the neutral trans chromophore ε = 32,909 $M^{-1}cm^{-1}$. Calculation using the absorption (390 nm maximum) and fluorescence (490 nm maximum, Extended Data Fig. 1b) spectra and approximately 60 nm FWHM spectral parameters, the Strickler-Berg relationship calculates a pure radiative lifetime of 5.34 ns when using a value of n=1.33 for water. Taking the experimental value for the fluorescence quantum yield of the off state φ(off) = 0.49 ± 0.03%, the resulting fluorescence lifetime is τ_{fl} = 30 ps. This value is very close to the experimental value of excited state decay measured by TA spectroscopy to be 50 ps (see above and Extended Data Fig. 1d). The rate k_{fl} = $2x10^{10}$ s^{-1} for the off state. Similarly, for the on-state cis anioic chromophore using a value of 98,727 $M^{-1}cm^{-1}$ and values for the absorption and emission of 485 nm and 515 nm, the pure radiative lifetime is calculated as 2.28 ns. Using the experimentally determined fluorescence quantum yield of φ(off) = 6.8 ± 0.1%, the calculated fluorescence lifetime is τ_{fl}=150 ps.

9. Cyro-trapping of rsKiiro intermediates

To gain insight into the reaction pathways of rsKiiro and attempt to isolate a reaction intermediate, Arrhenius measurements of both the on→off and off→on reactions were performed over a range of cryogenic temperatures (190-293 K). A small volume of rsKiiro solution was mixed with glycerol (to avoid icing, 1:4 ratio) and loaded onto a CryoLoop (Hampton Research) suspended inside an inverted microscope to monitor change in absorption. The sample was cooled using a cryo stream (Oxford Instruments). The on→off and off→on were driven using a 480 nm and 400 nm LED's respectively, focused onto the loop using f=100 mm lenses. The off→on reaction was performed both when the pre-conversion was performed at the same cryo temperature and at room temperature. The results are shown in Fig 2 (right). A full description of the methodology and fitting is described in the supplementary discussion.

9.1. Steady state MX Cryo-structures

Following the observation of Arrhenius break in rsKiiro photo-switching reaction in both the forward and reverse reaction (Extended Data Fig. 1e & k), crystals were prepared in attempt to cryo-trap structural intermediates at cool temperatures. Supplementary Fig. 47 shows the preparation of each state.

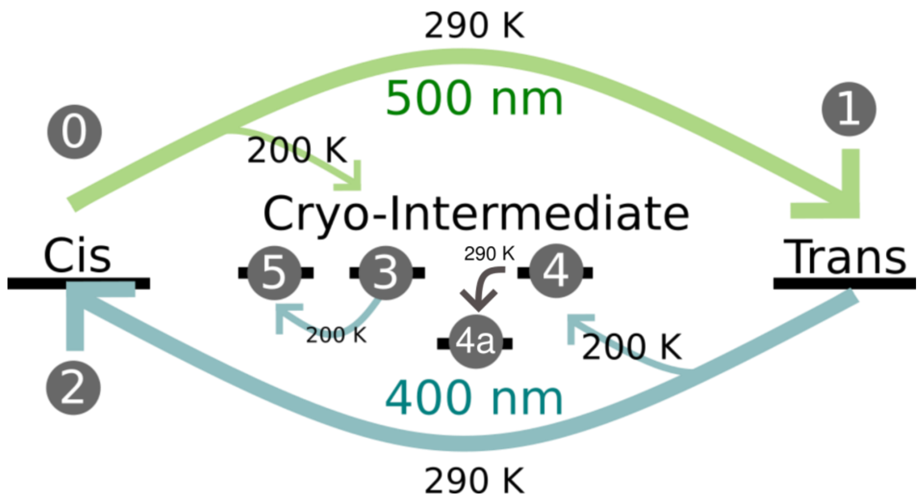

Supplementary Fig. 47 | Diagram showing the preparation of different states from illumination schemes at temperatures 290 K and 200 K using 500 nm and 400 nm light and existence of possible cryo-trapped-intermediate structures as indicated by the Arrhenius behaviour.

The intermediate structures 3 and 4 should be thermally unstable at room temperature. This means annealing a crystal should relax the structure from the intermediate to a normal cis or trans state. If the crystal is oriented correctly (orthogonal to the X-ray beam) then multiple data collections from the same crystal can be performed and the crystal annealed in between. Comparison of the annealed and un-annealed structures should then provide strong evidence for the presence of any cryo-trapped intermediates.

Batch grown crystals around 10x10x100 µm in size were grown in conditions of 25% Poly-ethylene glycol 3350 in 0.2 M lithium sulphate, 0.1 M Tris-HCl pH 8.5 with a final protein concentrating of 10 mg/ml (similar to Hutchison et al.[24]). A single crystal was fished onto an appropriately sized loop and illuminated. To ensure that the heating effects of illumination were accounted for the same power densities used in the spectroscopy were used in the crystal preparation. A crystal was assumed to have an optical density of 1. Using the measured rate constants at 200 K it was calculated 95% conversion would occur after 30 minutes of 400 nm and 69 minutes of 500 nm illumination respectively. As soon as illumination time was complete the crystals were flash frozen into liquid nitrogen. Data collection occurred at Diamond Light Source, Oxfordshire during beamtimes MX22819-1 and MX17221-42 at beamline i03 using an exposure time of 10 ms for 3600 images at 100% transmission for wavelengths 0.97 Å or 0.73 Å. Structure 3 and 4 were annealed by blocking the 100 K cryo-stream for a time of 1 s. After measurement of the cryo trapped structure, the crystal was annealed and another structure collected at a shifted point on the crystal. X-ray diffraction data was merged using the STARANISO[10] package.

Difference map were then calculated as described above. Q-weighted difference maps of 0 – 1 and 0 – 4 and 0 – 4a are shown in Supplementary Fig. 48, Supplementary Fig. 49 and Supplementary Fig. 50 respectively. An intermediate state was found in structure 4 (Supplementary Fig. 50). Crystallographic data and refinement statistics are shown in Supplementary Table. 9.

Supplementary Table. 9 | MX-Crystallography statistics for cryo-trapping processed with STARANISO[10].

**Condition**	**0**<br>**On state:**<br>**Cis Structure**<br>**(unilluminated)**			**1**<br>Off state<br>**Trans Structure**<br>**(illuminated at 290 K)**			**4**<br>**Cis structure intermediate**<br>**(illuminated at 290 K then 200 K)**			**4a**<br>**Thermal annealing at 290 K of the cis intermediate structure**		
PDB ID	7QLI			7QLJ			7QLK			7QLL		
Spacegroup name	$P2_12_12_1$			$P2_12_12_1$			$P2_12_12_1$			$P2_12_12_1$		
Unit cell parameters	38.560 73.467 78.038 90.0 90.0 90.0			38.7470 73.5243 78.1062 90.0 90.0 90.0			38.648 73.746 78.142 90.0 90.0 90.0			38.671 73.803 78.111 90.0 90.0 90.0		
Wavelength (Å)	0.72932			0.91587			0.72931			0.72932		
	Overall	Inner Shell	Outer Shell	Overall	Inner Shell	Outer Shell	Overall	Inner Shell	Outer Shell	Overall	Inner Shell	Outer Shell
Low resolution limit	53.549	53.549	1.261	39.053	39.053	1.039	53.633	53.633	1.546	53.645	53.645	1.4
High resolution limit	1.155	3.526	1.159	1.02	2.838	1.02	1.459	4.277	1.459	1.336	3.917	1.336
Rmerge (all I+ & I-)	0.122	0.044	2.088	0.063	0.035	0.777	0.135	0.053	1.914	0.087	0.062	1.404
Rmerge (within I+/I-)	0.123	0.044	2.028	0.061	0.034	0.705	0.136	0.052	1.819	0.086	0.061	1.287
Rmeas (all I+ & I-)	0.127	0.046	2.171	0.069	0.038	0.873	0.14	0.056	1.988	0.091	0.064	1.534
Rmeas (within I+/I-)	0.133	0.047	2.194	0.073	0.04	0.889	0.147	0.056	1.964	0.094	0.066	1.53
Rpim (all I+ & I-)	0.035	0.013	0.593	0.027	0.015	0.392	0.039	0.016	0.534	0.026	0.018	0.597
Rpim (within I+/I-)	0.05	0.018	0.836	0.039	0.021	0.533	0.055	0.021	0.739	0.036	0.025	0.808
Total number of observations	791089	37596	39592	688584	35224	27383	452492	20581	23068	496420	27160	13721
Total number unique	59836	2990	2992	112855	5642	5645	34513	1725	1725	44451	2222	2224
Mean(I)/sd(I)	12.1	46.4	1.4	12.8	38.1	2.2	10.7	32.1	1.4	15.7	38.7	1.2
Completeness (spherical)	77	100	17.5	98.9	100	94.3	87	99.9	27.6	86.3	100	33.5
Completeness (ellipsoidal)	94.8	100	63.7	98.9	100	94.3	94.1	99.9	49.6	90.6	100	47.1
Multiplicity	13.2	12.6	13.2	6.1	6.2	4.9	13.1	11.9	13.4	11.2	12.2	6.2
CC(1/2)	0.999	0.999	0.5	0.999	0.999	0.593	0.999	0.999	0.643	0.999	0.999	0.455
**Refinement:**												
Resolution	53.55 - 1.16			39.05 - 1.02			53.69 - 1.46			53.70-1.32		
No. reflections all/free	59836 / 2888			112852 / 5501			34516 / 1672			44562 / 2261		
R-factor/R-free	0.161 / 0.179			0.122 / 0.140			0.172 / 0.197			0.169 / 0.198		
RMS Deviations:												
Bonds	0.0134			0.0226			0.0126			0.0133		
Angles	1.811			1.542			1.834			1.844		

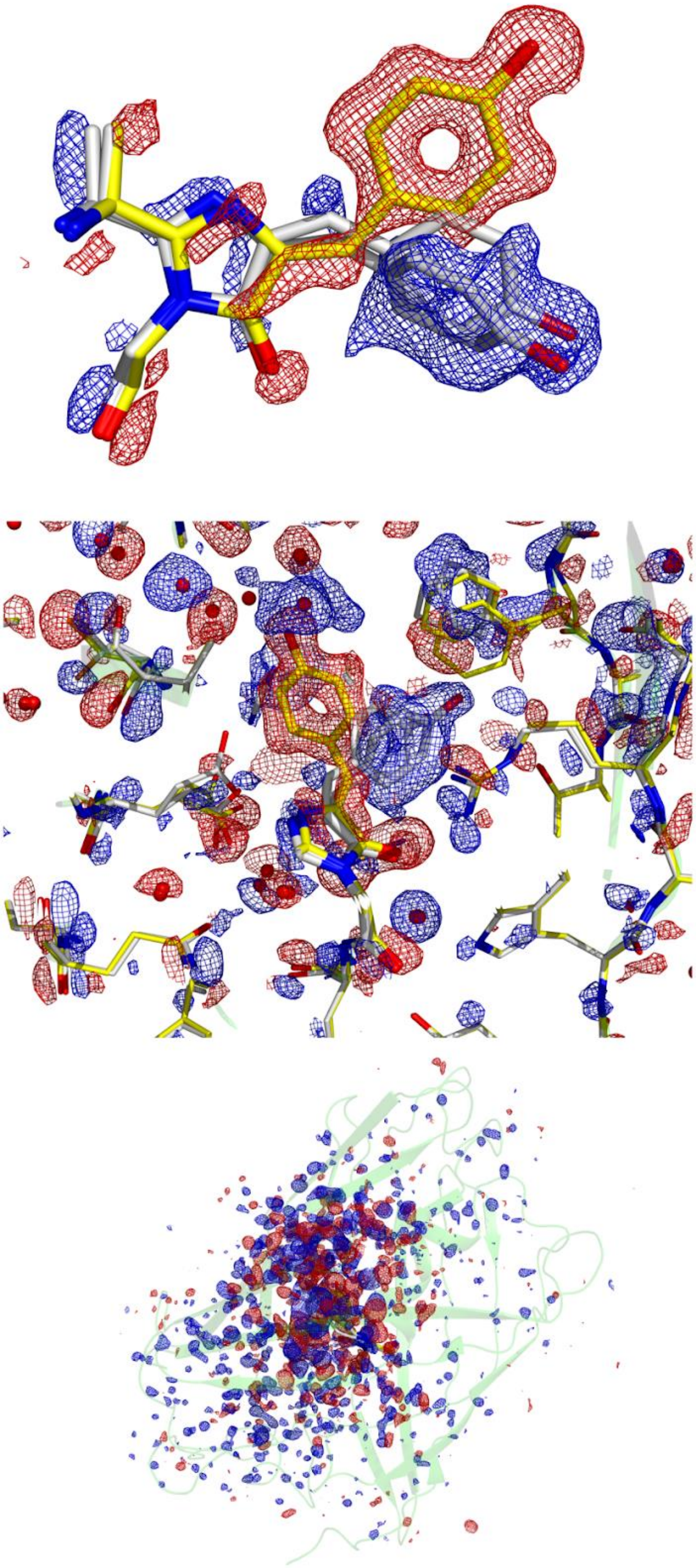

Supplementary Fig. 48 | MX Crystallography trans-cis photoisomerization. Q-weighted difference electron density of cryo-MX structures **0** (on state) (PDB ID 7QLI) and **1** (off state by 500nm illumination at 290K) (PDB ID 7QLJ) (Supplementary Fig. 47). The secondary structure (green) of the cis-ground state coordinates (yellow) and trans-state model (grey) are shown with electron density contoured at +3 rms (blue) and -3 rms (red). Note the additional chromophore conformation in the trans state with partial occupancy that is not observed at room temperature SFX data.

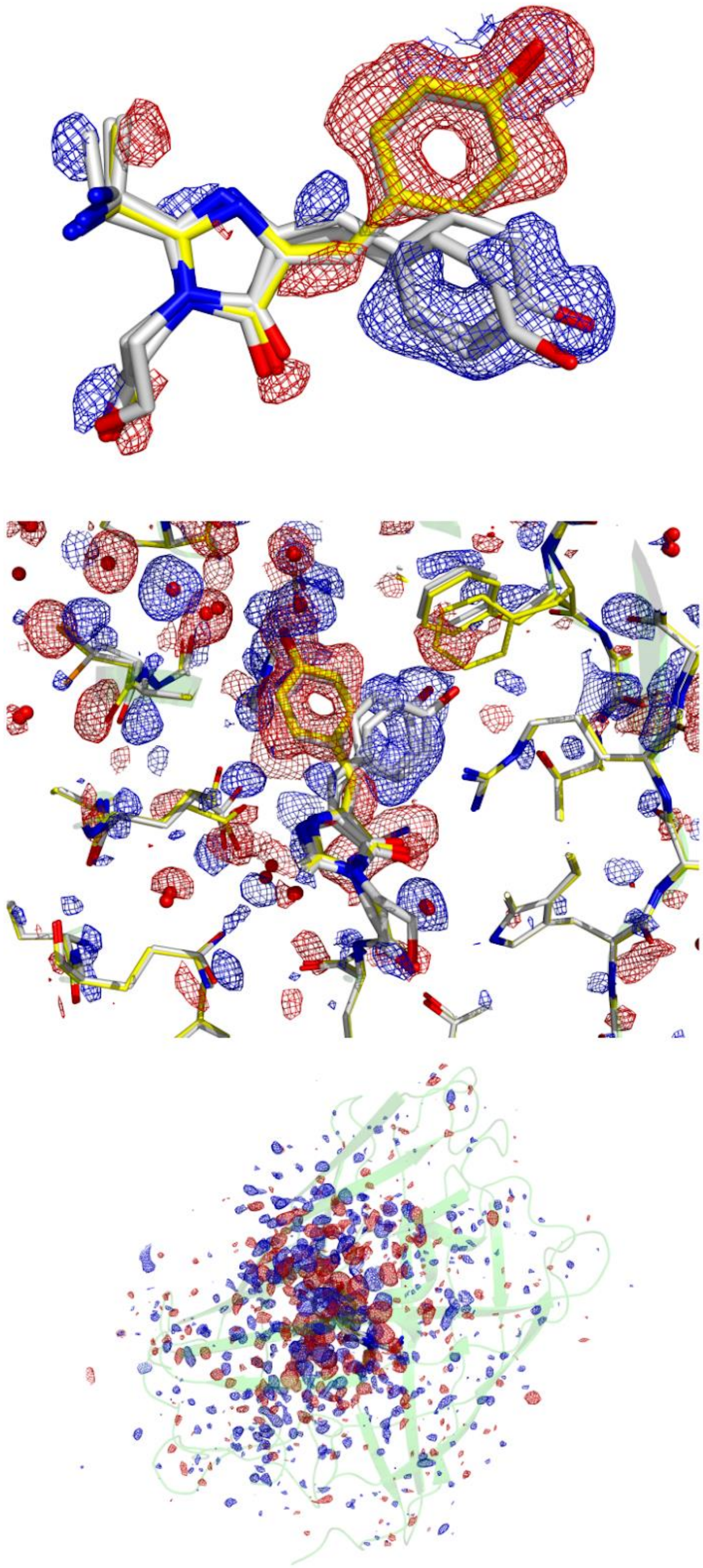

Supplementary Fig. 49 | MX Crystallography 200 K photoisomerization. Q-weighted difference electron density of cryo-MX structures **0** (on state) (PDB ID 7QLI) and **4** (off state by 500nm illumination at 200K) (PDB ID 7QLK). Showing that cis-trans photoisomerization proceeds at 200 K. The cis ground state (yellow carbon chromophore structure) was illuminated with 500 nm light at 200 K temperature. The photoproduct shows the same two trans chromophore structures (grey carbon chromophore structures), and associated amino acid changes as seen at 293 K temperature (Supplementary Fig. 48). Electron density contoured at +3 rms (blue) and -3 rms (red).

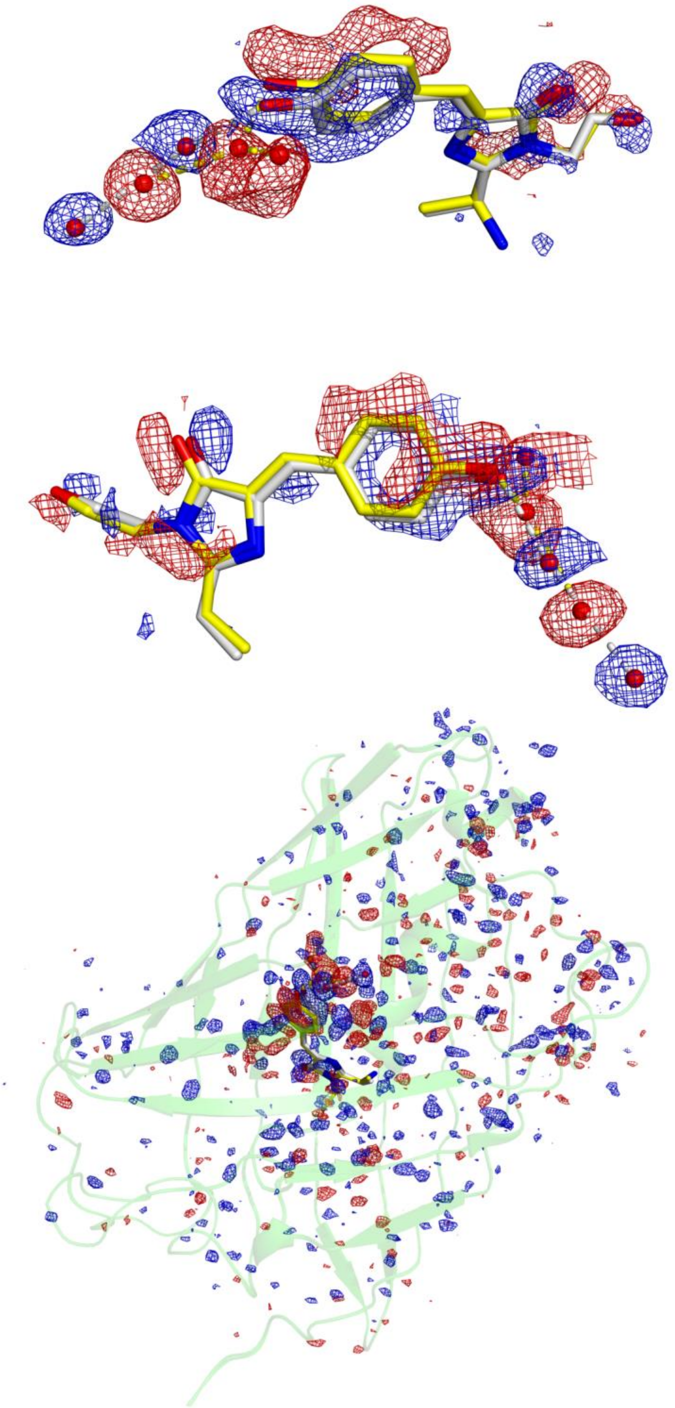

Supplementary Fig. 50 | MX Crystallography Thermal annealing of cryo-trapped intermediate. Thermal annealing of a photoproduct in the trans-cis direction: Q-weighted difference electron density of cryo-MX structures between **4** and the annealed crystal (**4a**). A trans structure was accumulated at 293 K to full occupancy. The relaxed trans structure was cooled to 200 K and subsequently illuminated with 400 nm light. The resulting photoproduct (Structure '**4**' in Supplementary Fig. 47) had fully occupied cis- chromophore conformation (yellow carbon coordinates).The photoproduct was heated to 293 K for 1 second then cryo-cooled for MX data collection (Structure '**4a**') (grey carbon coordinates). Isomorphous difference maps are shown (red, -3 rms; blue, 3 rms).

Supplementary Discussion

10. rsKiiro development

The target sample for these studies is a photoswitching fluorescent protein named "rsKiiro". Part of the EosFP family of fluorescent proteins it is a mutant of "Skylan-NS"[25]. It was the culmination of >80 iterations selected for 3 factors: increased photoproduct quantum yield, improved diffraction quality and resolution following photoconversion and expression yield. Potential mutants were developed using site-directed mutagenesis of high performing photoswitching fluorescent proteins reported in the literature which, in addition to Skylan-NS, included TGP[26], rsTagRFP[27] and rsEGFP2[28]. Mutations targeted key side chains in and around the chromophore region that have been reported to influence photo-switching behaviour in similar systems. rsKiiro is a L62A / M159T mutant of Skylan-NS, A rsFP originally derived from a species of coral known as *Lobophyllia Hemprichii*[25]. Structurally, rsKiiro is similar to Green Fluorescent Protein (GFP) with an 11-stranded beta-barrel surrounding an alpha helix. The chromophore of rsKiiro is formed from the Ala-Tyr-Gly tripeptide at residue numbers 62-64 in the alpha helix. During folding an autocatalytic condensation reaction produces the 4-(p-hydroxybenzylidene)-5-imidazolinone (p-HBI) chromophore. The optimized sequence included the 'fast-switching' M159T mutation that also yields similar phototransformation improvements also seen in the Dronpa FP[29]. The mutation retained the excellent crystallographic statistics, resolution and quality after photoconversion as also seen in the 'Skylan-NS' crystals[24].

```
CLUSTAL O(1.2.4) multiple sequence alignment


EosFP           MSAIKPDMKINLRMEGNVNGHHFVIDGDGTGKPFEGKQSMDLEVKEGGPLPFAFDILTTA     60
rsKiiro         MSAIKPDMKIKLRMEGNVNGHHFVIDGDGTGKPFEGKQSMDLEVKEGGPLPFAFDILTTA     60
skylan-NS       MSAIKPDMKIKLRMEGNVNGHHFVIDGDGTGKPFEGKQSMDLEVKEGGPLPFAFDILTTA     60
                **********:*************************************************

EosFP           FHYGNRVFAEYPDHIQDYFKQSFPKGYSWERSLTFEDGGICIARNDITMEGDTFYNKVRF     120
rsKiiro         FAYGNRVFAKYPDNIQDYFKQSFPKGYSWERSLTFEDGGICNARNDITMEGDTFYNKVRF     120
skylan-NS       FLYGNRVFAKYPDNIQDYFKQSFPKGYSWERSLTFEDGGICNARNDITMEGDTFYNKVRF     120
                * *******:***:*************************** ******************

EosFP           HGVNFPANGPVMQKKTLKWEPSTEKMYVRDGVLTGDITMALLLEGNAHYRCDFRTTYKAK     180
rsKiiro         YGTNFPANGPVMQKKTLKWEPSTEKMYVRDGVLTGDVETALLLEGNAHYRCDFRTTYKAK     180
skylan-NS       YGTNFPANGPVMQKKTLKWEPSTEKMYVRDGVLTGDVEMALLLEGNAHYRCDFRTTYKAK     180
                :*.*********************************:  *********************

EosFP           EKGVKLPGYHFVDHCIEILSHDKDYNKVKLYEHAVAHSGLPDNARR   226
rsKiiro         EKGVKLPGAHFVDHCIEILSHDKDYNKVKLYEHAVAHSGLPDNARR   226
skylan-NS       EKGVKLPGAHFVDHCIEILSHDKDYNKVKLYEHAVAHSGLPDNARR   226
                ******** *************************************
```

Supplementary Fig. 51. Multiple sequence alignment for the rsKiiro, skylan-NS and EosFP sequences.

Improved photoproduct quantum yield is critical for femtosecond TR-SFX studies due to the temporal scales of the experiment being shorter the excited state life of these systems (~50 ps, see below). Excitation events that follow non-photoreaction electronic pathways which return to the ground state of

the system have no opportunity to be re-excited and contribute to the total photoactive population. Therefore the theoretical maximum population that could be obtained by femtosecond excitation is capped at the photoproduct quantum yield (see below). This is not usually achievable as other excitation pathways such as excited state absorption leading to double excitation and stimulated emission will occur with strong optical pumping preventing a 100% occupancy of the S_1 electronic state. Despite improvements in TR-SFX data processing it is still not possible to reliably detect photoinduced differences of populations below ~10% level and the feasibility of a femtosecond TR-SFX experiment can hinge on the quantum yields of the sample. The situation is more relaxed with longer nanosecond timescale excitation where multiple re-excitations events can occur within the duration the pulse enabling final populations in excess of photoproduct quantum yield.

As previously mentioned the population differences are small and light induced difference on such short timescales can be incredibly subtle therefore maximizing crystallographic resolution is very important, the improvements made in rsKiiro meant that at room temperature microcrystal SFX structures collected at SACLA and LCLS XFELs routinely reach resolutions below <1.5 Å. This was an improvement from the 1.8 Å previously measured on Skylan-NS microcrystals. Depending on sample delivery method XFEL beamtimes have very demanding sample consumption particularly with optically driven reactions where the use of a grease injector is not desirable due to the potential for light-piping up the jet coupled with high quantum yield causing accumulated photo conversion in upstream crystals reducing the potential signal to noise obtainable in PP measurements. The GDVN consumed >5g of protein during LR23.

Screening for expression yield was quickly determined during initial grow ups. Due to the mutations being mostly limited to the chromophore region and crystallization conditions of the parent molecules being readily available in the literature obtaining initial crystals for testing was usually straight forward. These batch grown microcrystals used for the screening photoproduct quantum yield by performing femtosecond flash photolysis measurements on crystalline “pancakes” as described in the main text, both yield and non-recoverable bleaching were screening factors. Finally cryo-MX structures were collected in both the ground state and pre-illuminated crystals to confirm that crystal order was not disrupted through photo-activation.

rsKiiro has been selected for the specific photochemical characteristics described in the manuscript, that include the action of the double well potentials for both the off and the on states and the complete 100% photoswitching between on and off states. Additional characteristics considered for its selection included the photochemical quantum yield of photoisomerisation, the excited state lifetime which exceeds the vibrational dephasing time, and the activated barrier crossing and incoherent reaction pathway for photoisomerisation. The sequence is based on the EosFP gene from *Lobophyllia hemprichii* and the orthorhombic crystal form was found to diffract to high resolution in both the dark and the illuminated states. The expression and crystallisation characteristics were additionally excellent. These characteristics of rsKiiro allowed the investigation of the coherence contributions on ultrafast timescale as described. These are also fully distinct and different from other photoswitching FPs described in the literature. These include rsEGFP2[30,31], which is based on the *Aequorea Victoria gfp* sequence, and irisFP[32], that both have very different photochemical properties and structures.

11. Thermodynamic modelling of rsKiiro photo-switching

11.1. Analytical solution to the overall off → on conversion process using the Master Rate Equation method.

The temperature dependence of the off → on photoisomerization reaction takes the primary evidence from the femtosecond time resolved SFX crystal structure that identifies the short lived A_2 structure that is not populated at room temperature in equilibrium conditions. The proposed model for the off → on reaction is shown in Extended Data Fig. 1g & Supplementary Fig. 52 .

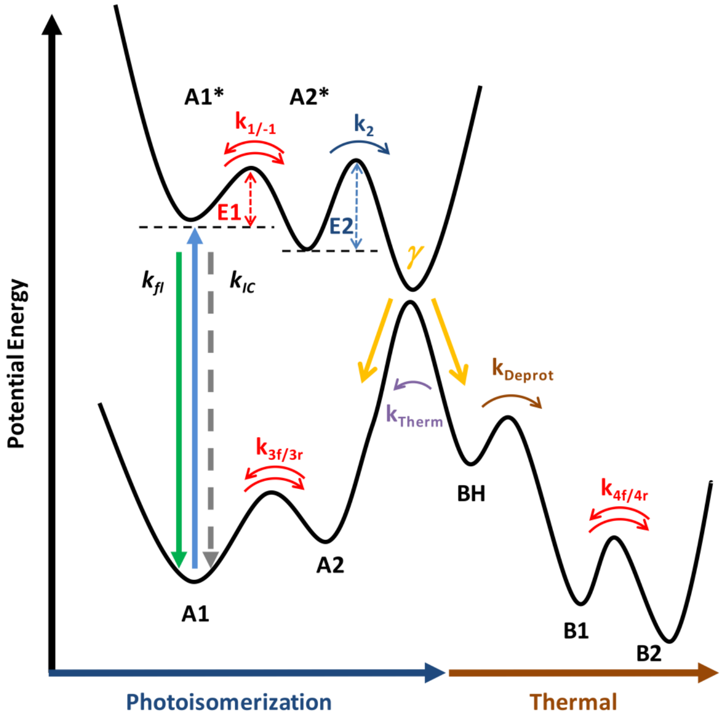

Supplementary Fig. 52 | Proposed electronic structure model for rsKiiro off→on photoreaction.

In the Born-Oppenheimer approximation there will exist both A_1^* and A_2^* coordinates with a singlet excited state electronic structure. We thus consider two separate barriers E_1 and E_2

$$
\begin{gathered}
A_1 \xrightarrow{k_{exc}} A_1^* \\
A_1^* \underset{k_{1r}}{\overset{k_{1f}}{\rightleftarrows}} A_2^* \\
A_2^* \xrightarrow{\gamma k_2} BH \\
A_2^* \xrightarrow{(1-\gamma)k_2} A_2 \\
A_2 \underset{k_{3r}}{\overset{k_{3f}}{\rightleftarrows}} A_1 \\
BH \xrightarrow{k_{Deprot}} B_1 \\
BH \xrightarrow{k_{Therm}} A_2 \\
B_1 \underset{k_{4r}}{\overset{k_{4f}}{\rightleftarrows}} B_2
\end{gathered}
\tag{S4}
$$

$$A_2^* \xrightarrow{k_{fl}} A_2$$
$$A_1 \xrightarrow{k_{relax(A-B)}} B_2$$

Using the master equation method[33,34] the time dependence is given by

$$\frac{dx}{dt} = K_c x \qquad \text{(S5)}$$

Or

$$\frac{dx}{dt} = -\sum_{j=1}^{m} K_{ij}x_i + \sum_{j=1}^{m} K_{ji}a_j (i = 1,2,..,m, i \neq j) \qquad \text{(S6)}$$

There x is a vector of concentrations of species, and $K_c x$ is given as:

$$\begin{bmatrix} -k_{exc}-k_{relax}-k_{3r} & 0 & 0 & k_{3f} & 0 & 0 & 0 \\ \mathrm{k_{exc}} & -k_{1f} & k_{1r} & 0 & 0 & 0 & 0 \\ 0 & k_{1f} & -k_{1r}-\gamma k_2-(1-\gamma)k_2-k_{fl} & 0 & 0 & 0 & 0 \\ k_{3r} & 0 & k_{fl}+(1-\gamma)k_2 & -k_{3f} & k_{Therm} & 0 & 0 \\ 0 & 0 & \gamma k_2 & 0 & -k_{Therm}-k_{Deprot} & 0 & 0 \\ 0 & 0 & 0 & 0 & k_{Deprot} & -k_{4f} & k_{4r} \\ k_{relax} & 0 & 0 & 0 & 0 & k_{4f} & -k_{4r} \end{bmatrix} \times \begin{bmatrix} A_1 \\ A_1^* \\ A_2^* \\ A_2 \\ BH \\ B_1 \\ B_2 \end{bmatrix} \qquad \text{(S7)}$$

Diagonalization of Kc

$$K_c \xi = \lambda \xi$$

solves the master equation analytically to provide the eigenvalues λ(n) alternatively these may be solved using MATLAB's symbolic methods.

```
syms kexc krelax k3f k1f k1r gamma k2 kfl k3r ktherm kdeprot k4f k4r

A = sym([(-kexc-krelax-k3r) 0 0 k3f 0 0 0; kexc -k1f k1r 0 0 0 0; 0 k1f (-k1r-(gamma*k2)-(1-gamma)*k2-kfl) 0 0 0 0; k3r 0 (kfl+(1-gamma)*k2) -k3f ktherm 0 0; 0 0 (gamma*k2) 0 (-ktherm-kdeprot) 0 0; 0 0 0 0 kdeprot -k4f k4r;krelax 0 0 0 0 k4f -k4r])

[V]=eig(A)
```

Box: matlab code to compute eigenvalues using symbolic function

The dimensions of the computed eigenvalue functions, which are not printed here, are 429 terms.

This in principle provides solutions to the master equation

$$x = PLP^{-1}x_0 \qquad \text{(S8)}$$

Where $P = [\xi_1, .., .., \xi_n]$is the matrix whose i[th] column is the eigenvector ξ_i and

$$L = \begin{bmatrix} \exp(\lambda_1 t) & 0 & 0 & 0 \\ 0 & \exp(\lambda_1 t) & 0 & 0 \\ 0 & 0 & \ddots & \vdots \\ 0 & 0 & \cdots & \exp(\lambda_n t) \end{bmatrix} \qquad \text{(S9)}$$

Alternatively solutions may be found from 'Sylvester's formula'[34] which depend on the eigenvalues only and do not need the eigenvectors

$$x = \exp(Kt)\, x_0 \tag{S10}$$

$$\exp(Kt) = \sum_{i=1}^{n} M_i \exp(\lambda_i t) \tag{S11}$$

$$M_i = \prod_{\substack{j=1 \\ j \neq i}}^{n} (K - \lambda_j I) / \prod_{\substack{j=1 \\ j \neq i}}^{n} (\lambda_i - \lambda_j) \tag{S12}$$

This provides analytical solutions to the coupled differential equation. In practice the system could be solved in more straightforward manner using numerical approach, which however requires that all parameters are known. Therefore, the steady state approximation reduces the complexity of an analytical approach.

Similarly, the photoisomerization of the B_1 state to produce the relaxed A_1 photoproduct is directly equivalent but lacks the final relaxation as the B_1 state is the dark resting species. Furthermore, the thermal protonation follows the cis-trans photoisomerization (Supplementary Fig. 52). However this manuscript does not include experimental observation of these processes directly and also does not contain measurement of the explicit rates.

11.2. Summary of theoretical considerations for temperature dependence of the trans-cis photoisomerization

It has previously been shown that the temperature dependence of fluorescence and internal conversion is expected to be small[35], summarized below. The fluorescence rate can be considered to relatively independent of temperature because the absorption and fluorescence spectra do not change significantly over the temperature range that is studied. Therefore the Strickler-Berg relation predicts the rate not to change significantly. The temperature dependence of internal conversion is known from theory and is argued to be weak for fluorescent proteins on the basis of the following arguments previously put forward[35]:

The internal conversion rate constant has a temperature dependent and a temperature independent part

$$k_{IC} = k_{IC}^{0}(T_{independent}) + k_{IC}(T_{dependent}) = k_{IC}^{0} + k_{IC}^{T} \exp\left(-\frac{E_A}{RT}\right) \tag{S13}$$

Fluorescence and internal conversion can be described by the "spin-boson Hamiltonian", in which "spin" is a two-level system (e.g. electronic ground and excited states), and "boson" is a bath of harmonic oscillators that couple to the two-level system to various degrees [36,37]

$$H = \Sigma_{n=g,e}(E_n + \Sigma_\alpha g_{n\alpha} x_\alpha)|n\rangle\langle n| + V_{g,e}|g\rangle\langle e| + V_{e,g}|e\rangle\langle g| + \Sigma_\alpha \left(\frac{p_\alpha^2}{2m_\alpha} + \frac{1}{2} m_\alpha \omega_\alpha^2 x_\alpha^2\right) \tag{S14}$$

where $|g\rangle$ and $|e\rangle$ are the electronic ground and excited states, E_n are the corresponding electronic energies without any couplings, α are the harmonic bath normal modes, x_α and p_α are their displacements and momenta, m_α and ω_α are their masses and frequencies, $V_{g,e}$ and $V_{e,g}$ are the electronic couplings between two electronic states, and $g_{n\alpha}$ are the couplings between the bath modes and the electronic states. The interpretation is that fluorescence and internal conversion between two electronic energy levels are a competition between photons and phonons governed by their relative coupling strengths.

Fermi's golden rule expresses the internal conversion rate

$$k_{IC} = \frac{\left|V_{eg}\right|^2}{\hbar^2} e^{-\Sigma_\alpha S_\alpha (2n_\alpha+1)} \int_{-\infty}^{\infty} dt\; e^{i\omega_{eg}t + \Sigma_\alpha S_\alpha \left(n_\alpha e^{i\omega_\alpha t} + (n_\alpha+1)e^{-i\omega_\alpha t}\right)} \tag{S15}$$

where S_α are dimensionless quantities, the Huang-Rhys factors, which are directly related to the vibronic coupling strengths. Further detail is found in references[35–37] that discuss two possible regimes that differ in the coupling strengths. If the vibronic couplings are weak it is shown that $k_{IC} \sim e^{-\omega_{eg}}$ and is thus dominated by the energy gap, which is known as the Englman-Jortner energy-gap law. In the opposite regime of strong coupling

$$k_{IC} = \frac{\left|v_{e,g}\right|^2}{\hbar} \sqrt{\frac{\pi}{k_B T E_r}} e^{-\frac{E_A}{k_B T}} \tag{S16}$$

With

$$E_A \equiv \frac{\left(E_{eg} - E_r\right)}{4E_r} \tag{S17}$$

The temperature dependence of k_{IC} therefore depends on the magnitude of the Huang-Rhys factors. Lin et al.[35] previously argued that for fluorescent proteins computational results from Bochenkova et al. could be used[38]. From that data Lin et al estimated the summation. $\Sigma_\alpha S_\alpha n_\alpha$ to be approximately 0.5 which would be well below the expected value where temperature dependence would be significant. The same authors Lin et al.[35] carried on to evaluate theoretical arguments that show weak temperature of the branching ratio γ. Using the Launau-Zener theory[36,39,40]

$$\gamma = \exp\left(-\frac{2\pi|V_{12}|^2}{\hbar v |F_1 - F_2|}\right) \tag{S18}$$

Where V_{12} is the coupling between two surfaces, V is the nuclear velocity approaching the intersection, and F_1 and F_2 are the slopes (R is the nuclear position along the reaction coordinate) of the corresponding surfaces at the intersection. Lin et al argued that the Franck-Condon excitation is weakly temperature dependent, such that the nuclear velocity is proportional to $v \sim \sqrt{T}$ which is weaker than Arrhenius behaviour.

11.3. Modelling of the temperature dependence of cis-trans and trans-cis photoisomerization

The following considerations inform a model for the analysis of the temperature dependence of photoisomerization of the rsKiiro fluorescent protein. Firstly we analyse the cis-trans photoisomerization, as we have available measurements of the temperature dependence of both the fluorescence decay (Extended Data Fig. 1i) as well as the photoisomerization rate (Extended Data Fig. 1e & k).

The overall fraction of the fraction of B_1* converted to the A state is

$$\varphi_{overall} = \frac{\frac{k_{1f}}{k_{1r}} k_2}{k_f + k_{IC} + \frac{k_{1f}}{k_{1r}} k_2} \times \gamma \tag{S19}$$

The overall temperature dependence of photoisomerization from the conventional Arrhenius equation is

$$\varphi_{overall}(T) = \frac{\frac{A_{1f}}{A_{1r}} A_2 \exp\left(-\frac{E_{1f} - E_{1r} + E_2}{RT}\right)}{k_f + k_{IC} + \frac{k_{1f}}{k_{1r}} k_2} \times \gamma \quad \text{(S20)}$$

For both the on→off and off→on photoisomerization the fluorescence contributes only very little to the excited state decay (6% and 0.5 % respectively). In both cases the internal conversion will dominate the excited state decay. As previously discussed, the temperature dependence of the internal conversion rate depends on the magnitude of the Huang-Rhys factors. We are therefore able to compare the temperature dependence of the fluorescence, which measures all the contributions to the excited state decay, with the temperature dependence of the photoisomerization rate (k_{PI}). The total rate (k_T) from all contributions can be decomposed as:

$$k_T = k_{PI} + k_{IC} + k_R \quad \text{(S21)}$$

And further expressed as

$$k_T = \gamma_{PI} k_T Q_{PI} + \gamma_{IC} k_T Q_{IC} + \gamma_R k_{T(300k)} \quad \text{(S22)}$$

Where γ_n and Q_n are the quantum yields and temperature scalings of each process respectively.

For the on→off reaction γ_{PI} was found to be 0.63% from CW illumination of solution samples (see below) and Q_{Ph} can be determined using the E_{PI} = 2.0±0.4 kJ mol^{-1} from Arrhenius measurements (Extended Data Fig. 1k) and normalizing to the 300 K rate. By subtracting the scaled temperature dependent contributions due to the photoisomerization rate, it is possible to fit the Arrhenius equation to the remaining temperature dependent rate and obtain and effective barrier for the internal conversion processes (Extended Data Fig. 1l) of E_{IC} =16.5 ± 0.6 kJ mol^{-1}.The third term in (S22), the radiative rate, is assumed to be temperature independent and therefore will not contribute to the gradient in the Arrhenius plots.

We can perform the same calculation for the off→on reaction using the temperature dependence of the excited state decay obtained from transient absorption spectra (Extended Data Fig. 1d). E_{PI}= 3.5 ±1.2 kJ mol^{-1} (Extended Data Fig. 1e) & γ_{PI}= 15.9±1.2% (Supplementary section 6). The final recovered E_{IC} = 16.51 ± 0.53 kJ mol^{-1} (Extended Data Fig. 1f)

All the barriers recovered from these fittings are shown in Extended Data Fig. 1h. It can be seen that there very similar barriers obtained for E_{IC} in both the on→off and off→on reactions while the E_{PI} barrier is approximately twice as large for the off→on reaction.

Nevertheless, the temperature dependence of photoisomerization is dominated by the excited state pre-equilibrium between A_1* and A_2* states in combination with an excited state barrier.

$$A_1^* \underset{k_{1r}}{\overset{k_{1f}}{\rightleftarrows}} A_2^* \overset{k_2}{\rightarrow} BH \quad \text{(S23)}$$

For this process the overall rate becomes

$$k_{overall} \sim \frac{A_{1f}}{A_{1r}} A_2 \exp\left(-\frac{E_{1f} - E_{1r} + E_2}{RT}\right) \quad \text{(S24)}$$

Therefore the Arrhenius pre-factor for the high-temperature regime measured for both on→off and off→on directions is $\frac{A_{1f}}{A_{1r}} A_2$ while the effective activation energy is $E_{1f} - E_{1r} + E_2$. It is worth noting

that such re-equilibrium can lead to a effective negative activation energy which[41–43] has also been reported for the analysis of fluorescence decay of the Green Fluorescent Protein (GFP)[44].

11.4. Ground state evolution in the off → on direction

With excitation of the A_1 ground state, with neutral trans chromophore in relaxed geometry, the macroscopic absorption differences at room temperature shows an isosbestic point and are assigned to the conversion of A_1 to produce the final B_2 state with anionic cis chromophore structure. The ground state species evolution for the trans-neutral off state to the cis anionic on state chromophore

$$A_1 \underset{\overleftarrow{k_r}}{\xrightarrow{k_f, k_{therm}}} BH \xrightarrow[k_{Deprot}]{} B_1 \xrightarrow[k_{relax}]{} B_2 \qquad \text{(S25)}$$

The forward rate constant k_f is proportional to the optical excitation rate and the primary quantum yield, and at room temperature exceeds the thermal rate constant k_{therm}=1.7x10^{-3} s^{-1} by several orders of magnitude which can be neglected. The reverse rate constant k_r is similarly proportional to the optical excitation rate and the primary quantum yield and can be neglected since the deprotonation rate k_{Deprot}= 1/40 μs = 1.5x10^4 s^{-1} far exceeds the optical excitation rate. Subsequent to deprotonation of the cis neutral chromophore photoproduct, a relaxation of the B_1 product to form the folly relaxed B_2 product with rate k_{Relax} is assumed from the crystallographic observation that traps the B_1 state at low temperature. Since the B_2 state was observed at room temperature at the 100 μs time point following femtosecond excitation of A_1, the value of k_{Relax} at room temperature is higher than ~2x10^4 s^{-1}. Therefore the overall ground state process at room temperature is well described with the two-state forward phototransfer.

$$A_1 \xrightarrow{\varphi, k_f} B_2 \qquad \text{(S26)}$$

With

$$\frac{d[A_1]}{dt} = \frac{P_0}{V}(1 - 10^{-A_t})\left(-\frac{\epsilon_{A_1} l[A_1]}{A_t}\varphi\right) \qquad \text{(S27)}$$

With P_0 the incident light power, l the path length, V the volume and A_t the total absorbance. For the dilute limit taking the first term in the Taylor expansion allows the approximation

$$k_{overall} = \frac{d[A_1]}{dt} = \frac{P_0}{V}\ln 10\ \left(\epsilon_{A_1} l[A_1]\right)\varphi_{off \to on} \qquad \text{(S28)}$$

Similarly, for the photoconversion of the on state with cis anion chromophore to the relaxed off state, the overall process for ground state evolution is (not showing thermal relaxation)

$$B_2 \underset{\overleftarrow{k_r}}{\overset{k_f}{\rightarrow}} A^- \xrightarrow[k_{Prot}]{} A \xrightarrow[k_{relax}]{?} A_1 \qquad \text{(S29)}$$

The possible intermediate and relaxation in this direction of conversion is implied from cryotrapping (Supplementary Fig. 48 - Supplementary Fig. 50). With the observation of isosbestic point the overall process is described as

$$B_2 \underset{\xleftarrow[k_{therm}]{}}{\overset{\varphi k_f}{\longrightarrow}} A_1 \qquad \text{(S30)}$$

Neglecting the contribution of k_{therm} the overall rate for the on-to-off conversion is

$$k_{overall} = \frac{d[B_2]}{dt} = \frac{P_0}{V}\ ln10\ (\epsilon_{B_2} l[B_2])\varphi_{on\rightarrow off} \tag{S31}$$

11.5. Modelling of the convex Arrhenius behaviour of the on→off and off→on photoconversion

In contrast to recently reported temperature dependence of fluorescence of fluorescent proteins that scanned a relatively small range of temperatures near ambient values (279 - 315 K), here we show that the kinetics of photo-conversion measured for lower temperatures significantly deviate from the high temperature regime. A few µL of rsKiiro (60mg/mL) solution mixed with 100% glycerol cryoprotectant in a 30:70 ratio was loaded onto a 20 µm CryoLoop™ (Hampton) to give a final OD @ 490 nm of 0.2-0.3. The absorption was monitored using a fiber based spectrometer linked to a microscope and the temperature was controlled using a cryo stream (Oxford instruments) positioned ~1 cm from the loop. The forward on→off reaction was driven by an unfocused 15 mW 473 nm CW laser, while the reverse reaction was driven by an unfocused 5 mW 400 nm CW laser. For both directions a convex Arrhenius behaviour is observed (Extended Data Fig. 1e & k), separate analysis of the linear regions therefore retrieves larger activation energies as well as larger pre-factors for the low temperature regime.

We note that the transition temperature of the kinetic series in Extended Data Fig. 1e & k are 229±2 K, 208±4 K, 236±10 K for the on→off, off→on with low temp and room temp preconversion conditions respectively. The usual interpretation of kinetic transitions in this temperature range is the glass transition in proteins[45]. An empirical approach to model a transition temperature writes the rate coefficient approximated by the Vogel-Tammann-Fulcher (VTF) relation

$$k_\alpha(T) = A_\alpha \exp\left(-\frac{DT_0}{T - T_0}\right) \tag{S32}$$

Where T_0 is an experimentally determined transition temperature and the amplitudes A_α and D are empirical from experiment. The rate is taken to depend inversely proportional to the viscosity in the mechanical Maxwell relation[45].

While the viscosity changes at the glass transition cannot be excluded we consider as primary evidence that thermal relaxation of the metastable off state significantly increases the value of the transition temperature for the convex Arrhenius plot for the off-on transition (Extended Data Fig. 1e & k). Therefore, we model this dominating relaxation behaviour as a Gibbs Free Energy difference ΔG between relaxed and strained conformations of the excited state intermediate E_2^*. This model directly connects the ultrafast SFX measurements and structural determination of the chromophore and hydrogen bonding dynamics with the thermodynamics that describe the temperature dependence of photoisomerization.

For an equilibrium that models a temperature dependence of an intermediate A_1^* / A_2^* with a $K_{eq} = k_f/k_r$, the Gibbs Free energy is $\Delta G = -RT \ln K_{eq}$

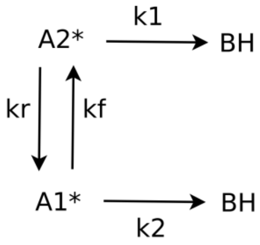

Here we treat the observed behaviour as a switching between two distinct confirmations each with their own reaction pathways that undergo a transition between the active and inactive at the point of the break. The rate constants k_1 and k_2 dominate in the high and low temperature region so that the overall rate k_{all} is

$$k_{all} = \frac{k_1}{1+\exp\left(+\frac{\Delta G}{RT}\right)} + \frac{k_2}{1+\exp\left(-\frac{\Delta G}{RT}\right)} \quad \text{(S33)}$$

Where

$$k_1 = \mathcal{A}_1 \exp\left(-\frac{E_{T1}}{RT}\right)$$

$$k_2 = \mathcal{A}_2 \exp\left(-\frac{E_{T2}}{RT}\right)$$

And

$$\Delta G = \Delta H - T\Delta S$$

$\mathcal{A}_n$ and E_{Tn} are the Arrhenius prefactors and total activation energy for each of the reactions, ΔH is the change in entropy and ΔS the enthalpy. By fixing the values for E_{T1} and E_{T2} to the same as the local (linear) fits to the Arrhenius equation, allows the values ΔH and ΔS to be determined from the steepness and location of the Arrhenius break. In order to produce the convex behaviour it is necessary to incorporate an Enthalpy-Entropy compensation such that the sign of ΔG changes across the transition temperature. The break position (T_k) the point at which $\Delta G = 0$. This uses a modified expressions previously developed by Klinman et. al to model convex Arrhenius behaviour in enzymology [46].

Supplementary Table. 10. Arrhenius fitting of rsKiiro photoswitching reactions

Reaction	E_{T1} (kJ/mol)	E_{T2} (kJ/mol)	$\mathcal{A}_1$ (s^{-1})	$\mathcal{A}_2$ (s^{-1})	ΔH (kJ mol^{-1})	ΔS (kJ mol^{-1} K^{-1})	T_t (K)
on – off	2±0.4	32±3	1.5±0.7	32±5	66±4	0.29±0.02	229±2
off- on (Low temp precon.)	3.5±1.2	40±4	0.9±1.2	22±3	51±20	0.24±0.1	208±4
off – on (room temp precon.)	-	55±20	-	26±10	58±25	0.25±0.13	236±10

Practically for the purposes of fitting the modification to k_1 and k_2 produces a sigmoid-like switching behaviour such that k_1 dominates at high temperature and k_2 low temperature. The temperature at which the transition occurs depends on the ratio of H to S while curvature of the break is inversely proportional

to H, we therefore substitute S for H/T_k. The sections above and below the Arrhenius break were independently linearly fit (Extended Data Fig. 1e & k, dashed lines) using a similar bootstrap method to that described in supplementary of Romei et al.[13] to obtain values of A_n and E_n (Supplementary Table. 10) for the high and low temperature regions. For fitting the full range of temperatures the activation energies and prefactors obtained from the linear fits were used in Eqn. (S33) and the only free parameters were H and T_k . It was necessary to relax constraints on the value of the A_2 to secure a good fit, we justify this by noting that due to the limited number of data points available the linear fits there will still be contributions from the other confirmer. Due to the similarity between the high temperature behaviour of the two off→on conditions and the limited number of data points for the room temperature preconversion the linear fit of the cryo preconversion was used to fit both datasets, we justify this as the difference between them is attributed to cryo temperatures modifying the reaction pathway therefore these data sets must converge at room temperature. We observe very similar values for the H for all three conditions, due to the larger error in the cryo on→off dataset at the transition temperature it is possible force the fit to a very large value (i.e H >500 kJ/mol) we disregard this as aphysical.

11.6. Explicit thermodynamics treatment of barriers in the reactive coordinate and non-radiative transitions

In order to derive the functional significance of the double-well adiabatic potential discovered in our ultrafast crystallography experiments we combined the structural information with kinetics measurements and thermodynamics modelling. The double-well adiabatic nature of both the off and on states was additionally shown from the thermodynamic analysis of reversible photoisomerisation as well as the excited state decay. We thus analyses and quantitatively modelled the temperature dependence of the radiative and non-radiative transitions of both the on and off states (Extended Data Figure 9) (Section 11 of the Supplementary materials).

The temperature dependence of both the *trans-cis* and *cis-trans* photoisomerization shows convex non-Arrhenius kinetics in which the transition temperature is additionally sensitive to structural annealing which lowers the ‘A*2’ potential level (Fig. 5). Convex Arrhenius kinetics are conventionally analysed using parameters for double-well potentials. Modelling of the non-Arrhenius kinetics invoked an Entropy-Enthalpy compensation scheme and retrieved the relaxation parameters of the second energy level of the double-well potential (Fig. 2, 5, A1*, A2*, Extended Data Fig. 1e & k and section 11.5 of supplementary materials). A full thermodynamics treatment of photoisomerization employs the knowledge of the double-well potential (Extended Data Fig. 1g) and uses the experimental separation of the radiative and non-radiative transitions that showed different barrier values (Extended Data Fig. 1f & l). We determined the temperature dependence of the excited state decay of the off state from stimulated emission decay kinetics (Extended Data Fig. 1d), and on state from fluorescence measurements (Extended Data Fig. 1i &-j) for samples in solution. We find that the internal conversion of the *trans* off state encounters a larger barrier at 17.0±0.6 kJ mol^{-1} than that found for the effective barrier $E_{total} = E_{1forward} - E_{1reverse} + E_2$ = 14.1±0.7 kJ mol^{-1} for photoisomerization. Similarly, the internal conversion of the *cis* state encounters a larger barrier at 16.5±0.6 kJ mol^{-1} than that found for the effective barrier $E_{total} = E_{1forward} - E_{1reverse} + E_2$ = 12.7±1.1 kJ mol^{-1} for photoisomerization.

Our treatment addresses the thermodynamics of the total excited state decay, $k_{total} = k_{pc} + k_{IC} + k_{fl}$ and evaluates the magnitude of the barriers in light of theoretical considerations, known as Landau-Zener theory [40] and Engleman-Jortner’s approach[47] to internal conversion (see section 11.2 of

supplementary materials for details). We conclude from the apparent barriers in the reactive and non-radiative pathways that internal conversion is dominated by a free rotor motion in combination with low frequency motion, which includes a combination of torsional and coupling coordinates. This model adheres to what is known as the Seidner-Domcke formalism[48]

The involvement of the double-well potential (Fig. 2) in the photoisomerization pathway was investigated from the details of the temperature dependence of kinetics (Fig 5 & Extended Data Fig. 1). In order to derive the functional significance of the double-well potential discovered from our ultrafast crystallography experiments we take advantage of the structural knowledge together with thermodynamics and kinetics information that is otherwise not accessible for explicit modelling. Both the cis(A)-trans(B) and trans-cis photoconversion showed relatively small temperature sensitivity near ambient temperatures (Fig. 5) An Arrhenius fit of the high temperature (293 K – 250 K) region retrieved values for the activation energies of 2±0.4 and 3.5±1.2 kJ/mol respectively (Fig. 5). A further lowering of the temperature dramatically reduced the kinetics in both directions resulting in convex Arrhenius behaviour (Fig 5 & Extended Data Fig. 1e,k). The modelling of temperature dependence of double-well potentials, notably involving hydrogen bonding modification, is well established and is known to result in reduced or negative activation energy since the effective barrier $E_{Total} = E_{1forward} - E_{1reverse} + E_2$ determines the overall temperature dependence and the pre-factors become the resulting combination[41,42,44,49] and will show an apparent underestimation of the apparent attempt frequency (see above). Interestingly a negative activation barrier of -5 kJ mol^{-1} was observed for the proton transfer in the Green Fluorescent Protein and modelled with a double well potential[44]. Significantly larger activation energy and pre-factors are shown for both cis-trans and trans-cis reactions (Extended Data Fig. 1h). Cryo-trapping of photoproducts after illumination at temperatures that are in low and high temperature regimes of the convex Arrhenius behaviour confirmed that cis-trans- and trans-cis photoisomerization is maintained in all cases (see above). The transition temperatures were 229 K and 208 K for cis-trans and trans-cis directions (Fig. 5). The conventional interpretation invokes the glass transition proposed by Marcus[50]. However we found that pre-converting to the trans (Extended Data Fig. 1e) state at room temperature both significantly reduced the subsequent conversion rate as well as shifting the transition temperature to higher value (236 K, Fig. 5, Right, blue series) as compared to pre-conversion at cryo temperatures. This key observation argues against the conventional glass transition models proposed for similar transition temperatures of kinetics. We therefore developed a model that involves the relaxation of the secondary, intermediate, well for both directions using a single Gibbs free energy difference. To arrive at the convex Arrhenius behaviour the fractional occupancies of intermediate states follow the enthalpy-entropy compensation such that $\Delta H = T_T \Delta S$ where T_T is the transition temperature (see above). The thermodynamics therefore connect the chemical structures with the double-well behaviour. The PDP experiment resolved the A_2 structure, whereas cryo-trapping resolved the unrelaxed B_1 for the cis state, as shown by hydrogen bonding rearrangements and a positional change of the chromophore (F_o-F_o difference map shown in Fig. 5, and see below).

Finally, we show the functional importance and physical significance of the ultrafast crystal structures and the wavepacket dynamics in the double well description for the photoisomerization reaction pathway. Specifically we connect the double well potential behaviour with the thermodynamics and observed non-Arrhenius behaviour of the photoswitching kinetics. This analysis requires precise knowledge of non-radiative transitions and the full temperature dependence of competing channels of

photochemical dynamics. The cis-trans and trans-cis photoisomerization of the fluorescent protein chromophore includes ultrafast barrier crossing and avoidance of internal conversion processes with barriers 16.5 ±0.6 & 17.0 ±0.6 kJ mol^{-1}respectively (Extended Data Fig. 1h)

We measured the temperature dependence of the fluorescence of the cis chromophore in the ON state in the high temperature region using TCSPC (7). The fluorescence was biphasic with lifetimes of 170 ps and 600 ps (@ 294 K), for which similar activation energies of 3.5 ± 0.9 and 4.0 ± 0.2 kcal mol^{-1} were determined. The statistical analysis showed these activation energies exceeded that determined for photoconversion. For analysis of the contributions to the total rate of excited state decay, $k_{total} = k_{pc} + k_{IC} + k_{ISC} + k_{fl}$ the fluorescence can be neglected at a quantum yield of 6.8 ± 0.1% and 0.49 ± 0.03% (8) for the cis and trans chromophores respectively and ISC is similarly neglected. The dominant contributions to decay are photoisomerization (k_{pc}) and internal conversion (k_{IC}). Using the Landau-Zener formulism the branching ratio at the conical intersection is found to be proportional to $\propto e^{-1/2}\sqrt{T}$ via the temperature dependence of the nuclear velocity neglecting non-equilibrium effects on the Franck-Condon excitation[35,36,40]. The Landau-Zener equation therefore predicts that the temperature dependence of a small range can be neglected. The theoretical considerations for the temperature dependence of internal conversion are previously considered specific for fluorescent proteins and assumed to be weakly temperature dependent[35]. Neglecting a temperature independent zero-point motion tunnelling contribution due to the large mass involved in transfer, the Englman-Jortner approach[36,47,51] in the weak coupling regime predicts from the energy gap law that temperature dependence is weak. Also in the strong coupling limit an activated barrier crossing behaviour is retrieved that predicts weak temperature dependence and is proportional to $\propto e^{\frac{(E_{ge}-E_r)^2}{4E_r}}$ where E_{ge} and E_r are the electronic and relaxation energies. The experimental temperature dependence of fluorescence was analysed by applying the measured quantum yields of photoconversion and internal conversion, and in this manner retrieved the activation energy for E_{IC} = 16.5 ± 0.6 kJ mol^{-1} (11.3). The lowest energy solution to strong coupling term for the observed 16.5 kJ mol^{-1} barrier to internal conversion for the cis state would therefore need a 11,683 cm^{-1} relaxation energy which disfavours a strong vibronic coupling mechanism for internal conversion, following Englman-Jortner. Internal conversion is therefore considered to be dominated by a free rotor motion in combination with low frequency motion, which includes a combination of torsional and coupling coordinates in Seidner-Domcke formalism[48]. This differs from the behaviour of the isolated p-hydroxybenzylidene imidazolidinone (HBDI) chromophore in solution, for which near-barrierless internal conversion was seen at high temperatures[52]. Here we have shown that the internal conversion of the cis state encounters a larger barrier at 16.5 kJ mol^{-1} than that found for the effective barrier $E_{total} = E_{1forward} - E_{1reverse} + E_2$ = 2 kJ mol^{-1} for photoisomerization. Since spectroscopy did not find a separation of time scales within the excited state decay for the trans chromophore, a further branching in Seidner-Domcke potential separates the paths in additional coordinates beyond the model shown (Extended Data Fig. 1g & Supplementary Fig. 52). Therefore, our full analysis finds the thermodynamics and barriers of non-radiative transitions separate from photoisomerization and extends the detailed and complete thermodynamics modelling of structural dynamics of all origins on ultrafast time scales of the rsKiiro fluorescent protein.

In conclusion, we find for both the trans off state and the cis on state that the internal conversion encounters a larger barrier than that found for the effective barrier for photoisomerization. In addition,

we find a relaxation mechanism within the double well potential that results in convex Arrhenius plots for the photoisomerization reaction of both the on and off states. We also determined crystal structures for cryo-trapped intermediates in the double well potential of the on state, shown in Extended Data Figure 9. We present an unprecedented opportunity to use knowledge of ultrafast crystallography to explain and quantitatively model the thermodynamics of functional reactions, in this case the photoisomerization coordinates in both forward and reverse directions.

12. Hybrid Quantum Mechanics / Molecular Mechanics Computations

The main aim of the computations is to test the conjecture that there are two configurations, corresponding the A_1 and A_2 minima (Fig. 2), separated by a small barrier on both the electronic ground state (S_0) and excited state (S_1) potential energy surfaces. We therefore constructed initial models for A_1 and A_2 based on the differences between the resting state structure and the pump-probe structure. In the A_1 configuration (Extended Data Fig. 8a), the hydroxyl group of the chromophore donates a hydrogen bond to a water molecule, which donates hydrogen bonds to the carboxylate side chain of Glu144 and the backbone carbonyl of Gly155. In the A_2 configuration (Extended Data Fig. 8a), the hydroxyl group of the chromophore forms a direct hydrogen bond with the side chain of Glu144, while the water molecule donates a hydrogen bond to the chromophore hydroxyl.

The initial structure for these two models was the resting state X-ray structure of rsKiiro. For amino acids resolved in multiple conformations, we selected A-conformation, with the exception of His194, for which the B conformer was selected that can form additional hydrogen bonds to stabilize the chromophore pocket. In addition, the B-conformation of His194 also has a higher population in the crystal. All other missing hydrogens atoms were added with the GROMACS pdb2gmx tool. To model the interactions, we used the Amber03 Molecular Mechanics (MM) forcefield[53]. The missing chromophore parameters were obtained using the AnteChamber protocol [54].

Geometry optimizations were performed at both the MM level and the hybrid quantum mechanics / molecular mechanics (QM/MM) level with Gromacs version 4.5.5[55], in combination with the TeraChem quantum chemistry program[56]. This Gromacs/TeraChem QM/MM interface is available for download from Github (https://github.com/dmmorozo/GromacsTc).

Geometry optimization of the initial structure was performed in two steps. First, we carried out 1000 energy minimization steps at the AMBER03 force field level with L-BFGS algorithm. This MM optimization was followed by a second geometry optimization at the QM/MM level until the maximum force on any atom was below a threshold of 4 kJ/mol/nm. The QM region in these calculations consisted of the chromophore, three water molecules that are in direct hydrogen bonding contact with the chromophore, and the side chains of amino acids Arg66, Arg91, Glu144, Tyr177, His194, and Glu212. In addition, eight hydrogen link atoms were added on the chemical bonds connecting the QM and MM subsystems. The QM region thus contained 120 atoms, which are shown in ball-and-stick representation in Extended Data Fig. 8b. The QM region was modelled with Density Functional Theory (DFT), using the PBE0 functional[57] in combination with the cc-pVDZ basis set[58] and the DFT-D3 Grimme's empirical dispersion corrections[59]. To perform energy minimization in the electronic excited state, we used the Time-Dependent DFT within the Tamm-Dancoff approximation[60].

To locate the transition state between the A_1 and A_2 conformations on both ground (S_0) and excited (S_1) states an interpolation procedure was used. First, we interpolated the geometry from A_1 to A_2. Then, for each interpolation point, the geometry was optimized using constraints on the distances (i) between the chromophore phenol oxygen and the carboxylate of Glu144, (ii) between the chromophore phenol oxygen atoms and the water molecule, and (iii) between the carboxylate of Glu144 and the water molecules. The constrained distances are indicated by the dashed red lines in Extended Data Fig. 8b. Even if this setup does not guarantee that the lowest energy transitions state is found, the geometry with the lowest potential energy can be considered an upper bound to the actual transitions state.

An energy diagram based on the energies of the optimized (local) minima and transition states in both the S_0 and S_1 electronic states, is shown in Extended Data Fig. 8c. The results of the QM/MM optimizations suggest that there are indeed two energy minima, which we assign to the A_1 and A_2 conformations. In S_0 the A_1 conformation is more stable than the A_2 conformation by 8.97 kJ/mol. In S_1 the order reverses and the energy of the A_2 conformation is 7.98 kJ/mol below that of the A_1 conformation. The barriers connecting these minima are 38.11 and 21.16 kJ/mol in the ground and excited state, respectively. While the calculated barriers would be too high to account for ultrafast transitions between A_1 and A_2, we emphasize that the barriers are upper bound estimates.

To compare with spectroscopic data, we also computed the absorption and emission wavelengths at the XMCQDPT2[61] multiconfigurational correlated level of theory. These calculations were performed with the Firefly[62] package. The CASSCF reference wavefunction for the XMCQDPT2 calculations was based on an active space with 12 electrons distributed over 11 π-orbitals on the chromophore and was averaged over the five lowest singlet states. In these calculations, the rest of the protein was included as MM point charges that can polarize the QM wave function. The results of these XMCQDPT2/SA5-CASSCF(12,11)/cc-pVDZ//Amber03 QM/MM computations suggest a Stokes shift of 0.67 eV in the A_1 conformation and 0.77 eV in the A_2 conformation. These values are in line with the energy differences between the pump and probe pulses.

13. Density Matrix Simulations & Wigner Phase Space Analysis

To compliment the experimental findings non-perturbative density matrix simulations were performed. This simulation demonstrates a proof of principle, that vibrational coherence in an excited electronic state can be transferred to a lower energy electronic state via a dump interaction. A Wigner phase space analysis of the density matrix for the model system was used to elucidate the coherence and population dynamics of the system.

The density matrix formalization is useful for describing the statistical state at which a quantum mechanical system is in. The density matrix encodes information on the populated states through the diagonal elements of the matrix. The interaction of a system with an electric field on the coherent timescale will excite multiple states coherently and is experimentally visible in oscillations in the transient signal. These coherences between states are encoded in the off-diagonal elements of the density matrix. Off-diagonal elements between states in the same electronic level represent vibrational coherence whereas those that occur between electronic states characterize electronic coherence. As such the density matrix formalization allows for the observation of population and coherence.

A four-level system was used as a simple model of the rsKiiro fluorescent protein. Containing two vibrational states in both a ground and excited electronic state with the vibrational frequency set to 170 cm^{-1} for both levels. The excited state levels were set such that the 400 nm experimental pump could populate both vibrational levels of the excited state ($|2\rangle$ & $|3\rangle$) from, predominately, the vibrationally cold ground electronic state ($|0\rangle$) (Supplementary Fig. 53). The dump pulse carrier frequency was selected to maximize the dump population efficiency and is red shifted from the pump pulse.

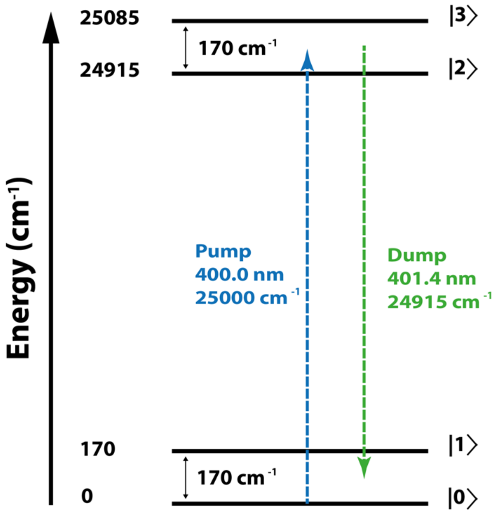

Supplementary Fig. 53 | Energy level diagram of four level system used to simulate coherence and population dynamics in rs-Kiiro protein.

The four-level (16 element) density matrix simulation evolves under the Liouville von Neumann equation, with a Hamiltonian that can be deconstructed into three separate contributions: An unperturbed term which describes the energy of the system, a perturbed term which contains the perturbation due to the laser(s) electric field(s) and a final relaxation term which describes the population dynamics of the system. A full derivation of the density matrix formulation is shown later. The time dependent density matrix elements evolve via the following rate equations where the off-diagonal elements ρ_{ij} are the coherence between states and diagonal elements ρ_{ii} are the population in that respective state, where

$\sum \rho_{ii} = 1$. $\omega_{ij} = \frac{|\varepsilon_i - \varepsilon_i|}{\hbar}$ are the transition frequencies between states i and j with energies $\varepsilon_{i,j}$. E is the time dependent electric field of the laser pulse(s) and μ_{ij} is the transitional dipole moment between states i and j. $\bar{\Gamma}_{ij}$ is the off-diagonal decay rate, defined as $\bar{\Gamma}_{ij} = \frac{(\gamma_{ii} + \gamma_{jj})}{2} + \Gamma_{ij}$, where γ_{ii} is the decay rate of state i and Γ_{ij} is the pure dephasing rate of the coherence between i and j. All rates are defined as the inverse of the decay/dephasing time e.g. $\Gamma_{ij} = T_{ij}^{-1}$, where T_{ij} is the dephasing time between states i and j.

Off-diagonal elements rate equations:

$$\dot{\rho}_{01} = -\bar{\Gamma}_{01}\rho_{01} + i\omega_{10}\rho_{01} + \frac{iE}{\hbar}(\mu_{02}\rho_{21} + \mu_{03}\rho_{31} - \mu_{12}\rho_{20} - \mu_{13}\rho_{30}) \quad \text{(S34)}$$

$$\dot{\rho}_{02} = -\bar{\Gamma}_{02}\rho_{02} + i\omega_{20}\rho_{02} + \frac{iE}{\hbar}(\mu_{02}(\rho_{22} - \rho_{00}) + \mu_{03}\rho_{32} - \mu_{12}\rho_{01}) \quad \text{(S35)}$$

$$\dot{\rho}_{03} = -\bar{\Gamma}_{03}\rho_{03} + i\omega_{30}\rho_{03} + \frac{iE}{\hbar}(\mu_{03}(\rho_{33} - \rho_{00}) + \mu_{02}\rho_{23} - \mu_{13}\rho_{01}) \quad \text{(S36)}$$

$$\dot{\rho}_{12} = -\bar{\Gamma}_{12}\rho_{12} + i\omega_{21}\rho_{12} + \frac{iE}{\hbar}(\mu_{12}(\rho_{22} - \rho_{11}) + \mu_{13}\rho_{32} - \mu_{02}\rho_{10}) \quad \text{(S37)}$$

$$\dot{\rho}_{13} = -\bar{\Gamma}_{13}\rho_{13} + i\omega_{31}\rho_{13} + \frac{iE}{\hbar}(\mu_{13}(\rho_{33} - \rho_{11}) + \mu_{12}\rho_{23} - \mu_{03}\rho_{10}) \quad \text{(S38)}$$

$$\dot{\rho}_{23} = -\bar{\Gamma}_{23}\rho_{23} + i\omega_{32}\rho_{23} + \frac{iE}{\hbar}(\mu_{02}\rho_{03} + \mu_{12}\rho_{13} - \mu_{03}\rho_{20} - \mu_{13}\rho_{21}) \quad \text{(S39)}$$

$$\bar{\Gamma}_{ij} = \frac{(\gamma_{ii} + \gamma_{jj})}{2} + \Gamma_{ij} \quad \text{(S40)}$$

Diagonal elements rate equations:

$$\dot{\rho}_{11} = -\gamma_{11}\rho_{11} + \gamma_{12}\rho_{22} + \gamma_{13}\rho_{33} + \frac{iE}{\hbar}\big(\mu_{12}(\rho_{21} + \rho_{12}) + \mu_{13}(\rho_{31} - \rho_{13})\big) \quad \text{(S41)}$$

$$\dot{\rho}_{22} = -\gamma_{22}\rho_{22} + \gamma_{23}\rho_{33} + \frac{iE}{\hbar}\big(\mu_{02}(\rho_{02} + \rho_{20}) + \mu_{12}(\rho_{12} - \rho_{21})\big) \quad \text{(S42)}$$

$$\dot{\rho}_{33} = -\gamma_{33}\rho_{33} + \frac{iE}{\hbar}\big(\mu_{03}(\rho_{03} - \rho_{30}) + \mu_{13}(\rho_{13} - \rho_{31})\big) \quad \text{(S43)}$$

$$\dot{\rho}_{00} = -\dot{\rho}_{11} - \dot{\rho}_{22} - \dot{\rho}_{33} \quad \text{(S44)}$$

The electric field was simulated as transform limited Gaussian pulse $\tilde{E}_0(\omega)$ which is spectrally shaped via a spectral phase transfer function $\varphi(\omega)$.

$$\tilde{E}(\omega) = \tilde{E}_0(\omega)\exp\big(i\varphi(\omega)\big) \quad \text{(S45)}$$

The spectral phase transfer function is a Taylor polynomial where the first order constant *b* determines the temporal position of the pulse and the second order constant *c* is the spectral chirp of the pulse.

$$\varphi(\omega) = a(\omega_0) + b\big(\omega_0(\omega - \omega_0)\big) + \frac{c}{2}(\omega_0(\omega - \omega_0)^2) + \frac{d}{6}(\omega_0(\omega - \omega_0)^3) \ldots \quad \text{(S46)}$$

The simulated time-dependent density matrix was then analyzed by a Wigner transform, to give the probability distribution in phase space. The Wigner function yields a classical like distribution, quantifying the probability of finding the system with momentum *q* and position *p*. This classical representation of a quantum mechanical system is an approximation, as Heisenberg's uncertainty

principle forbids simultaneous measurement of momentum and position. Such the function is a quasiprobability distribution and can be negative.

Computing the Wigner function allows a topographical representation to be constructed. These 3-dimensional representations of position, momentum and the Wigner quasi-probability distributions allow a visual representation of the population and coherence dynamics of the system constructed from the density matrix. The plots materialize as a superposition of individual Fock states, if coherence (off-diagonal) elements are absent the plots are spherically symmetric. In this case the Wigner distribution of that system, is a linear combination of the individual Fock states. The coherence between states is observed as spherical asymmetries in the plots, where the number of lines of symmetry is equal to the number difference between the Fock states which that coherence is observed. Such a coherence between state $|3\rangle$ and $|1\rangle$ the Wigner distribution would have two (3-1) lines of symmetry. The coherence amplitude is proportional to the relative spherical difference in the plot. These properties of the Wigner function allow the separation of plots for different electronic states if electronic coherence is ignored. A full derivation of the computational Wigner function follows.

13.1. Density Matrix Simulations

The following is a full derivation of the formulization of the non-perturbative, time-dependent density simulations. This code was written by the Buckup Group in Heidelberg University[63,64].

Schrödinger Equation for evolution of wavefunction $|\psi(t)\rangle$:

$$i\hbar\frac{d}{dt}|\psi(t)\rangle = H(t)|\psi(t)\rangle \tag{S47}$$

Deriving the Louisville-Von Neumann Equation by product rule:

$$\frac{d}{dt}\underbrace{\rho(t)}_{|\psi(t)\rangle\langle\psi(t)|} = \underbrace{\left(\frac{d}{dt}|\psi(t)\rangle\right)}_{\frac{-i}{\hbar}H|\psi(t)\rangle}\langle\psi(t)| + |\psi(t)\rangle\underbrace{\left(\frac{d}{dt}\langle\psi(t)|\right)}_{\frac{i}{\hbar}\langle\psi(t)|H}$$

$$\frac{d}{dt}\rho(t) = \frac{-i}{\hbar}H\underbrace{|\psi(t)\rangle\langle\psi(t)|}_{\rho(t)} + \frac{i}{\hbar}\underbrace{|\psi(t)\rangle\langle\psi(t)|}_{\rho(t)}H = \frac{-i}{\hbar}[H,\rho(t)]$$

$$i\hbar\frac{d}{dt}\rho(t) = [H(t),\rho(t)] \tag{S48}$$

The total Hamiltonian is expressed in the three terms: an unperturbed term, the perturbed term which contains the perturbation due to the laser and a final term which describes the relaxation of the system:

$$i\hbar\frac{d}{dt}\rho(t) = [H_0(t),\rho(t)] + [H_I(t),\rho(t)] + [H_R(t),\rho(t)] \tag{S49}$$

The unperturbed term contains the energy of the system without any interaction and is a diagonal matrix:

$$H_0(t) = \hbar\begin{bmatrix}\omega_0 & 0 & \cdots & 0\\ 0 & \omega_1 & \cdots & 0\\ \vdots & \vdots & \ddots & \vdots\\ 0 & 0 & \cdots & \omega_n\end{bmatrix} \tag{S50}$$

$$[H_0(t),\rho(t)] = -\hbar\begin{bmatrix}0 & \omega_{0,1}\cdot\rho_{0,1}(t) & \cdots & \omega_{0,n}\cdot\rho_{0,n}(t)\\ \omega_{1,0}\cdot\rho_{1,0}(t) & 0 & \cdots & \omega_{1,n}\cdot\rho_{1,n}(t)\\ \vdots & \vdots & \ddots & \vdots\\ \omega_{n,0}\cdot\rho_{n,0}(t) & \omega_{n,1}\cdot\rho_{n,1}(t) & \cdots & 0\end{bmatrix} \tag{S51}$$

The interaction term describes the coupling of the laser field with electronic coherence via the transition dipole moments:

$$H_I(t) = \begin{bmatrix} 0 & \vec{\mu}_{0,1} \cdot \vec{E}(t) & \cdots & \vec{\mu}_{0,n} \cdot \vec{E}(t) \\ \vec{\mu}_{1,0} \cdot \vec{E}(t) & 0 & \cdots & \vec{\mu}_{1,n} \cdot \vec{E}(t) \\ \vdots & \vdots & \ddots & \vdots \\ \vec{\mu}_{n,0} \cdot \vec{E}(t) & \vec{\mu}_{n,1} \cdot \vec{E}(t) & \cdots & 0 \end{bmatrix} \qquad \text{(S52)}$$

Noting $\vec{\mu}_{i,j} = \vec{\mu}_{j,i}$

$$[H_I(t), \rho(t)]$$
$$= -\vec{E}(t) \begin{bmatrix} \sum_{i \neq 0}^{n} \vec{\mu}_{0,i}(\rho_{0,i}(t) - \rho_{i,0}(t)) & \vec{\mu}_{0,1}(\rho_{0,0}(t) - \rho_{1,1}(t)) + \sum_{i \neq 0,1}^{n} (\vec{\mu}_{i,1} \cdot \rho_{0,i}(t) - \vec{\mu}_{0,i} \cdot \rho_{i,1}(t)) & \cdots & \vec{\mu}_{0,n}(\rho_{0,0}(t) - \rho_{n,n}(t)) + \sum_{i \neq 0,n}^{n} (\vec{\mu}_{i,n} \cdot \rho_{0,n}(t) - \vec{\mu}_{0,n} \cdot \rho_{i,n}(t)) \\ \vec{\mu}_{1,0}(\rho_{1,1}(t) - \rho_{0,0}(t)) + \sum_{i \neq 1,0}^{n} (\vec{\mu}_{i,0} \cdot \rho_{1,i}(t) - \vec{\mu}_{1,i} \cdot \rho_{i,0}(t)) & \sum_{i \neq 1}^{n} \vec{\mu}_{1,i}(\rho_{1,i}(t) - \rho_{i,1}(t)) & \cdots & \vec{\mu}_{1,n}(\rho_{1,1}(t) - \rho_{n,n}(t)) + \sum_{i \neq 1,n}^{n} (\vec{\mu}_{i,n} \cdot \rho_{1,i}(t) - \vec{\mu}_{1,i} \cdot \rho_{i,n}(t)) \\ \vdots & \vdots & \ddots & \vdots \\ \vec{\mu}_{n,0}(\rho_{n,n}(t) - \rho_{0,0}(t)) + \sum_{i \neq n,0}^{n} (\vec{\mu}_{i,0} \cdot \rho_{n,i}(t) - \vec{\mu}_{n,i} \cdot \rho_{i,0}(t)) & \vec{\mu}_{n,1}(\rho_{n,n}(t) - \rho_{1,1}(t)) + \sum_{i \neq n,1}^{n} (\vec{\mu}_{i,1} \cdot \rho_{n,i}(t) - \vec{\mu}_{n,i} \cdot \rho_{i,1}(t)) & \cdots & \sum_{i \neq n}^{n} \vec{\mu}_{n,i}(\rho_{n,i}(t) - \rho_{i,n}(t)) \end{bmatrix}$$

The relaxation term is described phenomenologically as populations decay times ($1/T_{ii}$) for the diagonal terms and coherence times ($1/T_{ij}$) for the off-diagonal terms:

$$[H_R(t), \rho(t)] = i\hbar \begin{bmatrix} 1/T_{0,0} \cdot \rho_{0,0}(t) & -1/T_{0,1} \cdot \rho_{0,1}(t) & \cdots & -1/T_{0,n} \cdot \rho_{0,n}(t) \\ -1/T_{1,0} \cdot \rho_{1,0}(t) & -1/T_{1,1} \cdot \rho_{1,1}(t) & \cdots & -1/T_{1,n} \cdot \rho_{1,n}(t) \\ \vdots & \vdots & \ddots & \vdots \\ -1/T_{n,0} \cdot \rho_{n,0}(t) & -1/T_{n,1} \cdot \rho_{n,1}(t) & \cdots & -1/T_{n,n} \cdot \rho_{n,n}(t) \end{bmatrix} \qquad \text{(S53)}$$

13.2. Wigner Phase Space Analysis

The following is a full derivation of how a numerical algorithm that can calculate the Wigner quasi-probability distribution of a system from the time dependent density matrix. The Laguerre polynomial method for calculating the Wigner function was used, courtesy of the open source Quantum Toolbox in Python (QUTIP) package[65]. Derivation is adapted from "Measuring the Quantum State of Light" Ulf Leonhardt[66]. Expressing the density matrix in the Fock basis, where M is the number of states (energy levels) in the system:

$$\hat{\rho} = \sum_{m,n=0}^{M} \rho_{mn} |m\rangle\langle n| \qquad \text{(S54)}$$

Inserting into the Fock basis expansion of the density matrix into Wigner's formula yields:

$$W(q,p) = \sum_{m,n=0}^{M} \rho_{mn} W_{mn}(q,p) \qquad \text{(S55)}$$

With:

$$W_{mn}(q,p) \quad = \frac{1}{\pi} \int_{-\infty}^{+\infty} \exp\,(2ipx) \langle q - x \mid m \rangle \langle n \mid q + x \rangle dx \qquad \text{(S56)}$$

Schrodinger's wave functions are off for odd states and even for even states such we obtain an expression for $W_{mn}(q,p)$:

$$W_{mn}(q,p) = \frac{(-1)^m}{\pi}\int_{-\infty}^{+\infty} \exp(2ipx)\langle x-q \mid m\rangle\langle n \mid x+q\rangle dx \tag{S57}$$

This function for $W_{mn}(q,p)$ is equivalent to the Fourier-transformed distribution, $\tilde{W}_{mn}$, known as the characteristic function:

$$W_{mn}(q,p) = \frac{(-1)^m}{\pi}\tilde{W}_{mn}(-2p,2q) \tag{S58}$$

The characteristic function can be expressed in polar coordinates $\exp(-\,\mathrm{i}p\hat{q} - \mathrm{i}q\hat{p})$:

$$\begin{aligned} W_{mn}(q,p) &= \frac{(-1)^m}{\pi}\mathrm{tr}\,\{|m\rangle\langle n|\exp(2\mathrm{i}p\hat{q} - 2\mathrm{i}q\hat{p})\} \\ &= \frac{(-1)^m}{\pi}\langle n|\exp(2\mathrm{i}p\hat{q} - 2\mathrm{i}q\hat{p})|m\rangle \\ &= \frac{(-1)^m}{\pi}\langle n|\hat{D}(2\alpha)|m\rangle \end{aligned} \tag{S59}$$

Where the complex amplitude (α) can be decomposed into a real an imaginary component $\alpha = 2^{-1/2}(q + \mathrm{i}p)$ and $\hat{D}$ is the Hermitian displacement operator. Expressing the displacement operator in the form of the Baker-Hausdorff formula and expanding using the following property for the creation and annihilation operator of Fock States:

$$\hat{a}^{v}|n\rangle = \left[\frac{n!}{(n-v)!}\right]|n-v\rangle \tag{S60}$$

$$\begin{aligned} \langle n|\hat{D}(2\alpha)|m\rangle = \;& \exp(-2|\alpha|^2)\sum_{v=0}^{n}\sum_{\mu=0}^{m}(2\alpha)^{v}(-2\alpha^*)^{\mu} \\ & \times\frac{1}{v!\,\mu!}\left[\frac{n!\,m!}{(n-v)!\,(m-\mu)!}\right]^{1/2} \\ & \times\langle n-v \mid m-\mu\rangle \end{aligned} \tag{S61}$$

Using the orthonormal properties of Fock states, the double sum is reduced to a single sum with a polynomial of $|\alpha|^2$. Employing Laguerre polynomials L_m^k , the displacement operator reduces to:

For $m \geq n$

$$\langle n|\hat{D}(2\alpha)|m\rangle = \left(\frac{n!}{m!}\right)^{1/2}\exp(-2|\alpha|^2)(-2\alpha^*)^{m-n}L_n^{m-n}(4|\alpha|^2) \tag{S62}$$

And for $m < n$

$$\langle n|\hat{D}(2\alpha)|m\rangle = \langle m|\hat{D}(-2\alpha)|n\rangle^* \tag{S63}$$

Recasting q and p in phase space polar coordinates r and φ:

$$q = r\cos\varphi, p = r\sin\varphi$$

Such $\alpha = 2^{-1/2}\,r\exp(-\mathrm{i}\varphi)$ and expanding Equation (S55), where $k = m - n$:

$$W(q,p) = \sum_{k=-M}^{M} w(r,k)\exp(-\mathrm{i}k\varphi) \tag{S64}$$

With

$$w(r,k) = \begin{cases} \sum_{n=0}^{M-k} w_n(r,k)\rho_{n+k,n} & \text{for } k \geq 0 \\ w(r,-k)^* & \text{for } k < 0 \end{cases} \tag{S65}$$

And

$$w_n(r,k) = \frac{1}{\pi}(-1)^n \left[\frac{n!}{(n+k)!}\right]^{1/2} \exp\left(-r^2\right)(r\sqrt{2})^k L_n^k(2r^2) \tag{S66}$$

The above can be calculated numerically as the recurrence relation of Laguerre polynomials allows $w_n(r,k)$ to be expressed as:

$$\begin{aligned} w_n(r,k) = \; & \frac{1}{\sqrt{n(n+k)}}\left[(2r^2+1-k-2n)w_{n-1}(r,k)\right. \\ & \left. -\sqrt{(n-1)(n-1+k)}w_{n-2}(r,k)\right] \end{aligned} \tag{S67}$$

With the initial value of recurrence:

$$w_0(r,k) = \frac{1}{\pi}(k!)^{-1/2}(r\sqrt{2})^k \exp\left(-r^2\right) \tag{S68}$$

$$w_0(0,0) = \frac{1}{\pi} \tag{S69}$$

and

$$w_{-1}(r,k) = 0 \tag{S70}$$

Such the $w_n(r,k)$ function in summation of Equation (S65) is efficiently calculated *en passant*. Defining a phase space projection over which the algorithm can compute the Wigner function allow a topographical representation to be constructed. These 3-dimensional representations of position, momentum and the Wigner quasi-probability distributions allows a visual representation of the population and coherence dynamics of the system constructed from the density matrix.

13.3. Simulation Parameters

Simulation parameters chosen to best replicate the experimental values, such that the population and moreover the coherence dynamics were representative of rsKiiro's are shown in Supplementary Table. 11. The electronic and vibrational coherence was presumed to have a dephasing time of 100 fs (Supplementary Table. 1) and 1 ps[67]. The excited state lifetime was set to the experimentally measured 50 ps and a vibrational relaxation time of 1 ps for both ground and excited states[67]. The initial state populations were thermally populated with a Boltzmann distribution at 298 K. The full width half maximum of the Gaussian laser electric fields matched the experimentally used 100 fs for both the pump and dump pulses. The peak amplitude of the laser pulse was arbitrarily set at 5 x 10^7 V m^{-1} and the transition dipole moments to 4.2 x 10^{-29} C m for a maximum excitation efficiency in pumping.

Supplementary Table. 11 | Density matrix simulation parameters.

Parameter		Value
Dephasing of coherence	Electronic $(\Gamma_{el})^{-1}$	100 fs
	Vibrational $(\Gamma_{el})^{-1}$	1 ps
Decay rates	$\|0,1\rangle \rightarrow \|0,0\rangle \quad (\gamma_{01})^{-1}$	1 ps
	$\|1,0\rangle \rightarrow \|0,1\rangle \quad (\gamma_{21})^{-1}$	20 ps
	$\|1,1\rangle \rightarrow \|1,0\rangle \quad (\gamma_{32})^{-1}$	1 ps
Starting populations in states at 298 k, thermally populated	$\|0\rangle$	0.69
	$\|1\rangle$	0.31
	$\|2\rangle$	4.0×10^{-53}
	$\|3\rangle$	1.7×10^{-53}
Transition dipole moment	μ	4.20×10^{-29} C m
Peak amplitude of laser electric field	Pump	5×10^{7} V m^{-1}
	Dump	5×10^{7} V m^{-1}
Duration of Gaussian laser pulse		
	Pump	100 fs
	Dump	100 fs
Dump delay time		350 fs

Transition dipole moment coupling coefficient matrix, for dipole allowed transitions:

$$\begin{array}{c} |0\rangle \\ |1\rangle \\ |2\rangle \\ |3\rangle \end{array} \begin{bmatrix} 0 & 0 & \mu & \mu \\ 0 & 0 & \mu & \mu \\ \mu & \mu & 0 & 0 \\ \mu & \mu & 0 & 0 \end{bmatrix}$$
$$\quad |0\rangle \quad |1\rangle \quad |2\rangle \quad |3\rangle$$

Coherence dephasing matrix:

$$\begin{array}{c} |0\rangle \\ |1\rangle \\ |2\rangle \\ |3\rangle \end{array} \begin{bmatrix} 0 & \Gamma_{vib} & \Gamma_{el} & \Gamma_{el} \\ \Gamma_{vib} & 0 & \Gamma_{el} & \Gamma_{el} \\ \Gamma_{el} & \Gamma_{el} & 0 & \Gamma_{vib} \\ \Gamma_{el} & \Gamma_{el} & \Gamma_{vib} & 0 \end{bmatrix}$$
$$\quad |0\rangle \quad |1\rangle \quad |2\rangle \quad |3\rangle$$

Decay rates between states:

$$\begin{array}{c} |0\rangle \\ |1\rangle \\ |2\rangle \\ |3\rangle \end{array} \begin{bmatrix} 0 & \gamma_{01} & 0 & 0 \\ 0 & \gamma_{11} & \gamma_{21} & 0 \\ 0 & 0 & \gamma_{22} & \gamma_{32} \\ 0 & 0 & 0 & \gamma_{33} \end{bmatrix}$$
$$\quad |0\rangle \quad |1\rangle \quad |2\rangle \quad |3\rangle$$

Where $\gamma_{01} = \gamma_{11}, \gamma_{21} = \gamma_{22}$ and $\gamma_{32} = \gamma_{33}$

13.4. Pump vs Pump-Dump scheme

Time evolution of diagonal elements of the density matrix are plotted in Supplementary Fig. 54. Time evolution of diagonal elements of the density matrix are plotted in Supplementary Fig. 55. Initially both $|0\rangle$ and $|1\rangle$ are thermally populated, some small population relaxation to the vibrational cold state occurs pre pump pulse due to rate equations. Upon excitation with pump laser pulse both electronic excited states $|2\rangle$ and $|3\rangle$ are populated. In the pump only scheme a gradual decay of 1 ps for both electronic states back to the vibrationally cold states is observed. The long-lived excited state (~20 ps) shows no substantial decay back to ground state over the duration of the simulation. The dump pulse predominately transfers from $|3\rangle \rightarrow |1\rangle$ and $|2\rangle \rightarrow |1\rangle$, in line with schematic level diagram of the simulation Supplementary Fig. 53. Summation of the diagonal elements of states in the same electronic level are shown in Supplementary Fig. 55. The dump efficiency differs from the experimentally observed complete dumping, due to the limited vibrational levels used in the simulation.

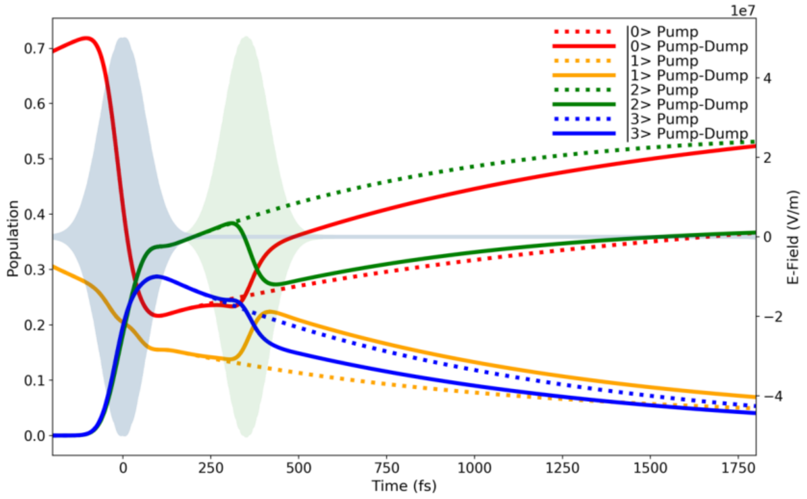

Supplementary Fig. 54 | Time evolution of diagonal elements of the density matrix calculation. Diagonal elements of the four states show the population dynamics in the pump (dotted) and pump-dump (solid) scheme. The pale blue and green Gaussians show the real part of the laser electric field with a pump-dump delay of 350 fs. Population is initially in a thermal distribution in states |0> and |1> before being excited to both the |2> and |3> states by the pump pulse. The dump pulse facilitates a population transfer back down to ground electronic state.

The vibrational coherence between states in the same electronic level is shown in Supplementary Fig. 56. The modulus of the complex off-diagonal elements is depicted for both ground state in yellow ($|0,1\rangle$) and excited state in red. Coherence is initially generated in both ground and excited states by the pump pulse. The creation of ground state impulsive coherence in the (near-)resonant case is in the direction of the nuclear binding force. The excited state coherence is displacement driven.

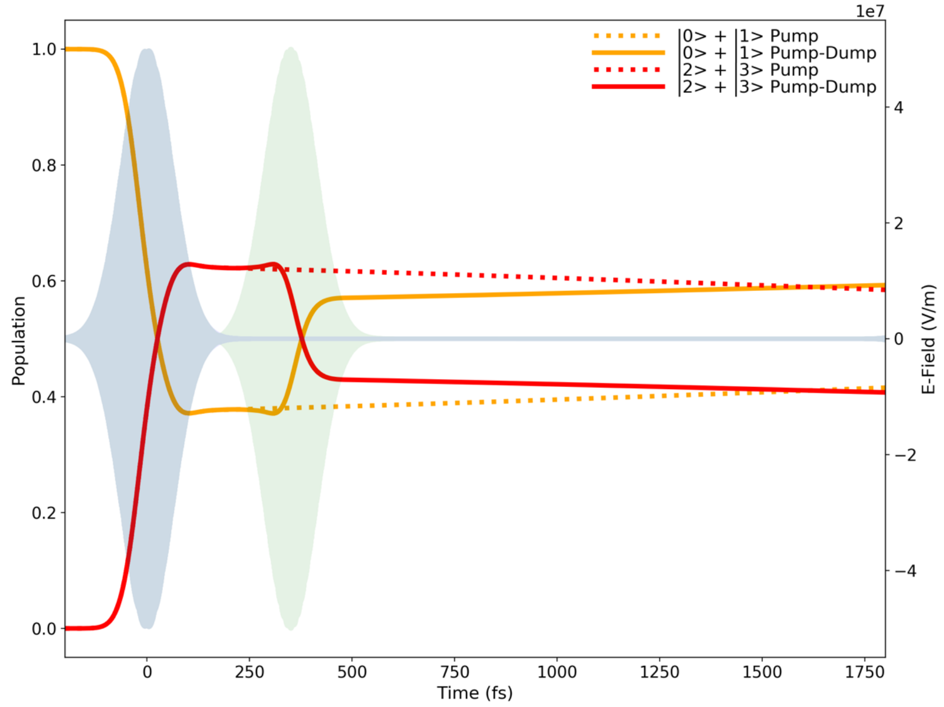

Supplementary Fig. 55 | Summation of the diagonal elements of states in the same electronic level. Ground state (yellow) |0> + |1> and excited state (red) |2> + |3>, shown for both pump (dotted) and pump-dump (solid) schemes. The pale blue and green Gaussians show the real part of the laser electric field with a pump-dump delay of 350 fs.

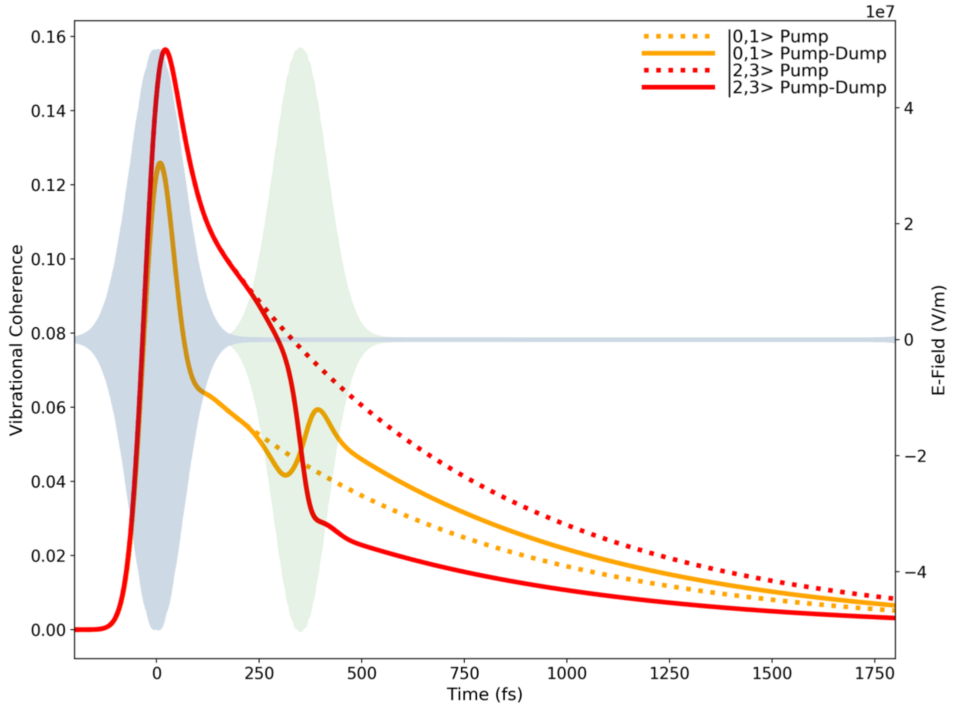

Supplementary Fig. 56 | Modulus of the off-diagonal elements show coherence between states in the same electronic level. Ground state vibrational coherence (|0,1>) shown in yellow and excited state vibrational coherence (|2,3>) shown in red for both pump (dotted) and pump-dump schemes (solid). The pale blue and green Gaussians show the real part of the laser electric field with a dump delay of 350 fs.

The dump pulse within the vibrational dephasing time facilitates a transfer of the coherent momentum in the excited state to the ground state. A sharp decline in the excited state coherence is observed within the timescale of the dump pulse and an increase in ground state coherence, compared with the no dump interaction. The remaining coherence in the excited state is due to the sub-optimal population transfer. It is noted that shorter pulses, imparting more vibrational coherence momentum in the pump case and produced a more effective coherence transfer.

A transfer of vibrational coherence from the excited electronic state to the ground electronic state is observed facilitated by the dump laser pulse.

The Wigner transforms for both experimental laser schemes are shown in Supplementary Fig. 57. The electronic coherence for ground and excited state Wigner plots have been neglected. The system can be deconstructed into components from the excited and ground states, these Wigner plots are shown. The full system can be seen to be a linear combination of the two electronic states minus the electronic coherent interactions, which have been ignored in the individual state plots. The effect of the electronic coherence can be observed in the full system plots centred on the two laser pulses. Fine structure can be observed in these plots as a superposition of many asymmetric coherence nodes, this fine structure quickly dissipates over the electronic dephasing time ($\sim$ 100 fs).

Population of the states can be observed by the amplitude of the Wigner plots. The dump population transfer is distinguished by the increase in the ground state amplitude and a decrease in the excited state, compared to the pump scheme.

The coherence in the states is displayed in the spherical asymmetry of the plots. The rotation of these nodes matches that of the vibrational frequency. In the pump scheme we see an initial large asymmetric node generated in both the ground and excited state. At longer times we see a gradual decay of the asymmetry until a spherically symmetric representation is formed, characteristic of a coherently cold state. The rate of the asymmetric node dissipating to a symmetric plot is characteristic of the vibrational dephasing rate.

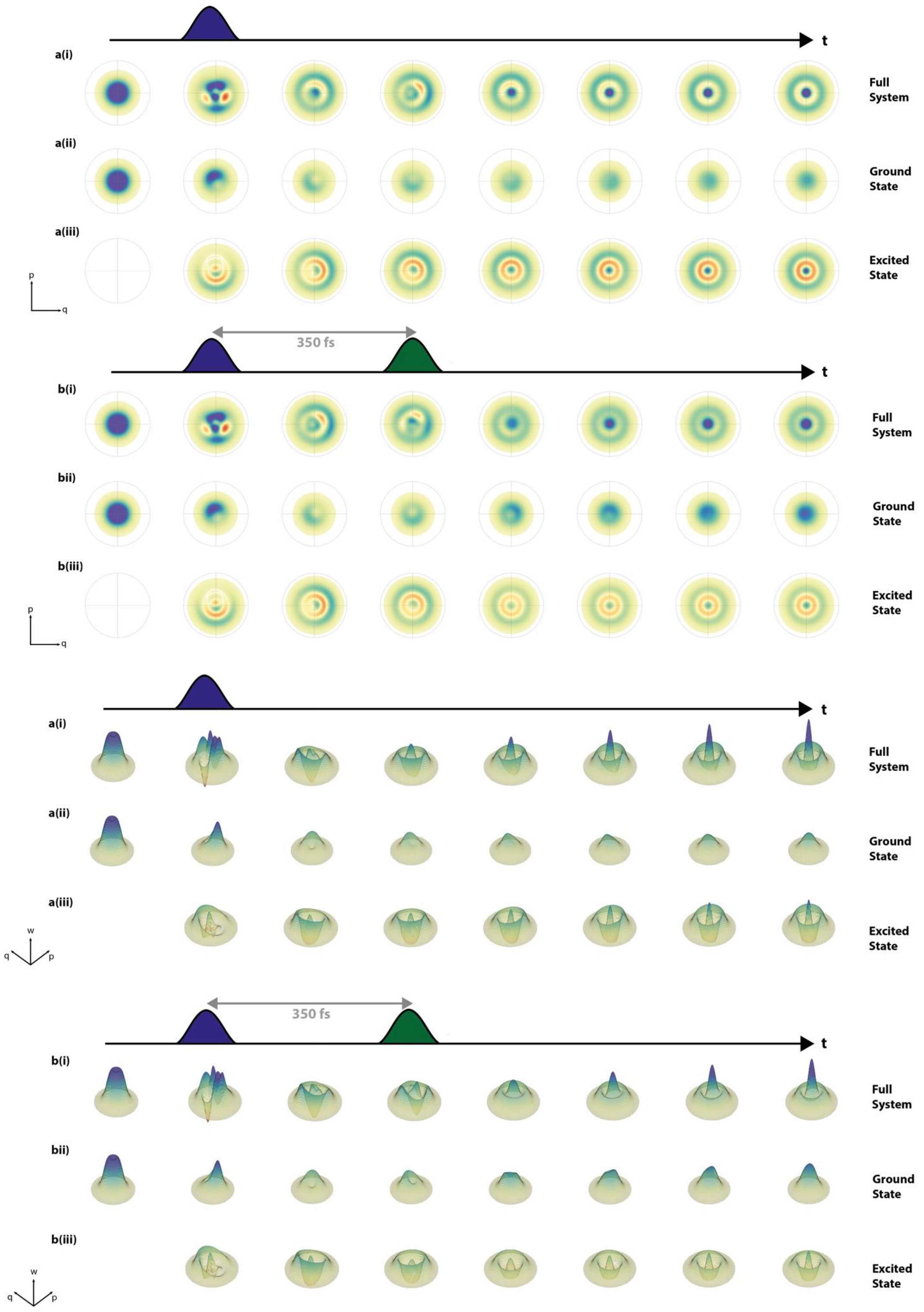

Supplementary Fig. 57 | Pump-probe and pump-dump-probe Wigner plots. Contour (top) and surface (bottom) plots of Wigner functions of the full system (i), ground electronic states (ii) and excited electronic states (iii), for both pump (a) and pump-dump (b) schemes at various time delays. The separated ground and excited state plots neglect electronic coherence, such structure can be observed beyond that of a superposition of the GS and ES plots. The electronic coherence decays with a time of 100 fs such beyond this after the pulse the remaining coherence in purely vibrational. The Wigner plots are shown at 175 fs intervals. Absolute scale has been used throughout all plots for the colour maps.

The coherent transfer of the dump is observed as a reduction in the asymmetry of the excited state Wigner plot and increase in asymmetry of the ground state. Such the ground state has a more pronounced node in the dump scheme and the opposite is true for the excited state.

The coherent dynamics around the dump pulse are examined in Supplementary Fig. 58. The ground state coherence transfer is observed after the peak of the laser pulse. In the first half the laser pulse a decrease in coherence is observed in the 300 fs Wigner plot. This coincides with a slight increase in excited state population observed in 300 fs, see Supplementary Fig. 55. Beyond this time, a large increase in coherence is observed over the cycle of the laser pulse. The prevalence is shown in the enhancement of the asymmetry observed between a(i) and a(ii) after the centre of the dump pulse.

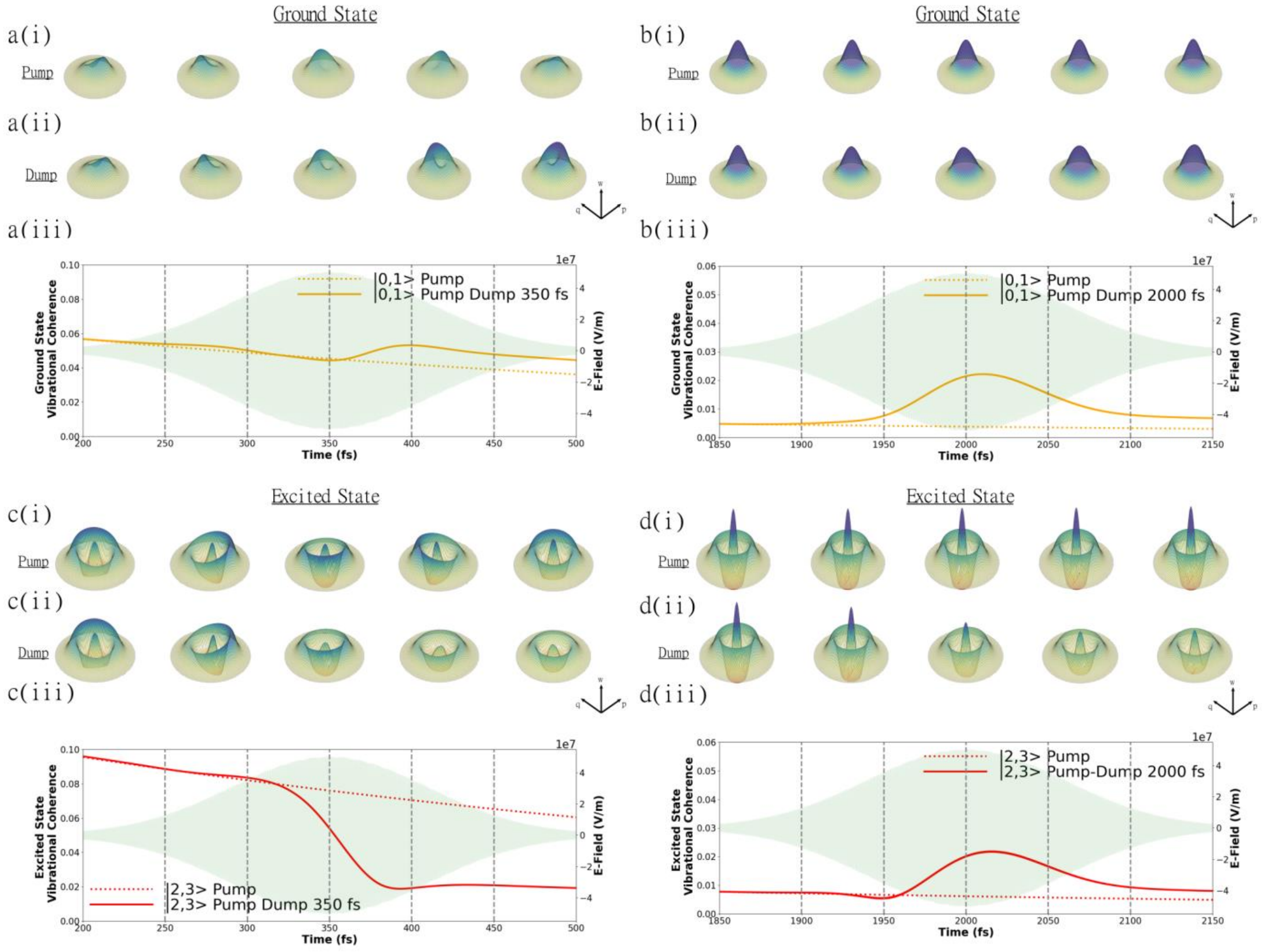

Supplementary Fig. 58 | Summary figure for density matrix calculations and Wigner transformations for ground and excited states. Results for a representative four-level system density matrix & Wigner transforms calculations on rsKiiro. Ground (a & b) and excited state (c & d) coherences for 350 fs (a & c) and 2 ps (b & d) pump-dump delay. In each case surface plots of Wigner functions are shown for the PP (i) and PDP (ii) cases, absolute scale is used for each colour map. Plots show the vibrational coherence (iii) (off-diagonal elements) of the ground electronic state, |0,1> (orange) and excited electronic state, |2,3> (red) in the pump (dashed) and pump-dump (solid) centred around the dump pulse.

Contrastingly the excited state coherence decreases from the beginning of the dump pulse. An almost 4-fold decrease in coherence in the excited state is observed. The large asymmetry in excited state Wigner plots at the start of the dump pulse is virtually symmetric by the end of the laser cycle. These simulations demonstrate a proof of principle that excited state vibrational coherence can be transferred to a lower electronic state by the simulated emission of a dump pulse.

13.5. Pump-Dump delay comparison

To compliment the SACLA experiment the effect of pump-dump delay time on the population and coherence dynamics of the toy system was investigated. A 350 fs and a 2 ps delay time was selected, replicating the TR-SFX experiment, the first being within the toy systems vibrational dephasing time (1 ps) and both delay times within the excited state lifetime (20 ps). The excited population dynamics for both dump delay schemes are shown in Supplementary Fig. 59. The dump efficiency decreases further in the 2 ps scheme (dashed) due to the pulse repopulating the largely vacant |3> state as vibrational cooling has occurred beyond 1 ps.

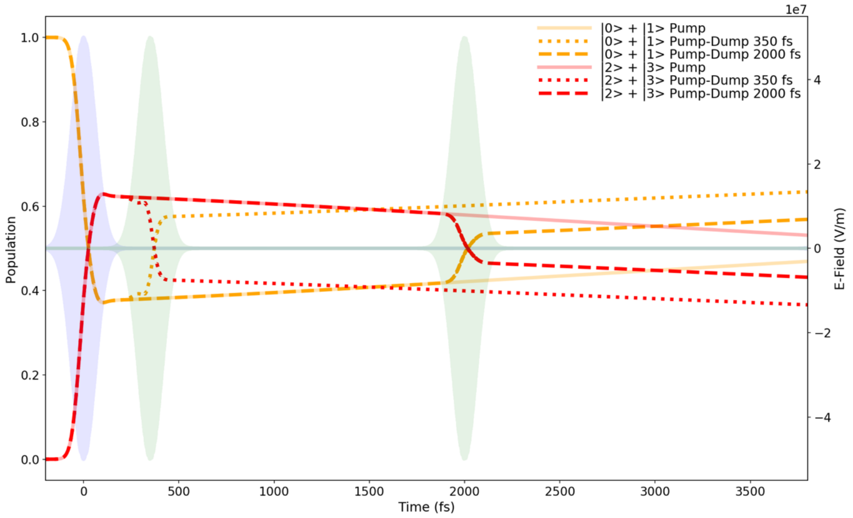

Supplementary Fig. 59| Summation of the diagonal elements of states in the same electronic level. Ground state (yellow) |0> + |1> and excited state (red) |2> + |3>, shown for both pump (solid), pump-dump 350 fs delay (dotted) and pump-dump 2 ps delay (dashed) schemes. The pale blue (pump) and green (dump) Gaussians show the real part of the laser electric field with either a dump delay of 350 fs or 2 ps.

The vibrational coherence of both pump-dump schemes is compared in Supplementary Fig. 60. As aforementioned in the Tannor-Rice optical control scheme, impulse momentum is imparted with the simulated emission dump pulse within the vibrational dephasing time of the system. Such in the 350 fs pump-dump delay, a transfer of vibrational coherence from the S_1 (|2,3>) to the S_0 (|0,1>) is observed (dotted line). Conversely the 2 ps delay creates vibrational coherence in both states (dashed line). Creation of vibrational coherence in the excited state is due to impulsive coherence in the direction of the nuclear binding force. Ground state coherence is displacement driven, the reversal of the pump coherence generated by the pump. The lack of a coherence transfer from excited to ground state in the 2 ps scheme due to the quenching of wave packet momentum beyond the 1 ps dephasing time validating the Tannor-Rice coherent dynamics.

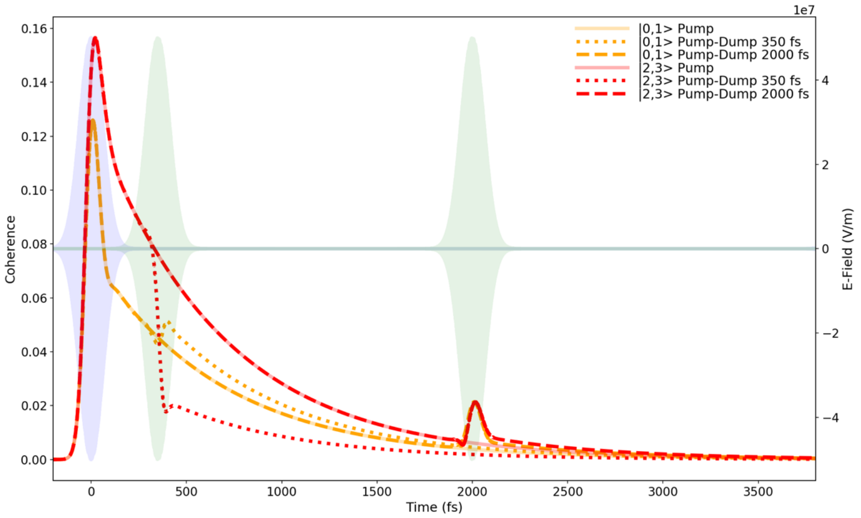

Supplementary Fig. 60| Modulus of the off-diagonal elements show coherence between states in the same electronic level. Ground state vibrational coherence (|0,1>) shown in yellow and excited state vibrational coherence (|2,3>) shown in red for both pump (solid), pump-dump 350 fs delay (dotted) and pump-dump 2 ps delay (dashed) schemes. The pale blue (pump) and green (dump) Gaussians show the real part of the laser electric field with either a dump delay of 350 fs or 2 ps.

Further elucidation of the dynamics can be garnered from a comparison of the Wigner distributions of the system over the laser cycle in both pump-dump schemes. Supplementary Fig. 61 shows the ground electronic state dynamics with the Wigner distributions plotted at 25 fs intervals over the central 100 fs of the dump pulse. In the early time of the 350 fs dump pulse, a continuing rotation of an asymmetric node (shown in Supplementary Fig. 61a(ii)) matches the vibrational frequency (170 cm^{-1}). The rotation of node is halted over the central portion of the laser pulse and growth of population is visible in both the positional and momentum axis. This is facilitated by both impulsive momentum and positional displacement being transferred from S_1 to the S_0 by stimulated emission, analogous to a Tannor-Rice scheme. In the 2 ps scheme the ground state distribution has no asymmetric nodes and is centralized. Upon interaction with central portion of the dump pulse the distribution is perturbed in both the position (q) and momentum (p) dimension. The positional perturbation of the maximum of the distribution is comparable in both dump schemes, however a far larger perturbation in the momentum axis is observed in the 350 fs scheme within the vibrational dephasing of the system.

The excited state Wigner distributions are shown in Supplementary Fig. 62, the 350 fs pump-dump scheme exhibits a loss of vibrational coherence in the excited state whereas a generation of coherence is observed in the 2 ps. The loss of coherence in the S_1 in the shorter delay attributed to removal of population in the coherently excited state. The generation in the longer scheme attributed to a combination of detuning of the dump pulse, causing a small positional displacement in lower vibrational state and repopulation of the higher vibrational level which population has relaxed out of at longer times. Reference Wigner plots in both surface and contour are shown in Supplementary Fig. 63 for reference.

13.6. Wavepacket assignment and discussion of coherence parameters and simulation

We discuss the wavepacket assignment in the framework of the existing Raman spectroscopy literature[68–71] and furthermore provide a coherence simulation with parameters relevant to the experimental conditions and observations. Pulsed resonant excitation of the molecular system prepares vibrational coherences in both the ground and excited states. The magnitude, phase and frequency limits

are determined by the electronic and vibrational dephasing, the detuning, pulse duration, intensity, carrier frequency and spectral phase. It is important to note that for a laser spectrum that is narrower than the absorption spectrum, the coherence magnitude of the ground state wavepacket is population driven and further enhanced by moderate detuning as are conditions typically used for TR-SFX and a representative of our PP data. The imparted impulse momentum in the ground state is in the direction of the nuclear binding force, while the excited state wavepacket is purely displacement driven and receives initial position and phase. For the ground state with small detuning the displacement is minimal in the lowest well that corresponds to the dark structure with the impulse momentum as shown in Fig. 2. Having shown the picoseconds time scale transfer of the PDP product back to the dark structure, coherence transfer between wells is described as adiabatic transfer on a double well potential energy surface. The transition energies for a four-state adiabatic model are taken from the electronic difference spectrum resolved by pump-dump-probe transient absorption spectroscopy (see above & Fig. 5). For the hydrogen-bonding changes and structural motions that characterize the A_1 and A_2 states, the adiabatic criterion is assumed for picoseconds transfer processes between these states. In this model the A_1 resting state is refilled in the adiabatic limit as shown in Fig. 2. A double-well potential in the excited state lowers the potential energy of the additional well relative to the dark state geometry and is populated within ~200 fs after the pump interaction.

We performed a non-perturbative density matrix simulation that fully demonstrates the coherence dynamics using the essential experimental parameters. Using Wigner transforms[72] of the density matrix that evolves under the Liouville von Neumann equation the phase space distributions demonstrate the coherence progression and transfer in the respective pump and dump interactions. We demonstrate the measured dynamics by modelling a simplified 4-level density matrix simulation that uses a 170 cm^{-1} vibrational frequency and matches the electronic transition energy of the neutral trans chromophore. The first pump interaction prepares population transfer from the vibrational ground state of S_0 into the two lowest vibrational states of S_1 (i.e. $\rho_{00} \rightarrow \rho_{22} + \rho_{33}$) and creates ground state coherence between the vibrational states in S_0, i.e. ρ_{01} from interaction with the Boltzmann distributions of ρ_{00} and ρ_{11} as well as excited state coherence ρ_{23} using the experimental laser spectrum and duration. We introduce the Stokes field with a 350 fs pump-dump delay according to the experimental value and using the criterion $\Gamma_{electronic} > \frac{1}{\Delta t_{pump-dump}} > \Gamma_{vibrational}$ (see above). The carrier frequency couples the population transfers and efficiently transfers the vibrational coherence $\rho_{23} \rightarrow \rho_{01}$. The simulation fully demonstrates Tannor-Rice dynamics[73–75] in the pump-dump scheme by transfer of coherence. The experimental results follow the Tannor-Rice time domain approach where the initial ultrafast pulse creates an intermediate state that is subsequently transferred into the final state by the dump pulse. The time delay between the pulses is used to control the reaction[73–75]. In the Wigner phase space, the transforms that are separated for the ground and excited state elements show the momentum and position transfer to the ground state that is stimulated by the Stokes field. In the wavepacket picture, the transfer of position and momentum of excited to ground state will cause the wavepacket to continue motion towards the dissociative state following the dump pulse (Fig. 2). Strikingly the density matrix simulation for the PDP condition doubles the magnitude of the ground state coherence and adds strong support to the assignments for the PP and PDP crystallographic data. The ~5000 cm^{-1} relaxation term between absorption (λ_{max}=390 nm) and fluorescence (λ_{max}=490 nm) energies formally requires a 4-level electronic system. The number of Fock states included in an extended model determine the high order

structure of the Wigner transforms and therefore take assumptions in the number of vibrationally excited levels. The transfer of electronic state $|2\rangle$ to the redshifted radiative state $|1\rangle$ proceeds under a Landau-Zener reaction driven Hamiltonian. A pump-dump simulation in a complete system however does not alter the physics of the coherence transfer dynamics demonstrated already in the 4-level system which is fully sufficient to demonstrate the structural dynamics observed in the time resolved experiments together with optical control and are additionally generally applicable to other systems.

These simulations are appropriate demonstration for the dynamics observed in rsKiiro. There are unlikely to exist general conclusions with regard to assignment of ultrafast crystallographic differences. The ultrafast motion is determined by quantum dynamics of electronic ground and excited states, optical parameters, electronic and vibrational coherence and dephasing, mode displacements, multimode character and coupling, concentrations and heterogeneity. In addition the crystallographic differences are determined by ordering parameters, resolution and signal to noise statistics. The outcome of the experiment cannot be predicted because of the complex characteristics of the structural dynamics and the measurements. The optical control experiment ensures that the observations after the dump interaction are exclusively showing the electronic ground state motions.

This work shows that for the off state of rsKiiro the ultrafast motions of PP and PDP experiments belong to coherent dynamics in the electronic ground state. It should be emphasized that this conclusion is unrelated to other proteins that have been studied by TR-SFX. These include the Photoactive Yellow protein[76] (PYP), bacteriorhodopsin[77,78], rsEGFP2[31] and myoglobin[79], which all have very different photochemical, structural and quantum dynamics characteristics. This work however provides experimental methods and coherence modelling in order to aid the assignment and evaluation of functional significance of the motions observed on the ultrafast time scale

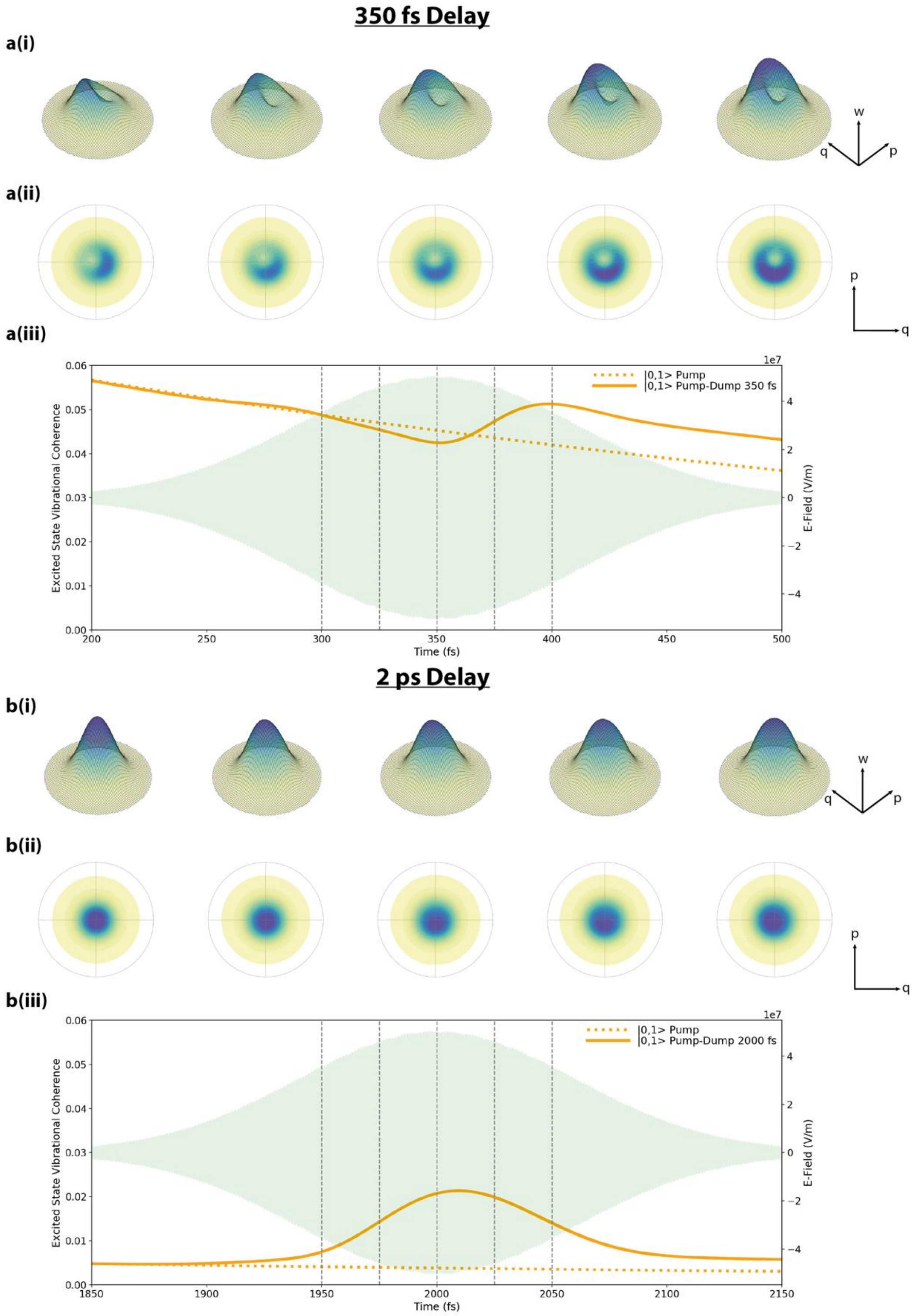

Supplementary Fig. 61| Ground state Wigner plots. Surface (i) and contour (ii) plots of Wigner functions of the ground electronic states for pump-dump delays of 350 fs (a) 2 ps (b). Vibrational coherence (off-diagonal elements) of the ground electronic state (|0,1>) (iii) for pump-dump delays of 350 fs (a) and 2 ps (b) , shown for pump (dotted) and pump-dump (solid) around the centre of the dump pulse, grey lines at 25 fs intervals depict time stamps of Wigner plots. Surface plot (i) colour schemes are normalized for each scheme to highlight asymmetric features.

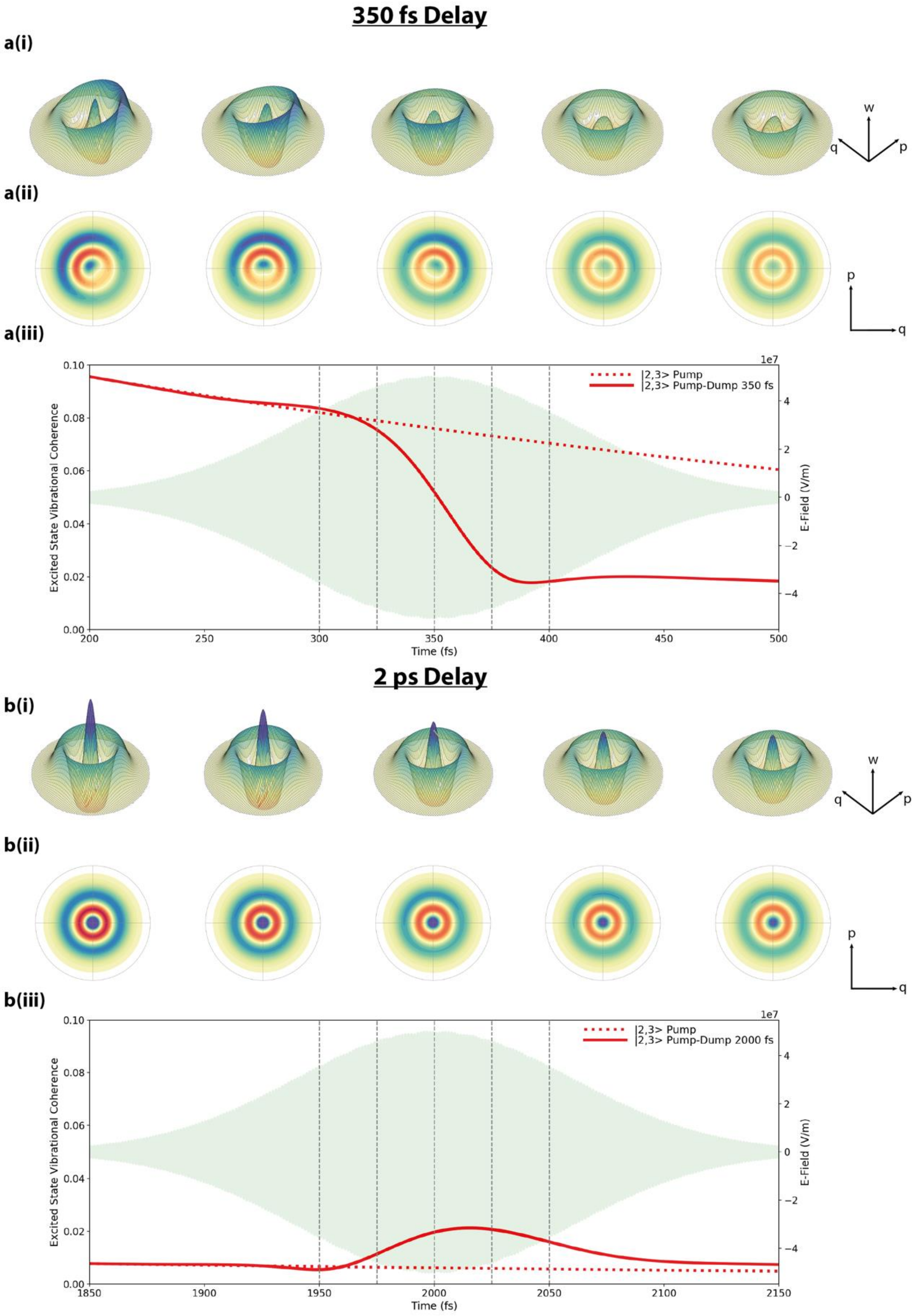

Supplementary Fig. 62| Excited state Wigner plots. Surface (i) and contour (ii) plots of Wigner functions of the excited electronic states for pump-dump delays of 350 fs (a) 2 ps (b). Vibrational coherence (off-diagonal elements) of the excited electronic state (|2,3>) (iii) for pump-dump delays of 350 fs (a) and 2 ps (b) , shown for pump (dotted) and pump-dump (solid) around the centre of the dump pulse, grey lines at 50 fs intervals depict time stamps of Wigner plots. Surface plot (i) colour schemes are normalized for each individual plot to highlight asymmetric features.

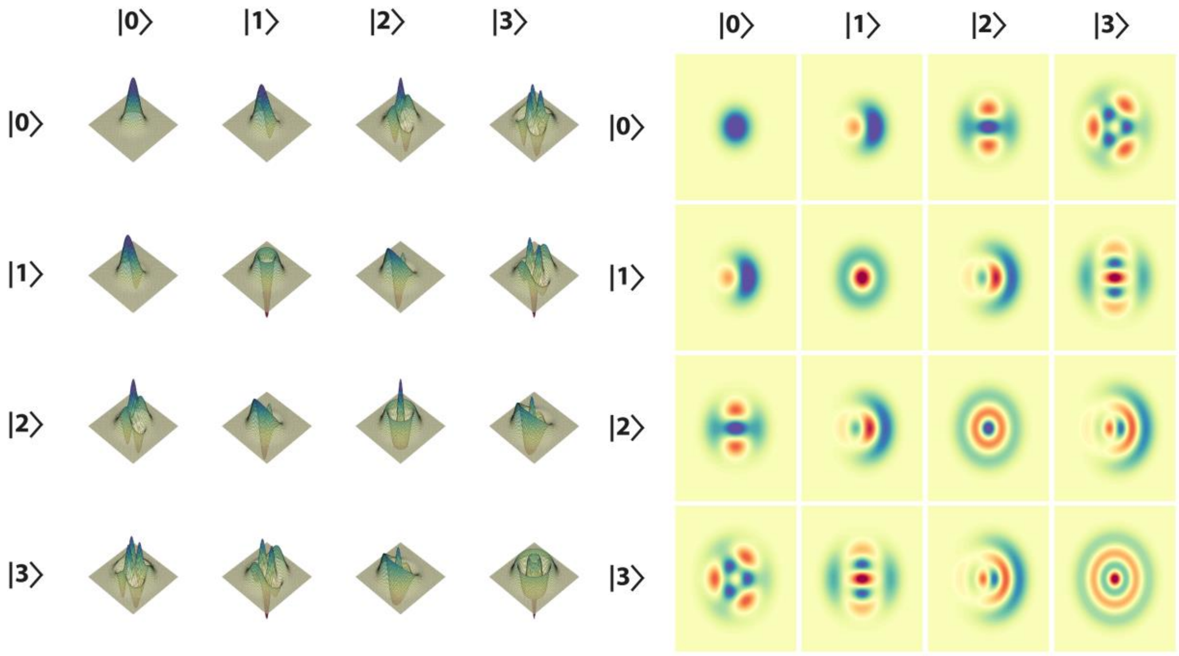

Supplementary Fig. 63 | Reference Wigner Plots. Surface (left) and contour plots (right) of normalized Wigner functions of various combinations of Fock states. Diagonal plots are pure states while off diagonal are mixed states with coherences present. The number of lines of symmetry in a Wigner plot is equal to the number difference between those Fock states. In the contour plots blue denotes a positive Wigner probability and red negative.

14. Supplementary references

1. Gahl, C. *et al.* A femtosecond X-ray/optical cross-correlator. *Nat. Photonics* **2**, 165–169 (2008).

2. Sanchez-gonzalez, A. *et al.* Coincidence timing of femtosecond optical pulses in an X-ray free electron laser. *J. Appl. Phys.* 203105 (2017) doi:10.1063/1.5012749.

3. Trebino, R. *et al.* Measuring ultrashort laser pulses in the time-frequency domain using frequency-resolved optical gating. *Rev. Sci. Instrum.* **68**, 3277–3295 (1997).

4. Linden, S., Giessen, H. & Kuhl, J. XFROG - A new method for amplitude and phase characterization of weak ultrashort pulses. *Phys. Status Solidi Basic Res.* **206**, 119–124 (1998).

5. Lincoln, C. N., Fitzpatrick, A. E. & Thor, J. J. V. Photoisomerisation quantum yield and non-linear cross-sections with femtosecond excitation of the photoactive yellow protein. *Phys. Chem. Chem. Phys.* **14**, 15752–15764 (2012).

6. Šrajer, V. *et al.* Protein Conformational Relaxation and Ligand Migration in Myoglobin: A Nanosecond to Millisecond Molecular Movie from Time-Resolved Laue X-ray Diffraction. *Biochemistry* **40**, 13802–13815 (2001).

7. Ursby, T. & Bourgeois, D. Improved Estimation of Structure-Factor Difference Amplitudesfrom Poorly Accurate Data. *Acta Crystallogr. Sect. A Found. Crystallogr.* **53**, 564–575 (1997).

8. Pandey, S. *et al.* Time-resolved serial femtosecond crystallography at the European XFEL. *Nat. Methods* **17**, 73–78 (2020).

9. Gorel, A., Schlichting, I. & Barends, T. R. M. Discerning best practices in XFEL-based biological crystallography – standards for nonstandard experiments. *IUCrJ* **8**, 532–543 (2021).

10. Tickle, I. *et al.* STARANISO. at http://staraniso.globalphasing.org/cgi-bin/staraniso.cgi (2018).

11. Prokhorenko, V. I., Halpin, A., Johnson, P. J. M., Miller, R. J. D. & Brown, L. S. Coherent control of the isomerization of retinal in bacteriorhodopsin in the high intensity regime. *J. Chem. Phys.* **134**, 085105 (2011).

12. Hutchison, C. D. M. *et al.* Photocycle populations with femtosecond excitation of crystalline photoactive yellow protein. *Chem. Phys. Lett.* **654**, 63–71 (2016).

13. Romei, M. G., Lin, C. Y., Mathews, I. I. & Boxer, S. G. Electrostatic control of photoisomerization pathways in proteins. *Science (80-.).* **367**, 76–79 (2020).

14. Fluorescence Decay Analysis Software 1.4, FlourTools. at www.fluortools.com/software/decayfit (2014).

15. Cotlet, M. *et al.* Excited-State Dynamics in the Enhanced Green Fluorescent Protein Mutant Probed by Picosecond Time-Resolved Single Photon Counting Spectroscopy. *J. Phys. Chem. B* **105**, 4999–5006 (2001).

16. Jung, G., Wiehler, J. & Zumbusch, A. The Photophysics of Green Fluorescent Protein: Influence of the Key Amino Acids at Positions 65, 203, and 222. *Biophys. J.* **88**, 1932–1947 (2005).

17. Ghosh, A. *et al.* Quantifying Microsecond Transition Times Using Fluorescence Lifetime Correlation Spectroscopy. *J. Phys. Chem. Lett.* **8**, 6022–6028 (2017).

18. Flors, C. *et al.* A stroboscopic approach for fast photoactivation-localization microscopy with Dronpa mutants. *J. Am. Chem. Soc.* **129**, 13970–13977 (2007).

19. Morozov, D. & Groenhof, G. Hydrogen Bond Fluctuations Control Photochromism in a Reversibly Photo-Switchable Fluorescent Protein. *Angew. Chemie Int. Ed.* **55**, 576–578 (2016).

20. Interactive chart of FP properties. http://www.fpbase.org/chart/.

21. Würth, C., Grabolle, M., Pauli, J., Spieles, M. & Resch-Genger, U. Relative and absolute determination of fluorescence quantum yields of transparent samples. *Nat. Protoc.* **8**, 1535–1550 (2013).

22. Gaigalas, A. K. & Wang, L. Measurement of the fluorescence quantum yield using a spectrometer with an integrating sphere detector. *J. Res. Natl. Inst. Stand. Technol.* **113**, 17 (2008).

23. Strickler, S. J. & Berg, R. A. Relationship between Absorption Intensity and Fluorescence Lifetime of Molecules. *J. Chem. Phys.* **37**, 814–822 (1962).

24. Hutchison, C. *et al.* X-ray Free Electron Laser Determination of Crystal Structures of Dark and Light States of a Reversibly Photoswitching Fluorescent Protein at Room Temperature. *Int. J. Mol. Sci.* **18**, 1918 (2017).

25. Zhang, X. *et al.* Highly photostable, reversibly photoswitchable fluorescent protein with high contrast ratio for live-cell superresolution microscopy. *Proc. Natl. Acad. Sci.* 201611038 (2016) doi:10.1073/pnas.1611038113.

26. Close, D. W. *et al.* Thermal green protein, an extremely stable, nonaggregating fluorescent protein created by structure-guided surface engineering. *Proteins Struct. Funct. Bioinforma.* **83**, 1225–1237 (2015).

27. Pletnev, S., Subach, F. V., Dauter, Z., Wlodawer, A. & Verkhusha, V. V. A Structural Basis for Reversible Photoswitching of Absorbance Spectra in Red Fluorescent Protein rsTagRFP. *J. Mol. Biol.* **417**, 144–151 (2012).

28. Grotjohann, T. *et al.* rsEGFP2 enables fast RESOLFT nanoscopy of living cells. *Elife* **1**, (2012).

29. Kaucikas, M. *et al.* Room temperature crystal structure of the fast switching M159T mutant of the fluorescent protein dronpa. *Proteins Struct. Funct. Bioinforma.* **83**, 397–402 (2015).

30. Woodhouse, J. *et al.* Photoswitching mechanism of a fluorescent protein revealed by time-resolved crystallography and transient absorption spectroscopy. *Nat. Commun.* **11**, 1–11 (2020).

31. Coquelle, N. *et al.* Chromophore twisting in the excited state of a photoswitchable fluorescent protein captured by time-resolved serial femtosecond crystallography. *Nat. Chem.* **10**, 31–37 (2018).

32. Colletier, J. P. *et al.* Serial Femtosecond Crystallography and Ultrafast Absorption Spectroscopy of the Photoswitchable Fluorescent Protein IrisFP. *J. Phys. Chem. Lett.* **7**, 882–887 (2016).

33. Pilling, M. J. & Robertson, S. H. Master Equation Models For Chemical Reactions Of Importance In Combustion. *Annu. Rev. Phys. Chem.* **54**, 245–275 (2003).

34. Summers, D. & Scott, J. M. W. Systems of first-order chemical reactions. *Math. Comput. Model.* **10**, 901–909 (1988).

35. Lin, C.-Y., Both, J., Do, K. & Boxer, S. G. Mechanism and bottlenecks in strand photodissociation of split green fluorescent proteins (GFPs). *Proc. Natl. Acad. Sci.* **114**, E2146–E2155 (2017).

36. Nitzan, A. *Chemical Dynamics in Condensed Phases Relaxation, Transfer and Reactions in*

Condensed Molecular Systems. (Oxford University Press, 2006).

37. Breuer, H.-P. & Petruccione, F. *The Theory of Open Quantum Systems*. (Oxford University Press, 2002).

38. Bochenkova, A. V. & Andersen, L. H. Ultrafast dual photoresponse of isolated biological chromophores: link to the photoinduced mode-specific non-adiabatic dynamics in proteins. *Faraday Discuss.* **163**, 297 (2013).

39. Kim, J. E., Tauber, M. J. & Mathies, R. A. Analysis of the Mode-Specific Excited-State Energy Distribution and Wavelength-Dependent Photoreaction Quantum Yield in Rhodopsin. *Biophys. J.* **84**, 2492–2501 (2003).

40. Wittig, C. The Landau−Zener Formula †. *J. Phys. Chem. B* **109**, 8428–8430 (2005).

41. Alvarez-Idaboy, J. R., Mora-Diez, N. & Vivier-Bunge, A. A Quantum Chemical and Classical Transition State Theory Explanation of Negative Activation Energies in OH Addition To Substituted Ethenes. *J. Am. Chem. Soc.* **122**, 3715–3720 (2000).

42. Krasnoperov, L. N., Peng, J. & Marshall, P. Modified Transition State Theory and Negative Apparent Activation Energies of Simple Metathesis Reactions: Application to the Reaction CH 3 + HBr → CH 4 + Br †. *J. Phys. Chem. A* **110**, 3110–3120 (2006).

43. Mozurkewich, M. & Benson, S. W. Negative activation energies and curved Arrhenius plots. 1. Theory of reactions over potential wells. *J. Phys. Chem.* **88**, 6429–6435 (1984).

44. Agmon, N. Kinetics of Switchable Proton Escape from a Proton-Wire within Green Fluorescence Protein. *J. Phys. Chem. B* **111**, 7870–7878 (2007).

45. Frauenfelder, H. *et al.* A unified model of protein dynamics. *Proc. Natl. Acad. Sci.* **106**, 5129–5134 (2009).

46. Nagel, Z. D., Dong, M., Bahnson, B. J. & Klinman, J. P. (Suppl. Mat.) Impaired protein conformational landscapes as revealed in anomalous Arrhenius prefactors. *Proc. Natl. Acad. Sci.* **108**, 10520–10525 (2011).

47. Englman, R. & Jortner, J. The energy gap law for radiationless transitions in large molecules. *Mol. Phys.* **18**, 145–164 (1970).

48. Seidner, L. & Domcke, W. Microscopic modelling of photoisomerization and internal-conversion dynamics. *Chem. Phys.* **186**, 27–40 (1994).

49. Donahue, N. M. Reaction Barriers: Origin and Evolution. *Chem. Rev.* **103**, 4593–4604 (2003).

50. Marcus, R. A. Foreword: Beyond the Historical Perspective on Hydrogen and Electron Transfers. in *Quantum Tunnelling in Enzyme-Catalysed Reactions* (eds. Allemann, R. K. & Scrutton, N. S.) (RSC Publishing, 2009).

51. Nitzan, A. & Jortner, J. Non radiative transition probabilities in the statistical limit. *Theor. Chim. Acta* **30**, 217–229 (1973).

52. Litvinenko, K. L., Webber, N. M. & Meech, S. R. Internal Conversion in the Chromophore of the Green Fluorescent Protein: Temperature Dependence and Isoviscosity Analysis. *J. Phys. Chem. A* **107**, 2616–2623 (2003).

53. Duan, Y. *et al.* A point-charge force field for molecular mechanics simulations of proteins based on condensed-phase quantum mechanical calculations. *J. Comput. Chem.* **24**, 1999–2012 (2003).

54. Sousa da Silva, A. W. & Vranken, W. F. ACPYPE - AnteChamber PYthon Parser interfacE. *BMC Res. Notes* **5**, 367 (2012).

55. Pronk, S. *et al.* GROMACS 4.5: a high-throughput and highly parallel open source molecular simulation toolkit. *Bioinformatics* **29**, 845–854 (2013).

56. Titov, A. V., Ufimtsev, I. S., Luehr, N. & Martinez, T. J. Generating Efficient Quantum Chemistry Codes for Novel Architectures. *J. Chem. Theory Comput.* **9**, 213–221 (2013).

57. Adamo, C. & Barone, V. Toward reliable density functional methods without adjustable parameters: The PBE0 model. *J. Chem. Phys.* **110**, 6158–6170 (1999).

58. Dunning, T. H. Gaussian basis sets for use in correlated molecular calculations. I. The atoms boron through neon and hydrogen. *J. Chem. Phys.* **90**, 1007–1023 (1989).

59. Grimme, S., Antony, J., Ehrlich, S. & Krieg, H. A consistent and accurate ab initio parametrization of density functional dispersion correction (DFT-D) for the 94 elements H-Pu. *J. Chem. Phys.* **132**, 154104 (2010).

60. Hirata, S. & Head-Gordon, M. Time-dependent density functional theory within the Tamm–Dancoff approximation. *Chem. Phys. Lett.* **314**, 291–299 (1999).

61. Granovsky, A. A. Extended multi-configuration quasi-degenerate perturbation theory: The new approach to multi-state multi-reference perturbation theory. *J. Chem. Phys.* **134**, 214113 (2011).

62. Granovsky, A. A. Firefly version 8. at http://classic.chem.msu.su/gran/firefly/index.html.

63. Brühl, E., Buckup, T. & Motzkus, M. Experimental and numerical investigation of a phase-only control mechanism in the linear intensity regime. *J. Chem. Phys.* **148**, 214310 (2018).

64. Wohlleben, W., Buckup, T., Herek, J. L. & Motzkus, M. Coherent Control for Spectroscopy and Manipulation of Biological Dynamics. *ChemPhysChem* **6**, 850–857 (2005).

65. Johansson, J. R., Nation, P. D. & Nori, F. QuTiP: An open-source Python framework for the dynamics of open quantum systems. *Comput. Phys. Commun.* **183**, 1760–1772 (2012).

66. Leonhardt, U. & Paul, H. Measuring the quantum state of light. *Prog. Quantum Electron.* **19**, 89–130 (1995).

67. Fujisawa, T., Kuramochi, H., Hosoi, H., Takeuchi, S. & Tahara, T. Role of Coherent Low-Frequency Motion in Excited-State Proton Transfer of Green Fluorescent Protein Studied by Time-Resolved Impulsive Stimulated Raman Spectroscopy. *J. Am. Chem. Soc.* **138**, 3942–3945 (2016).

68. Kumar, A. T. N., Rosca, F., Widom, A. & Champion, P. M. Investigations of amplitude and phase excitation profiles in femtosecond coherence spectroscopy. *J. Chem. Phys.* **114**, 701 (2001).

69. Kumar, A. T. N., Rosca, F., Widom, A. & Champion, P. M. Investigations of ultrafast nuclear response induced by resonant and nonresonant laser pulses. *J. Chem. Phys.* **114**, 6795–6815 (2001).

70. Hutchison, C. D. M. & van Thor, J. J. Populations and coherence in femtosecond time resolved X-ray crystallography of the photoactive yellow protein. *Int. Rev. Phys. Chem.* **36**, 117–143 (2017).

71. Mukamel, S. *Principles of Nonlinear Optical Spectroscopy*. (Oxford University Press, 1995).

72. Wigner, E. On the Quantum Correction For Thermodynamic Equilibrium. *Phys. Rev.* **40**, 749–759

(1932).

73. Tannor, D. J. & Rice, S. A. Control of selectivity of chemical reaction via control of wave packet evolution. *J. Chem. Phys.* **83**, 5013–5018 (1985).

74. Tannor, D. J., Kosloff, R. & Rice, S. A. Coherent pulse sequence induced control of selectivity of reactions: Exact quantum mechanical calculations. *J. Chem. Phys.* **85**, 5805–5820 (1986).

75. Kosloff, R., Rice, S. A., Gaspard, P., Tersigni, S. & Tannor, D. J. Wavepacket dancing: Achieving chemical selectivity by shaping light pulses. *Chem. Phys.* **139**, 201–220 (1989).

76. Pande, K. *et al.* Femtosecond structural dynamics drives the trans/cis isomerization in photoactive yellow protein. *Science (80-.).* **352**, 725–729 (2016).

77. Nogly, P. *et al.* Retinal isomerization in bacteriorhodopsin captured by a femtosecond x-ray laser. *Science (80-.).* **361**, eaat0094 (2018).

78. Nass Kovacs, G. *et al.* Three-dimensional view of ultrafast dynamics in photoexcited bacteriorhodopsin. *Nat. Commun.* **10**, 3177 (2019).

79. Barends, T. R. M. *et al.* Direct observation of ultrafast collective motions in CO myoglobin upon ligand dissociation. *Science (80-.).* **350**, 445–450 (2015).
